# *In crystallo* lattice adaptivity triggered by solid-gas reactions of cationic group 7 pincer complexes†

**DOI:** 10.1039/d3cc03201a

**Published:** 2023-08-07

**Authors:** Joe C. Goodall, M. Arif Sajjad, Emily A. Thompson, Samuel J. Page, Adam M. Kerrigan, Huw T. Jenkins, Jason M. Lynam, Stuart A. Macgregor, Andrew S. Weller

**Affiliations:** a Department of Chemistry, University of York York YO10 5DD UK; b Institute of Chemical Sciences, Heriot-Watt University Edinburgh EH14 4AS UK; c Department of Chemistry, University of Durham Durham DH1 3LE UK; d The York-JEOL Nanocentre, University of York, Heslington York YO10 5BR UK

## Abstract

The group 7 complexes [M(κ^3^-2,6-(R_2_PO)_2_C_5_H_3_N)(CO)_2_L][BAr^F^_4_] [M = Mn, R = ^*i*^Pr, L = THF; M = Re, R = ^*t*^Bu, L = vacant site] undergo *in crystallo* solid-gas reactivity with CO to form the products of THF substitution or CO addition respectively. There is a large, local, adaptive change of [BAr^F^_4_] anions for M = Mn, whereas for M = Re the changes are smaller and also remote to the site of reactivity.

## Abstract 2

*In crystallo* solid/gas reactions of cationic group 7 pincer complexes reveal large adaptive responses of the partnering [BAr^F^_4_]^−^ anions in response to changes at the metal centre.

## Graphical abstract

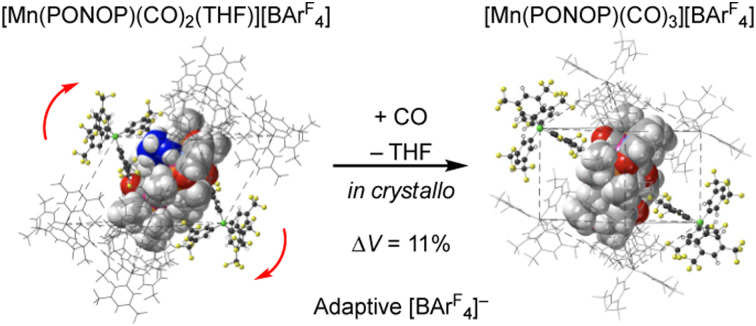

Molecular single crystals that are adaptive^1^ to external stimuli are promising materials for applications in organic electronics,^2^ actuating devices^3^ or catalysis.^4^ Stimuli can be mechanical, photochemical, thermal or chemical;^5^ and can lead to crystal deformation (either *restorative* or *disintegrative*^5*c*^), changes in crystalline phase, or changes in chemical/electronic properties. While many factors can control adaptive responses,^1,5*a*,*c*^ fluorous groups appear to be important in promoting single-crystal to single crystal (SC–SC) transformations.^6^

Organometallic reactivity *in crystallo* allows for highly reactive complexes that are often inaccessible, or very short lived, in solution to be synthesised, characterised and undergo onward reactivity in the solid state.^7^ We have been developing the synthesis, catalysis and structural analysis of cationic group 9 complexes using SC–SC methods, calling this Solid-state Molecular OrganoMetallic Chemistry (SMOM).^8^ This approach allows for relatively stable σ-alkane complexes to be isolated by the solid/gas hydrogenation of precursor alkene complexes,^9^ or the observation of different reaction pathways in solid/gas reactivity compared with solution.^8,10,11^ These systems are exemplified by a reactive cation that sits in a cavity formed from [BAr^F^_4_]^−^, or related,^12^ anions [Ar^F^ = 3,5-(CF_3_)_2_C_6_H_3_], which are often arranged in ∼octahedral (*O*_h_) or ∼bicapped square prismatic (BCSP) motifs. Fig. 1 shows examples of each motif in selected rhodium σ-alkane complexes.^9^ These arrangements provide a 3° periodic framework, with associated unit cell volume changes of <2% on reaction, and 2° non-covalent interactions, that collectively retain crystallinity, stabilise the 1° metal cation site,^9,13^ and promote reactant ingress/product egress,^14^ selectivity in ligand binding,^15^ and reactivity.^8,16^ While much focus has been on the metal cation, structural changes associated with the anion motif have been less studied,^4,10^ and include decomposition routes in which the [BAr^F^_4_]^−^ anion coordinates to the metal centre and crystallinity is lost.^9^

**Fig. 1:**
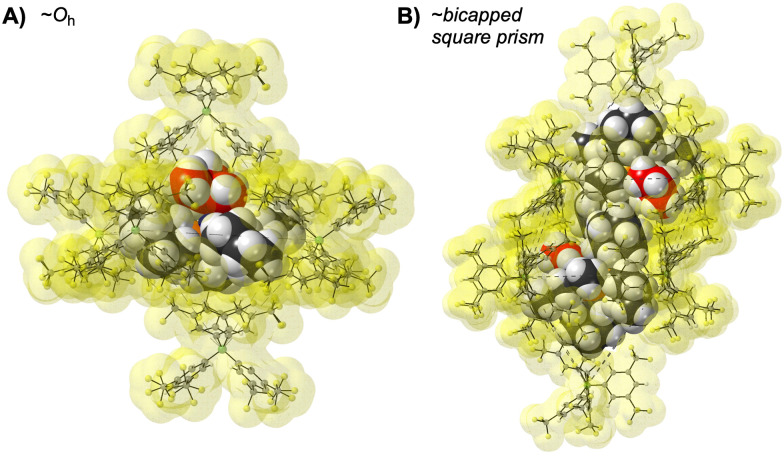
Common motifs of [BAr^F^_4_]^−^ anions in SMOM (A) [Rh(Cy_2_PCH_2_CH_2_PCy_2_)(isobutane)][BAr^F^_4_]; (B) [Rh(Cy_2_PCH_2_CH_2_PCy_2_)(hexane)][BAr^F^_4_].

We now report that by extending the SMOM methodology to group 7 pincer cations, [M(R-PONOP)(CO)_2_L][BAr^F^_4_], Scheme 1 [M = Mn, R = ^*i*^Pr, L = THF; M = Re, R = ^*t*^Bu, L = vacant site; R-PONOP = κ^3^-2,6-(R_2_PO)_2_C_5_H_3_N)], solid/gas reactions with CO at the metal site can result in significant adaption of the anion framework in response to changes at the metal centre. The mechanical stresses associated with such reactions result in significant fracturing of the crystals, and MicroED methods^12,17^ are used to analyse resulting microcrystals. Computational studies offer insight into the changes in inter-ion interactions that are associated with these rearrangements.

**Scheme 1:**
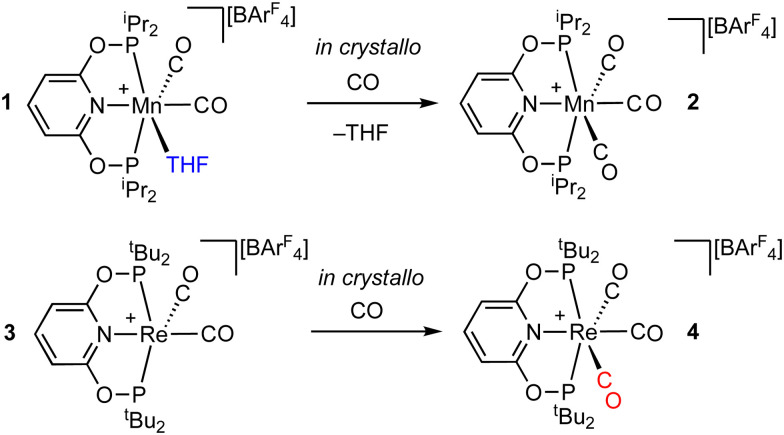
*In crystallo* reactivity reported in this work.

While the solution-phase chemistry of Mn and Re pincer complexes is well established,^18^ examples of SC–SC reactivity with group 7 complexes is limited.^19^ Our recent report of SC–SC reactivity using [Ir(^*i*^Pr-PONOP)(propene)][BAr^F^_4_],^10^ suggested that the R–PONOP ligand could act to template the [BAr^F^_4_]^−^ anions in group 7 complexes, potentially resulting in a favourable motif for *in crystallo* reactivity. New starting complexes were targeted in which CO could be subsequently added in a solid/gas reaction, either to displace a weakly bound THF ligand, [Mn(^*i*^Pr-PONOP)(CO)_2_(THF)][BAr^F^_4_] 1, or added at a vacant site, *i.e.* 16-electron [Re(^*t*^Bu-PONOP)(CO)_2_][BAr^F^_4_], 3. These complexes were synthesised in good yield as analytically pure crystalline materials, and fully characterised by solution (CD_2_Cl_2_) and solid-state (SS) NMR spectroscopy (ESI†). The solid-state structure of 1 (Fig. 2A), as determined by single-crystal X-ray diffraction (110 K) shows the THF ligand bound *trans* to CO [Mn–C1, 1.781(5) Å]. The [BAr^F^_4_]^−^ anions form a distorted BCSP motif in which two crystallographically identical [Mn(^*i*^Pr-PONOP)(CO)_2_(THF)]^+^ cations are enclosed by 10 anions, with the cyclic THF ligand sitting in a cleft^13,16^ of two Ar^F^ groups from a proximal [BAr^F^_4_]^−^ anion, Fig. 2B. There is 0.5 of hexane per unit cell (not shown). Complex 3 also shows a BCSP anion motif. The 16-electron Re centre has one relatively short distance to C22 [3.122(4) Å], and a slightly compressed Re–P2–C20 angle compared to the opposite Re–P1–C7 angle [107.83(13)° *cf.* 117.49(14)°]. However, there is no evidence for an agostic interaction from QTAIM studies or ^1^H NMR spectroscopy (at 193 K), and this distortion towards the vacant site likely arises from steric pressure with a proximal [BAr^F^_4_]^−^ anion.^20^ DFT calculations support this, with optimisation of the isolated 16e cation resulting in relaxation of both Re–P–C angles to 113.9° while Re⋯C22 lengthens to 3.43 Å. The observation of a single CO environment in the solution ^13^C{^1^H} NMR spectrum (*δ* 198.8, 298 K, CD_2_Cl_2_) suggests a dynamic process that gives time-averaged *C*_2v_ symmetry. DFT calculations modelling this in solution confirm a rocking motion that interconverts two equivalent square-pyramidal structures with a barrier of only 3.4 kcal mol^−1^. In contrast, the 298 K single-crystal X-ray structure shows no positional disorder that would signal the presence of such an alternative isomer (Fig. S41, ESI†); while periodic-DFT calculations show the alternative square-pyramidal structure is 16 kcal mol^−1^ higher when computed within the unit cell of 3.

**Fig. 2:**
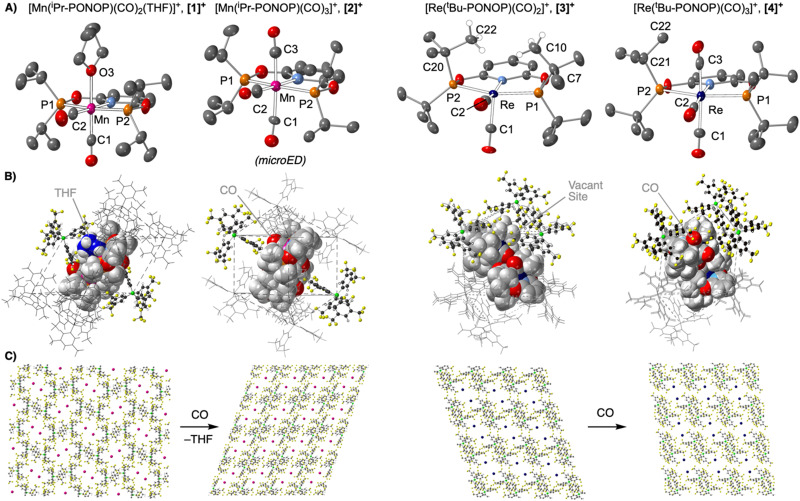
(A) Single crystal X-ray diffraction and MicroED structures of complexes 1, 2, 3 and 4. Selected H-atoms shown. Displacement ellipsoids shown at the 40% probability level. (B) Extended solid-state structures showing the two cations (van der Waals radii) in a ∼ BCSP of [BAr^F^_4_] anions. Selected anions shown as ball & stick representation. (C) Packing motifs of the [BAr^F^_4_] anions with Mn and Re centres shown as spheres of arbitrary radius.

Addition of CO (1 bar, 5 days) to crystals of 1 resulted in the formation of [Mn(^*i*^Pr-PONOP)(CO)_3_][BAr^F^_4_], 2. The reaction can be followed visually by the colour change from bright yellow (1) to colourless (2), exemplified in Fig. 3A and B using large crystals. This is a *disintegrative in crystallo* reaction, and internal shattering of the crystals occurs so that no material remained that was suitable for analysis by single crystal X-ray diffraction, despite repeating on a range of crystal sizes (0.1 mm^3^ to 2 mm^3^). SEM images before and after reaction with CO show significant fracturing on the μm scale (Fig. 3D and E). Following this transformation by ^31^P{^1^H} SSNMR spectroscopy showed a clean transition between 1 and 2, with no intermediate phase observed. These ^31^P{^1^H} SSNMR spectra of 1 and 2 show well-defined, but complex, multiplets, reflecting ^55^Mn–^31^P (^55^Mn *I* = 5/2, 100% abundant) and *trans*^31^P–^31^P coupling for two crystallographically inequivalent environments (Fig. 3C).^21^ Consistent with surface area effects, finely ground microcrystals react faster with CO to form 2 (28 hours). This microcrystalline material was analysed using MicroED^12,17*a*^ methods, by merging 8 independent data sets (95.3% completeness, 0.83 Å resolution) collected from regions at the edges of fractured microcrystals of ∼1 μm^3^. The molecular structure of the cation in 2 (Fig. 2A and B) confirms the substitution of THF for CO. This ligand exchange in the primary coordination sphere triggers the proximal [BAr^F^_4_]^−^ anion that enfolded the THF ligand in 1 to pivot so that one of its aryl groups now points directly towards the new CO ligand.

**Fig. 3:**
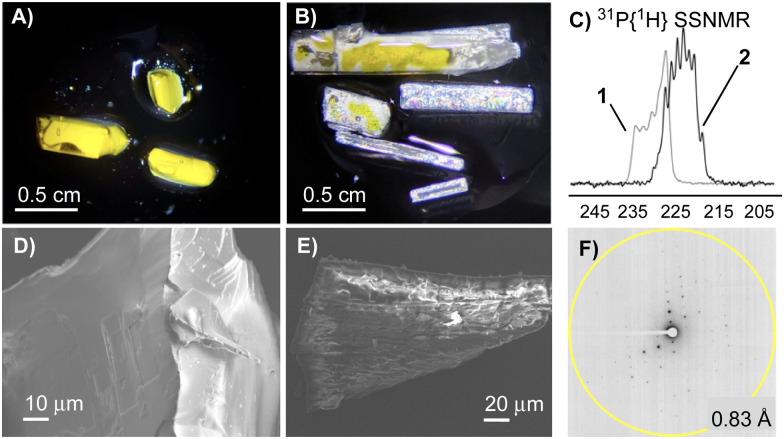
Optical images of large crystals of 1 before (A) and during (B) CO addition to form 2 (48 hours); (C) ^31^P{^1^H} SSNMR of complexes 1 and 2. SEM images of complex 1 (D) and 2 (E); (F) electron diffraction pattern of 2.

This lattice adaptation results in a change in space group, loss of THF and lattice hexane, and a considerable decrease in the unit cell volume of ∼11% (1: *P*2_1_/*n*, 6306 Å^3^, *Z* = 4; 2: *P*1̄ 2836 Å^3^, *Z* = 2, Table S1, ESI†). While the overall ∼BCSP motif of local [BAr^F^_4_]^−^ anions is retained, the cations have moved from a herringbone pattern in 1 to a parallel arrangement in 2, Fig. 2C. While this large structural change likely results in mechanical stress and fracturing of the crystals on a macroscopic scale, we propose that the fluorous groups in the [BAr^F^_4_]^−^ anion promote sufficient plasticity in the lattice to retain micro-crystallinity. Large changes in unit cell volumes have been reported previously in SC–SC transformations.^5*d*,5*e*^

A related, but less extreme, adaptive response comes from addition of CO (1 bar, 24 h) to bright-red 16-electron 3 to form colourless [Re(^*t*^Bu-PONOP)(CO)_3_][BAr^F^_4_], 4. While this reaction also results in shattering of the crystals, this is less severe than observed for 1/2, and crystalline material suitable for single-crystal X-Ray diffraction remained. The resulting analysis shows that the BCSP motif was retained, but compared with 1 the change in unit cell was far more modest (0.7%, 3016 Å^3^*vs.* 3035 Å^3^), there was no change in space group (*P*1̄), and there is no major reorientation of the [BAr^F^_4_]^−^ anions. However, coordination of the CO to the vacant site does result in the ^*t*^Bu group that was in close approach to the Re-centre becoming more open, Re–P2–C21 119.4(4)°. The resulting steric pressure on the local [BAr^F^_4_]^−^ anions causes a slight reorganisation of the motif, as reflected by the B⋯B distances on the BCSP cap changing [3 : 9.214(8)–14.4679(2); 4 : 9.134(12)–14.1715(3) Å]. ^31^P{^1^H} SSNMR spectroscopy shows that 3 to 4 is quantitative.

Periodic-DFT calculations (PBE-D3) were used to analyse the structural changes associated with THF/CO substitution in the 1/2 pair. First, a proto-structure for 2, 2*, was optimised using the structure and unit cell parameters of 1 with THF replaced by CO at each Mn centre. 2* is 66.0 kcal mol^−1^ less stable than 2 when the latter is optimised within its experimental unit cell; there is therefore a strong thermodynamic driving force for lattice rearrangement. To assess changes in the 2° microenvironment between 1 and 2*, individual ion-pair energies were computed between one of the Mn cations within the BCSP motif and each of its five nearest-neighbour [BAr^F^_4_]^−^ anions. The PBE functional was used, both with and without a D3 correction, to allow dispersion effects to be quantified. For each ion-pair IGMH analyses (Independent Gradient Model; Hirshfeld partitioning) highlighted the most important inter-ion non-covalent interactions (see the ESI† for full details).

Fig. 4 illustrates these analyses for the ion-pair (IP1) which enfolds the THF ligand in 1 that is then substituted by CO to form 2*. The computed ion-pair energy in 1 is 64.4 kcal mol^−1^, of which 13.3 kcal mol^−1^ is due to dispersion. The IGMH isosurface shows a green swathe of dispersive stabilisation between the THF and the two proximate Ar^F^ groups of the [BAr^F^_4_]^−^ anion. Within this the colour-coded δ*G*^atom^ values indicate the most important contributions come from one THF methylene group. A similar pattern has been noted in Rh σ-alkane complexes.^13^ Additional disk-like features correspond to weak C–H⋯F H-bonds between the PONOP ^*i*^Pr substituents and anion CF_3_ groups. After THF/CO substitution these C–H⋯F interactions are the only significant features that remain in IP1 in 2*. The ion-pair energy is reduced by 16.3 kcal mol^−1^ compared to 1, of which 6.7 kcal mol^−1^ (*ca.* 40%) is due to dispersion; the remainder presumably reflects changes in inter-ion electrostatic interactions. For the remaining ion-pairs the computed changes in ion-pair energies between 1 and 2* are much smaller (Fig. S47, ESI†) and when summed across all five ion-pairs the total change is +17.6 kcal mol^−1^. IP1 contributes >92% of this, suggesting this ion-pair dominates the 1/2 structural transformation.

**Fig. 4:**
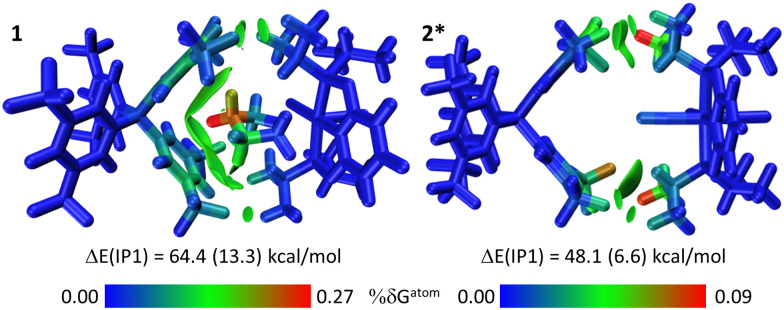
IGMH plots for IP1 in 1 and its equivalent in 2* after THF/CO substitution. Cations and anions are defined as separate fragments; sign(*λ*_2_)ρ-coloured isosurfaces are plotted with δ*G*^inter^ = 0.003 a.u; relative atomic contributions coloured by %δ*G*^atom^. The ion-pair interaction energies are also indicated (kcal mol^−1^) with the contribution from dispersion in brackets. See text for details.

A similar analysis on the 3/4 transformation optimised a proto-structure 4* after adding CO to each Re centre in the unit cell of 3. 4* is 15.0 kcal mol^−1^ less stable than 4 optimised in its experimental unit cell. Lattice rearrangement is again favoured, but with a lower thermodynamic driving force than the 1/2 pair, consistent with the smaller 3/4 structural change. The change in the summed ion-pair energies between 3 and 4* is also much smaller (+3.3 kcal mol^−1^, Fig. S48, ESI†) of which IP1, the ion-pair adjacent to the added CO ligand, contributes −5.4 kcal mol^−1^. Instead, the largest contribution involves the movement of the PONOP ^*t*^Bu substituent within the pocket of an adjacent [BAr^F^_4_]^−^ anion (IP2, Δ*E* = +7.3 kcal mol^−1^).

In conclusion, we show that the [BAr^F^_4_]^−^ anions in SMOM systems can be remarkably adaptive in response to changes at the metal centre arising from solid/gas reactions. The largest changes are observed for the 1/2 pairing, being dominated by changes to IP1, the site of THF/CO substitution, and extensive crystal degradation occurs. For 3/4 the ion-pair changes are more balanced and smaller structural changes occur. We are currently exploring the extent to which the analysis of 2° microenvironment effects in proto-structures, such as 2* and 4*, can be used as a predictive tool of *in crystallo* reactivity.

The EPSRC (EP/W015552, EP/W015498, DTP for J. C. G.), Wellcome Trust (206161/Z/17/Z); ARCHER2 UK National Supercomputing Service, Royal Society (J. M. L. INF\R1\221057).

## Conflicts of interest

There are no conflicts to declare.

## Supplementary Material

CC-059-D3CC03201A-s001

CC-059-D3CC03201A-s002
