# Supplementary material for: In crystallo lattice adaptivity triggered by solid-gas reactions of cationic group 7 pincer complexes
Source: Chem Commun (Camb). 2023 Aug 7;59(72):10749–52. doi: 10.1039/d3cc03201a (PMC10484290; doi:10.1039/d3cc03201a)
Supplement: CC-059-D3CC03201A-s001 [file CC-059-D3CC03201A-s001.pdf]

Electronic Supplementary Material (ESI) for ChemComm.
This journal is © The Royal Society of Chemistry 2023

Electronic Supporting Information for:

In Crystallo Lattice Adaptivity Triggered by Solid-Gas Reactions of Cationic Group 7 Pincer Complexes

Joe C. Goodall,[a] M. Arif Sajjad,[b] Emily A. Thompson,[a] Samuel J. Page,[c] Adam M. Kerrigan,[d] Huw T. Jenkins,[a*] Jason M. Lynam,[a*] Stuart A. Macgregor[b*] and Andrew S. Weller[a*]

[a]Department of Chemistry, University of York, York, YO10 5DD (UK); email: andrew.weller@york.ac.uk; jason.lynam@york.ac.uk; huw.jenkins@york.ac.uk

[b]Institute of Chemical Sciences, Heriot-Watt University, Edinburgh, EH14 4AS (UK); email: s.a.macgregor@hw.ac.uk

[c]Department of Chemistry, University of Durham, South Road, Durham, DH1 3LE (UK)

[d]York JEOL Nanocentre, Helix House, Innovation Way, Heslington, York, YO10 5BR (UK)

Contents

S1. Experimental Procedures **2**
S1a. General Considerations 2
S1b. Synthesis 3
S1c. NMR Spectra 9
S1d. IR Spectra 32
S2. MicroED Methods **38**
S3. Single-Crystal X-Ray Diffraction **39**
S3a. Room Temperature Structure of [Re(tBu-PONOP)(CO)2][BArF4] 39
S3b. Crystallographic Data Tables 40
S4. SEM Methods **43**
S5. Computational Section **44**
S5a. Computational Details 44
S5ai. Periodic DFT Calculations 44
S5aii. Computational Details for Molecular DFT Calculations 44
S5b. QTAIM Analysis of the [Re(tBu-PONOP)(CO)2]+ molecular cation 45
S5c. Comparison of experimental and computed structures of 1-4. 46
S5d. IGMH Plots 50
S6. References **54**

S1. Experimental Procedures

S1a. General Considerations

All manipulations, unless otherwise stated, were performed under standard Schlenk line and glovebox (<0.5 ppm O_2/H_2O) techniques under an argon (BOC, N4.8 purity), carbon monoxide (CK Gases, N3.7 purity) or nitrogen atmosphere. Glassware was dried overnight at 140 °C and flame dried under vacuum before use. Pentane, hexane, CH_2Cl_2 and toluene were dried using a commercially available Grubbs-type purification system (Innovative Technology) and degassed with three freeze-pump-thaw cycles and stored over 3 Å molecular sieves (CH_2Cl_2 and toluene) under argon in resealable glass ampoules fitted with PTFE high vacuum stopcocks (Rotaflo HP or J. Young). C_6H_5F (pre-treated with alumina), 1,2-$C_6H_4F_2$ (pre-treated with alumina) and CD_2Cl_2 were dried over CaH_2, vacuum transferred, degassed with three freeze-pump-thaw cycles, and stored over 3 Å molecular sieves. Tetrahydrofuran (THF) was dried over Na/benzophenone, distilled, degassed with three freeze-pump-thaw cycles and stored over 3 Å molecular sieves. Na[$BAr^F{}_4$],[1] ^{i}Pr-PONOP,[2] ^{t}Bu-PONOP,[3] [Mn(^{i}Pr-PONOP)Br$(CO)_2$][4] and ReBr$(CO)_5$[5] were synthesised according to literature methods. All other chemicals were from commercial sources and used without further purification. Solution state NMR data were collected on a Bruker AVIIIHD 500 MHz or AVIIIHD 600 MHz Widebore spectrometer at the temperatures specified. The solution ^{1}H and ^{13}C{^{1}H} NMR spectra were referenced to the residual solvent peaks. Assignments were aided with ^{1}H{^{31}P} solution NMR data. ^{31}P{^{1}H} and ^{11}B{^{1}H} solution spectra were referenced externally to 85% H_3PO_4 in D_2O and 5% BF_3•OEt_2 in C_6D_6, respectively. Solid-state NMR samples were prepared in an argon-filled glovebox by pre-loading 60-100 mg of crushed material into a 4.0 mm zirconia solid-state NMR rotor and sealed with Kel-F, vespel or zirconia caps. Solid-state NMR data were obtained on Bruker Avance III HD spectrometers, operating at 100.63 MHz (^{13}C{^{1}H}), 100.56 MHz (^{13}C{^{1}H}), 162.04 MHz (^{31}P{^{1}H}), 161.99 MHz (^{31}P{^{1}H}) or 376.5 MHz (^{19}F{^{1}H}) at the MAS rates and temperatures specified. All ^{13}C{^{1}H} CP MAS spectra were referenced to adamantane where the upfield methane resonance was taken to be δ_C = 29.5 ppm, secondarily referenced to δ_C($SiMe_4$) = 0.0 ppm. ^{31}P{^{1}H} CP MAS spectra were referenced to triphenylphosphine (δ_P = -9.3 ppm relative to H_3PO_4) or calcium hydrogen phosphate (δ_P = 1.4 ppm relative to H_3PO_4). ^{19}F{^{1}H} HPDEC MAS spectra were referenced to CF_3COOH/H_2O (50% v/v, δ -76.54 ppm relative to $CFCl_3$). Solid-state NMR spectra were recorded at varied MAS rates to determine isotropic chemical shifts. Electrospray ionisation mass spectrometry (ESI-MS) was carried out using a Bruker MicrOTOF instrument by Mr Karl Heaton at the University of York. ESI-MS for [Mn(^{i}Pr-PONOP)(THF)$(CO)_2$][$BAr^F{}_4$] was collected using a modified glovebox directly attached to a Bruker HCT-II ion trap. Elemental microanalyses were performed by Dr Graeme McAllister at the University of York, or by Ms Orfhlaith McCullough at London Metropolitan University.

S1b. Synthesis

Preparation of [Mn(^{i}Pr-PONOP)(CO)$_3$][BAr$^F{}_4$]

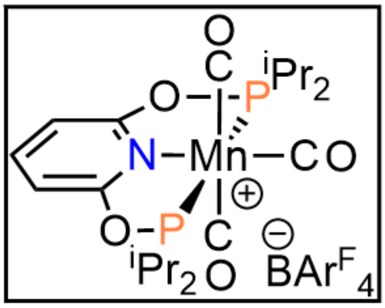

An ampoule was charged with [Mn(^{i}Pr-PONOP)Br(CO)$_2$] (283 mg) and Na[BAr$^F{}_4$] (512 mg). Fluorobenzene (30 mL) was vacuum transferred onto the solids. The ampoule was backfilled with CO (1 bar gauge) at 78 K, warmed to room temperature and stirred for 18 hours. The reaction mixture became cloudy during this time, presumably due to NaBr precipitation. The solution was degassed and the product precipitated by addition of pentane (200 mL). The product was isolated *via* cannula filtration. The product was extracted into CH_2Cl_2 (20 mL), cannula filtered, then precipitated with pentane (200 mL). The solids were washed with pentane (3 x 10 mL) and dried *in vacuo* to yield the complex as a microcrystalline white powder (536 mg, 75% yield). Crystals suitable for single-crystal X-Ray diffraction were obtained by layering a CH_2Cl_2 solution with pentane at room temperature.

^{1}H Solution NMR (600 MHz, CD_2Cl_2, 298 K): δ 7.78 (t, $^2J_{HH}$ = 8.1 Hz, 1 H, *para*-PONOP), 7.72 (s, 8 H, *ortho*-BAr$^F{}_4$), 7.56 (s, 4 H, *para*-BAr$^F{}_4$), 6.85 (d, $^2J_{HH}$ = 8.0 Hz, 2 H, *meta*-PONOP), 3.00 (virtual m, 4 H, PC***H***Me$_2$), 1.47 (virtual dd, 12 H, PCHC***H***$_3$), 1.37 (virtual dd, 12 H, PCHC***H***$_3$).

^{1}H{^{31}P} Solution NMR (600 MHz, CD_2Cl_2, 298 K): δ 7.77 (t, $^2J_{HH}$ = 7.9 Hz, 1 H, *para*-PONOP), 7.71 (s, 8 H, *ortho*-BAr$^F{}_4$), 7.56 (s, 4 H, *para*-BAr$^F{}_4$), 6.85 (d, $^2J_{HH}$ = 7.4 Hz, 2 H, *meta*-PONOP), 3.00 (br sept, $^2J_{HH}$ = 7.0 Hz, 4 H, PC***H***Me$_2$), 1.47 (d, $^2J_{HH}$ = 6.0 Hz, 12 H, PCHC***H***$_3$), 1.37 (d, $^2J_{HH}$ = 6.9 Hz, 12 H, PCHC***H***$_3$).

^{31}P{^{1}H} Solution NMR (242.95 MHz, CD_2Cl_2, 298 K): δ 225.2 (br s, fwhm = 165 Hz).

^{13}C{^{1}H} Solution NMR (150.91 MHz, CD_2Cl_2, 298 K): δ 217.2 (br, equatorial ***C***O), 213.3 (br, axial ***C***O), 162.9 (virtual t, *ortho*-PONOP), 162.2 (q, $^1J_{BC}$ = 50 Hz, *ipso*-BAr$^F{}_4$), 145.6 (s, *meta*-PONOP), 135.2 (s, *ortho*-BAr$^F{}_4$), 129.3 (qq, $^2J_{CF}$ = 32 Hz, $^4J_{CF}$ = 3 Hz, *meta*-BAr$^F{}_4$), 125.0 (q, $^1J_{CF}$ = 271 Hz, *C*F$_3$), 117.9 (sept., $^3J_{CF}$ = 4 Hz, *para*-BAr$^F{}_4$), 105.4 (s, *para*-PONOP), 33.0 (virtual t, P***C***H(CH$_3$)$_2$), 17.2 (s, PCH(***C***H$_3$)), 17.0 (s, PCH(***C***H$_3$)).

^{11}B{^{1}H} Solution NMR (192.53 MHz, CD_2Cl_2, 298 K): δ -6.6 (s, BAr$^F{}_4$).

^{31}P{^{1}H} CP SSNMR (162.06 MHz, 10 kHz spin rate, 298 K): δ 227.5, 226.1, 224.7, 223.2, 221.8, 220.5, 218.5.

^{13}C{^{1}H} CP SSNMR (100.66 MHz, 10 kHz spin rate, 298 K): δ 164.7, 163.68, 146.5, 138.4, 135.6, 134.6, 132.2, 131.5, 130.5, 125.9, 120.1, 119.3, 106.4, 103.8 (BAr$^F{}_4$ and PONOP environments), 38.5, 27.8 (methine), 19.7, 17.9, 17.4 (^{i}Pr methyl groups). CO resonance was not observed.

IR (ATR) *v*CO 2055 (w, s), 1970, (br, s) cm^{-1}.

ESI-MS m/z (CH_2Cl_2) found (calculated) for $C_{20}H_{31}MnNO_5P_2$ 482.1066 (482.1058).

Elemental analysis calc for $C_{52}H_{43}BF_{24}MnNO_5P_2$: C 46.42; H 3.22; N 1.04; found: C 46.34; H 3.17; N 1.12.

Preparation of [Mn(^{i}Pr-PONOP)(THF)(CO)$_2$][BAr$^F{}_4$]•½ hexane (1)

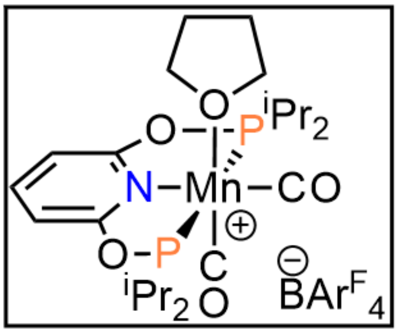

Tetrahydrofuran (THF, 5 mL) was added to an ampoule charged with [Mn(^{i}Pr-PONOP)(CO)$_3$][BAr$^F{}_4$] (233 mg) and trimethylamine N-oxide (15 mg). A deep orange solution formed immediately. The solution was stirred at room temperature for 1 hour. The volatiles were thoroughly removed under reduced pressure, the solids taken up in THF (2 mL), isolated *via* cannula filtration and layered with hexane at room temperature to yield the complex as bright orange needles (156 mg, 65% yield). *Note: [Mn(^{i}Pr-PONOP)(THF)(CO)$_2$][BAr$^F{}_4$] slowly decomposes into unidentified products at -30 °C in an argon glovebox over the course of months.*

^{1}H Solution NMR (600 MHz, CD_2Cl_2, 298 K): δ 7.86 (t, $^2J_{HH}$ = 8.0 Hz, 1 H, *para*-PONOP), 7.72 (br s, 8 H, *ortho*-BAr$^F{}_4$), 7.56 (s, 4 H, *para*-BAr$^F{}_4$), 6.92 (d, $^2J_{HH}$ = 8.2 Hz, 2 H, *meta*-PONOP), 4.61-1.70 (extremely broad, 18 H, fluxional THF exchange and overlapping methine environments), 1.48 (virtual m, 12 H, PCHMeC***H***$_3$), 1.34 (broad virtual m, 11 H, PCHC***H***$_3$Me). *Note: Fluxionality of THF ligand and decomposition in CD_2Cl_2 solution hindered effective integration of ^{1}H spectrum.*

^{31}P{^{1}H} Solution NMR (242.95 MHz, CD_2Cl_2, 298 K): δ 231.0 (br s, fwhm = 221 Hz).

^{13}C{^{1}H} Solution NMR (150.91 MHz, CD_2Cl_2, 298 K): δ 225.7 (m, ***C***O), 164.5 (virtual t, *ortho*-PONOP), 162.2 (q, $^1J_{BC}$ = 50 Hz, *ipso*-BAr$^F{}_4$), 145.9 (s, *para*-PONOP), 135.2 (s, *ortho*-BAr$^F{}_4$), 129.2 (q, $^2J_{CF}$ = 31 Hz, *meta*-BAr$^F{}_4$), 125.0 (q, $^1J_{CF}$ = 271 Hz, *C*F$_3$), 117.9 (sept., $^3J_{CF}$ = 4 Hz, *para*-BAr$^F{}_4$), 105.3 (s, *meta*-PONOP), 32.0 (s, hexane), 29.5 (br s, methine), 23.1 (s, hexane), 17.3 (br s, PCH(***C***H$_3$)), 14.3 (s, hexane). Bound THF resonances not observed.

^{31}P{^{1}H} CP MAS SSNMR (162.06 MHz, 10 kHz spin rate, 298 K): δ 234.6, 232.6, 230.8, 227.4.

^{13}C{^{1}H} CP MAS SSNMR (100.66 MHz, 10 kHz spin rate, 298 K): δ 164.4, 158.9 (*ortho*-PONOP and *ipso*-BAr$^F{}_4$), 143.3, 136.4, 134.7, 132.3, 130.9, 130.2, 124.9, 118.6, 117.4 (overlapping PONOP and BAr$^F{}_4$ resonances), 104.4 (*meta*-PONOP), 76.8, 68.8, 65.4 (THF resonances), 29.8, 26.7, 25.2, 23.9 (methine resonances), 22.1, 17.9, 16.5, 15.7, 14.2, 12.8 (overlapping ^{i}Pr groups and hexane resonances). CO resonances not observed.

^{19}F{^{1}H} HPDEC MAS SSNMR (376.5 MHz, 20 kHz spin rate, 298 K): δ -63.3, -64.8.

IR (nujol) *v*CO 1963 (s), 1891 (s) cm^{-1}.

ESI-MS (THF) found (calculated) for $C_{19}H_{31}MnNO_4P_2$ 454.15 (454.11).

Elemental analysis calc for $C_{58}H_{58}BF_{24}MnNO_5P_2$: C 48.62; H 4.08; N 0.98; found: C 48.22; H 4.18; N 0.92.

Preparation of [Mn(^{i}Pr-PONOP)(CO)$_3$][BAr$^F{}_4$] (solid-state) (2)

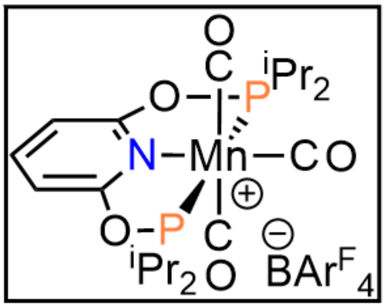

An ampoule was charged with finely ground [Mn(^{i}Pr-PONOP)(THF)(CO)$_2$][BAr$^F{}_4$]•½ hexane (50 mg), backfilled with CO (1 bar gauge) and sealed for 28 hours (optimised). The volatiles removed under reduced pressure to yield white microcrystalline [Mn(^{i}Pr-PONOP)(CO)$_3$][BAr$^F{}_4$] in quantitative yield.

^{1}H Solution NMR (600 MHz, CD_2Cl_2, 298 K): δ 7.81 (t, $^2J_{HH}$ = 8.2 Hz, 2 H, *para*-PONOP), 7.71 (s, 8 H, *ortho*-BAr$^F{}_4$), 7.55 (s, 4 H, *para*-BAr$^F{}_4$), 6.87 (d, $^2J_{HH}$ = 8.2 Hz, *meta*-PONOP), 3.68 (virtual m, 1 H, OC***H***$_2$), 3.01 (virtual m, 4 H, PC***H***Me$_2$), 1.82 (virtual m, 1 H, OCH$_2$C***H***$_2$), 1.48 (virtual dd, 12 H, PCHC***H***$_3$), 1.38 (virtual dd, 12 H, PCHC***H***$_3$).

^{31}P{^{1}H} Solution NMR (242.95 MHz, CD_2Cl_2, 298 K): δ 225.2 (br s, fwhm = 187 Hz).

^{13}C{^{1}H} Solution NMR (150.91 MHz, CD_2Cl_2, 298 K): δ 162.9 (virtual t, *ortho*-PONOP), 162.2 (q, $^1J_{BC}$ = 50 Hz, *ipso*-BAr$^F{}_4$), 145.7 (s, *meta*-PONOP), 135.2 (s, *ortho*-BAr$^F{}_4$), 129.3 (qq, $^2J_{CF}$ = 31 Hz, $^4J_{CF}$ = 3 Hz, *meta*-BAr$^F{}_4$), 125.0 (q, $^1J_{CF}$ = 272 Hz, *C*F$_3$), 117.9 (sept., $^3J_{CF}$ = 4 Hz, *para*-BAr$^F{}_4$), 105.4 (virtual t, *para*-PONOP), 68.2 (br s, O***C***H$_2$CH$_2$), 33.0 (virtual t, P*C*HMe$_2$), 26.0 (br s, OCH$_2$***C***H$_2$), 17.2 (s, PCH***C***H$_3$), 17.0 (s, PCH***C***H$_3$).

^{31}P{^{1}H} CP MAS SSNMR (162.06 MHz, 10 kHz spin rate, 298 K): δ 230.4, 227.5, 226.0, 224.6, 223.2, 221.8, 220.5, 218.6.

^{13}C{^{1}H} CP MAS SSNMR (100.66 MHz, 10 kHz spin rate, 298 K): δ 163.3 (*ortho*-PONOP and *ipso*-BAr$^F{}_4$), 145.1 (*meta*-PONOP), 137.1, 135.3, 133.9, 132.3, 130.6, 129.5, 124.8 (overlapping BAr$^F{}_4$ and PONOP resonances), 117.2, 104.5, 102.6 (BAr$^F{}_4$ and PONOP resonances) 67.3 (THF), 37.4, 36.3, 34.8 (methine resonances), 26.6 (THF), 25.3 (hexane), 18.5, 17.3, 16.5, 15.7, 14.0, 13.3 (^{i}Pr methyl and hexane resonances).

^{19}F{^{1}H} HPDEC MAS SSNMR (376.5 MHz, 20 kHz spin rate, 298 K): δ -59.5, -61.7, -63.4, -64.5, -65.0.

IR (ATR) ν(CO) 2057 (w, s), 1980 (s), 1947 (s) cm^{-1}

ESI-MS (CH_2Cl_2) found (calculated) for $C_{20}H_{31}MnNO_5P_2$ 482.1067 (482.1058).

Elemental Analysis calc for $C_{52}H_{43}BF_{24}MnNO_5P_2$: C 46.42; H 3.22; N 1.04; found: C 46.38; H 3.69; N 0.93.

Preparation of [Re(^{t}Bu-PONOP)Br(CO)$_2$]

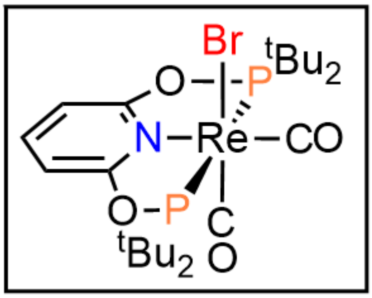

To an ampoule charged with $ReBr(CO)_5$ (490 mg) was added ^{t}Bu-PONOP (485 mg) in 1,2-difluorobenzene (10 mL). The reaction mixture was heated to 95 °C for 18 hours, turning from colourless to bright yellow. The reaction was cooled to room temperature and the volatiles removed under reduced pressure. The yellow solid was washed with pentane (3 x 10 mL), and extracted into diethyl ether (40 mL) and isolated *via* cannula filtration. The volatiles were removed under reduced pressure and dried *in vacuo* to yield a bright yellow microcrystalline solid. Yield: 607 mg (70%). Crystals suitable for single-crystal X-Ray diffraction were grown from slow vapour diffusion of pentane into a toluene solution at -30 °C.

^{1}H Solution NMR (500 MHz, C_6D_6, 298 K) δ 6.71 (t, $^2J_{HH}$ = 8.1 Hz, 1 H, *para*-PONOP), 6.14 (d, $^2J_{HH}$ = 8.1 Hz, 2 H, *meta*-PONOP), 1.66 (virtual t, 18 H, C(C***H***$_3$)$_3$), 1.32 (virtual t, 18 H, C(C*H*$_3$)$_3$).

^{31}P{^{1}H} Solution NMR (202.53 MHz, C_6D_6, 298 K) δ 196.0.

^{13}C{^{1}H} Solution NMR (125.80 MHz, C_6D_6, 298 K) δ 206.2 (s, equatorial ***C***O), 198.9 (s, axial ***C***O), 164.7 (virtual t, *ortho*-PONOP), 142.7 (s, *para*-PONOP), 102.6 (virtual t, *meta*-PONOP), 44.7 (virtual t, P*C*(CH$_3$)$_3$), 44.6 (virtual t, P***C***(CH$_3$)$_3$), 32.1 (virtual t, C(***C***H$_3$)$_3$), 28.0 (virtual t, C(*C*H$_3$)$_3$).

IR (ATR) νCO 1933 (s), 1842 (s).

ESI-MS m/z (CH_2Cl_2) found (calculated) for $C_{23}H_{39}NO_4P_2Re$ 642.1913 (642.1912) [M]$^+$-Br.

Elemental Analysis calc for $C_{23}H_{39}BrNO_4P_2Re$: C 38.28; H 5.45; N 1.94; found: C 38.58; H 5.63; N 1.91.

Preparation of [Re(^{t}Bu-PONOP)(CO)$_2$][BAr$^F{}_4$] (3)

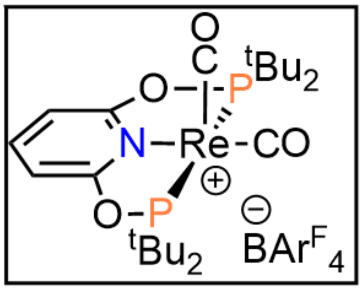

A solution of [Re(^{t}Bu-PONOP)Br(CO)$_2$] (400mg) in 1,2-difluorobenzene (10 mL) was added dropwise to a solution of Na[BAr$^F{}_4$] (567 mg) in 1,2-difluorobenzene (10 mL). After several drops of [Re(^{t}Bu-PONOP)Br(CO)$_2$] solution had been added, the reaction mixture rapidly changed from a cloudy white suspension to a bright red cloudy suspension. After complete addition, the reaction mixture was stirred at room temperature for 1 hour, then precipitated with hexane (200 mL). The reaction mixture was cannula filtered and the solids extracted into CH_2Cl_2 (15 mL). The solution was layered with hexane to yield the product as bright red block-like crystals. Yield: 670 mg (80%).

^{1}H Solution NMR (500 MHz, CD_2Cl_2, 298 K) δ 8.06 (t, $^2J_{HH}$ = 8.25 Hz, 1 H, *para*-PONOP), 7.73 (br m, 8 H, *ortho*-BAr$^F{}_4$), 7.56 (s, 4 H, *para*-BAr$^F{}_4$), 7.13 (d, $^2J_{HH}$ = 8.25 Hz, 2 H, *meta*-PONOP), 1.32 (virtual t, 36 H, C(C***H***$_3$)$_3$).

^{31}P{^{1}H} Solution NMR (202.53 MHz, CD_2Cl_2, 298 K) δ 214.4.

^{13}C{^{1}H} Solution NMR (125.80 MHz, CD_2Cl_2, 298 K) δ 198.8 (t, $^2J_{CP}$ = 5 Hz, ***C***O), 166.5 (virtual t, *ortho*-PONOP), 162.2 (q, $^1J_{BC}$ = 50 Hz, *ipso*-BAr$^F{}_4$), 148.8 (s, *para*-PONOP), 135.2 (s, *ortho*-BAr$^F{}_4$), 129.3 (qq, $^2J_{CF}$ = 32 Hz, $^3J_{CF}$ = 3 Hz, *meta*-BAr$^F{}_4$), 125.0 (t, $^1J_{CF}$ = 272 Hz, *C*F$_3$), 117.9 (sept., $^3J_{CF}$ = 4 Hz, *para*-BAr$^F{}_4$), 105.0 (virtual t, *meta*-PONOP), 43.8 (virtual t, P***C***(CH$_3$)), 27.5 (virtual t, C(***C***H$_3$)).

^{31}P{^{1}H} CP MAS SSNMR (162.02 MHz, 10 kHz spin rate, 298 K) δ 217.8 (v. broad), 214.5 (v. broad).

^{13}C{^{1}H} CP MAS SSNMR (100.63 MHz, 10 kHz spin rate, 298 K) 166.1-163.1 (*ortho*-PONOP and *ipso*-BAr$^F{}_4$), 147.5 (*para*-PONOP), 136.1, 134.9, 133.0, 130.4, 124.8, 117.9 (overlapping PONOP and BAr$^F{}_4$ resonances), 105.5, 102.6 (*meta*-PONOP), 47.6, 42.2, 41.2, 40.7 (P*C*(CH$_3$)$_3$), 26.7 (PC(*C*H$_3$)$_3$).

^{19}F{^{1}H} HPDEC MAS SSNMR (376.5 MHz, 20 kHz spin rate, 298 K): δ -62.5, -63.6.

IR (ATR) νCO 1965 (s), 1893 (s).

ESI-MS m/z (CH_2Cl_2) found (calculated) for $C_{23}H_{39}NO_4P_2Re$ 642.1904 (642.1912).

Elemental Analysis calc for $C_{55}H_{51}BF_{24}NO_4P_2Re$: C 43.90; H 3.42; N 0.93; found: C 44.09; H 3.62; N 0.92.

Preparation of [Re(^{t}Bu-PONOP)(CO)$_3$][BAr$^F{}_4$] (solid-state) (4)

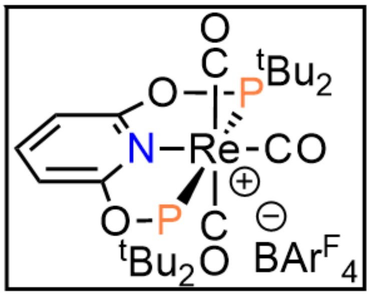

An ampoule was charged with crystalline [Re(^{t}Bu-PONOP)(CO)$_2$][BAr$^F{}_4$] (25 mg) and the ampoule backfilled with CO (1 bar gauge). The appearance of the red block-like crystals changed over 24 hours to white crystalline blocks with microcrystalline powder on the surface of the crystals. The ampoule was placed under vacuum and replaced with Ar (0.2 bar gauge). This process was repeated for a total of three vacuum/Ar cycles, yielding [Re(^{t}Bu-PONOP)(CO)$_3$][BAr$^F{}_4$] in quantitative yield.

^{1}H Solution NMR (500 MHz, CD_2Cl_2, 298 K) δ 7.86 (tt, $^2J_{HH}$ = 8.1 Hz, $^5J_{HP}$ = 0.8 Hz, 1 H, *para*-PONOP), 7.72 (br m, 8 H, *ortho*-BAr$^F{}_4$), 7.56 (s, 4 H, *para*-BAr$^F{}_4$), 6.95 (d, $^2J_{HH}$ = 8.2 Hz, 2 H, *meta*-PONOP), 1.49 (virtual t, 36 H, C(C***H***$_3$)$_3$).

^{31}P{^{1}H} Solution NMR (202.53 MHz, CD_2Cl_2, 298 K) δ 198.2.

^{13}C{^{1}H} Solution NMR (125.80 MHz, CD_2Cl_2, 298 K) δ 194.7 (t, $^2J_{CP}$ = 9 Hz, axial ***C***O), 192.7 (br s, equatorial ***C***O), 163.7 (s, *ortho*-PONOP), 162.2 (q, $^1J_{BC}$ = 50 Hz, *ipso*-BAr$^F{}_4$), 146.5 (s, *para*-PONOP), 135.2 (s, *ortho*-BAr$^F{}_4$), 129.3 (qq, $^2J_{CF}$ = 31 Hz, $^3J_{CF}$ = 3 Hz, *meta*-BAr$^F{}_4$), 125.0 (q, $^1J_{CF}$ = 272 Hz, *C*F$_3$), 117.9 (quint, $^3J_{CF}$ = 4 Hz, *para*-BAr$^F{}_4$), 105.2 (virtual t, *meta*-PONOP), 44.4 (virtual t, ***C***(CH$_3$)$_3$), 29.0 (virtual t, C(***C***H$_3$)$_3$).

^{31}P{^{1}H} CP MAS SSNMR (162.03 MHz, 10 kHz spin rate, 298 K): δ 200.3 (v. broad, FWHM = 2100 Hz).

^{13}C{^{1}H} CP MAS SSNMR (100.63 MHz, 10 kHz spin rate, 298 K): δ 163.6 (*ortho*-PONOP), 146.0 (*para*-PONOP), 136.2, 135.0, 133.9, 133.1, 131.0, 130.1 (overlapping BAr$^F{}_4$ resonances) 124.5 (CF_3), 119.2, 117.8, 116.6 (overlapping BAr$^F{}_4$ resonances), 105.0, 102.7(*meta*-PONOP resonances), 43.9, 42.9, 42.2 (***C***Me$_3$ resonances), 29.6, 29.1, 27.1, 26.5 (methyl resonances).

^{19}F{^{1}H} HPDEC MAS SSNMR (376.5 MHz, 20 kHz spin rate, 298 K): δ -61.8, -63.0, -64.3.

IR (ATR) νCO 2050 (s), 1956 (s), 1942 (s)

ESI-MS (CH_2Cl_2) found (calculated) for $C_{24}H_{39}NO_5P_2Re$ 670.1877 (670.1861).

S1c. NMR Spectra

[Mn(^{i}Pr-PONOP)(CO)$_3$][BAr$^F{}_4$]

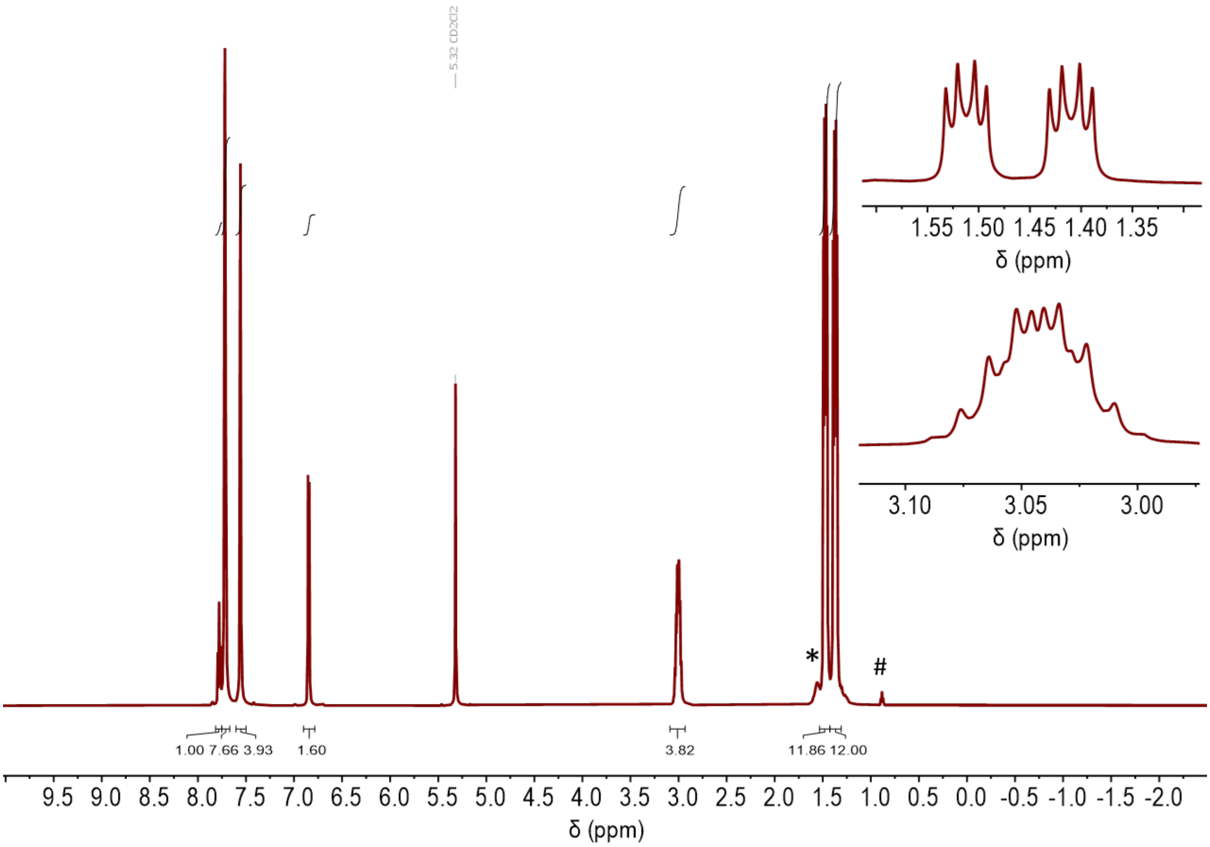

Figure S1: ^{1}H NMR spectrum of [Mn(^{i}Pr-PONOP)(CO)$_3$][BAr$^F{}_4$] (600 MHz, CD_2Cl_2, 298 K). Insets depict methine (3.04 ppm) and ^{i}Pr methyl groups (1.53-1.37 ppm). * denotes unknown trace impurity, # denotes pentane impurity.

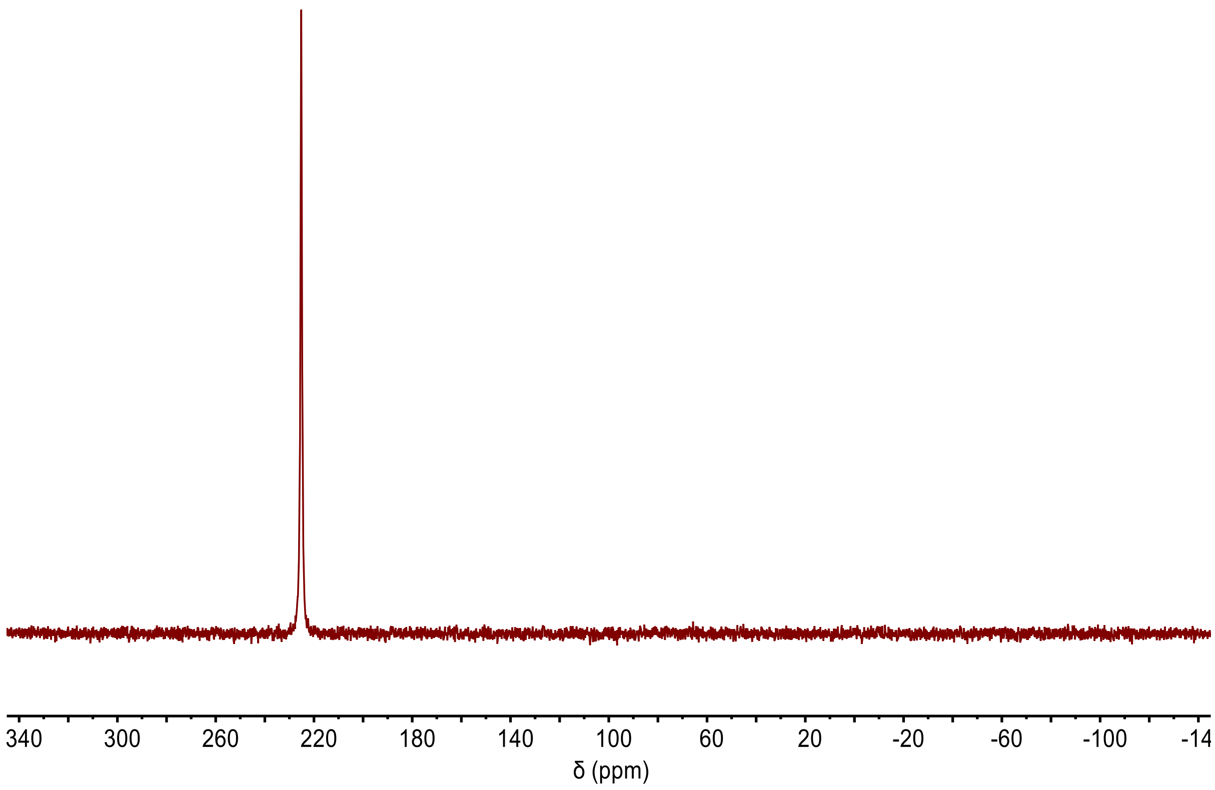

Figure S2: $^{31}P\{^{1}H\}$ NMR spectrum of [Mn(^{i}Pr-PONOP)(CO)$_3$][BAr$^{F}_{4}$] (600 MHz, CD_2Cl_2, 298 K).

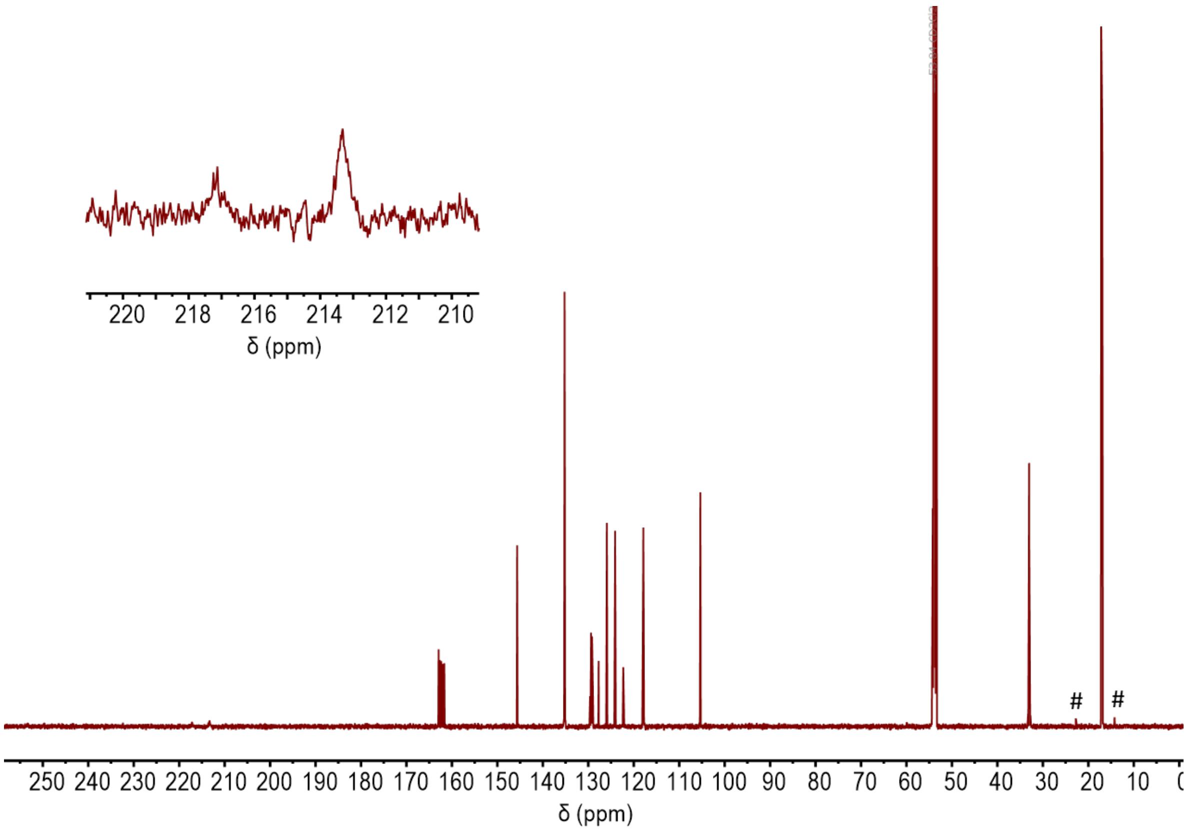

Figure S3: $^{13}C\{^{1}H\}$ NMR spectrum of [Mn(^{i}Pr-PONOP)(CO)$_3$][BAr$^{F}_{4}$] (150.91 MHz, CD_2Cl_2, 298 K). $^{\#}$ denotes pentane impurity.

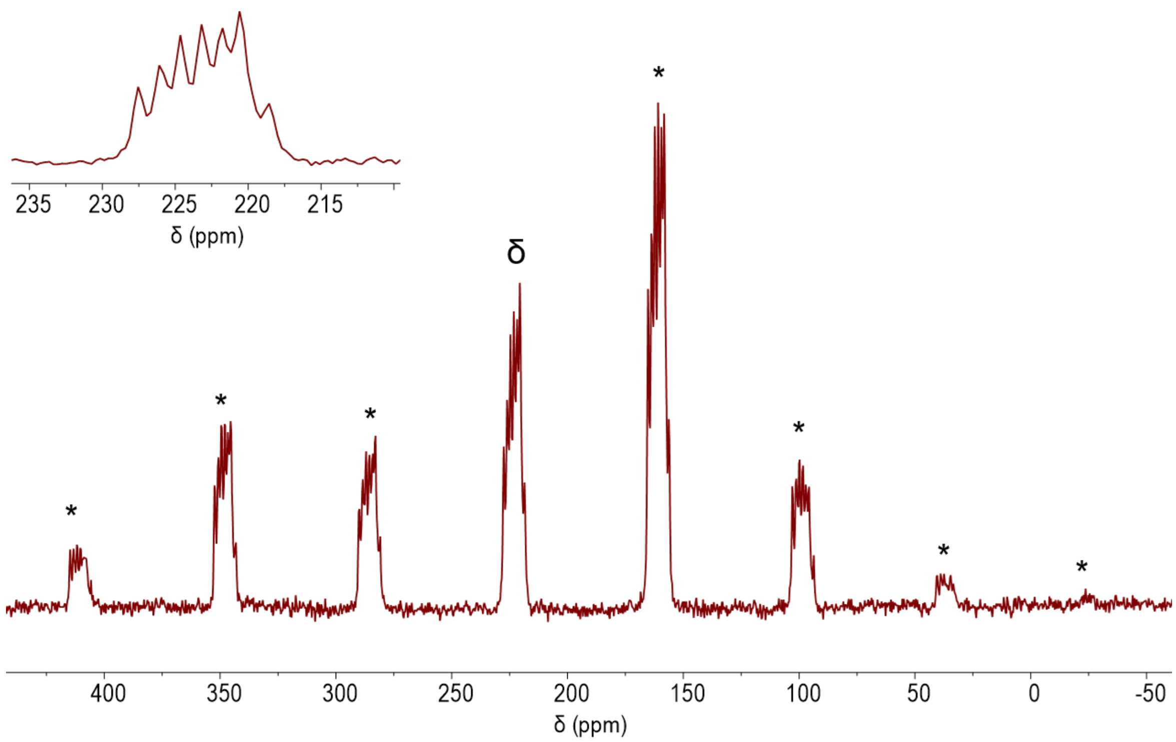

Figure S4: Solid-state $^{31}P\{^{1}H\}$ CP MAS spectrum of [Mn(^{i}Pr-PONOP)(CO)$_3$][BAr$^{F}{}_4$] (162.02 MHz, 10 kHz spin rate, 298 K). Inset depicts fine coupling of isotropic chemical shift. δ denotes isotropic chemical shift. * denotes spinning side bands.

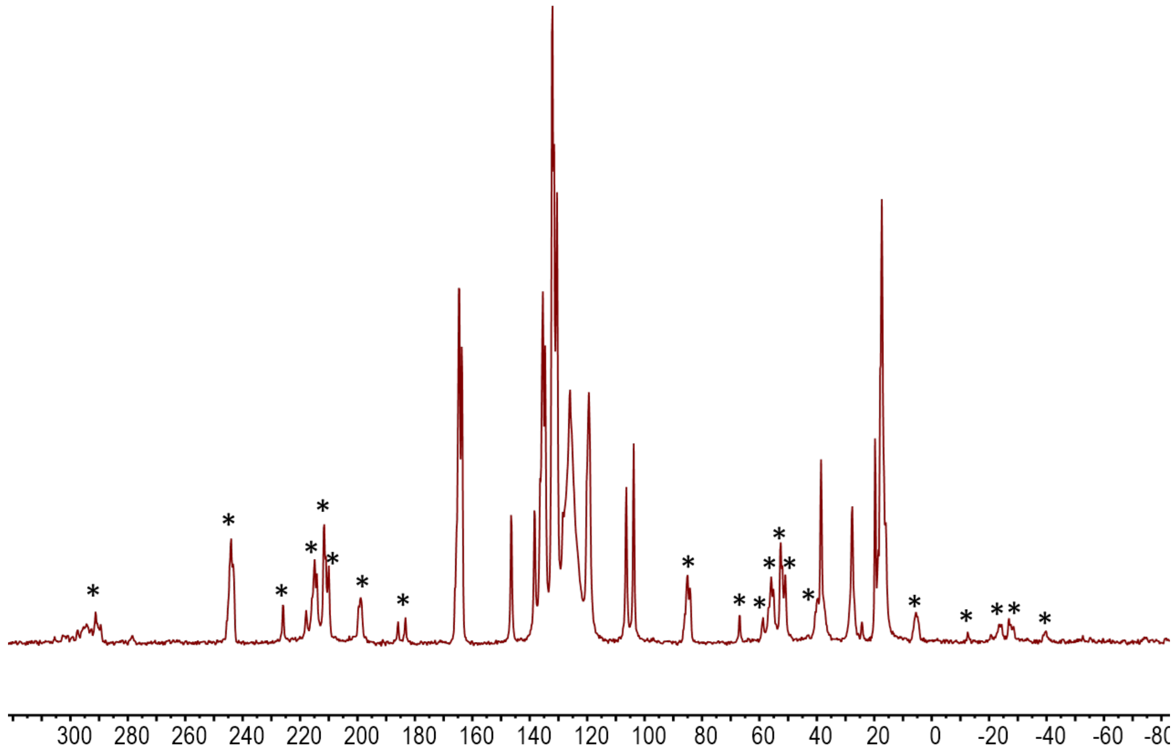

Figure S5: Solid-state $^{13}C\{^{1}H\}$ CP MAS spectrum of [Mn(^{i}Pr-PONOP)(CO)$_3$][BAr$^{F}{}_4$] (100.66 MHz, 10 kHz spin rate, 298 K). * denotes spinning side bands.

[Mn(^{i}Pr-PONOP)(CO)$_2$(THF)][BAr$^F_4$]

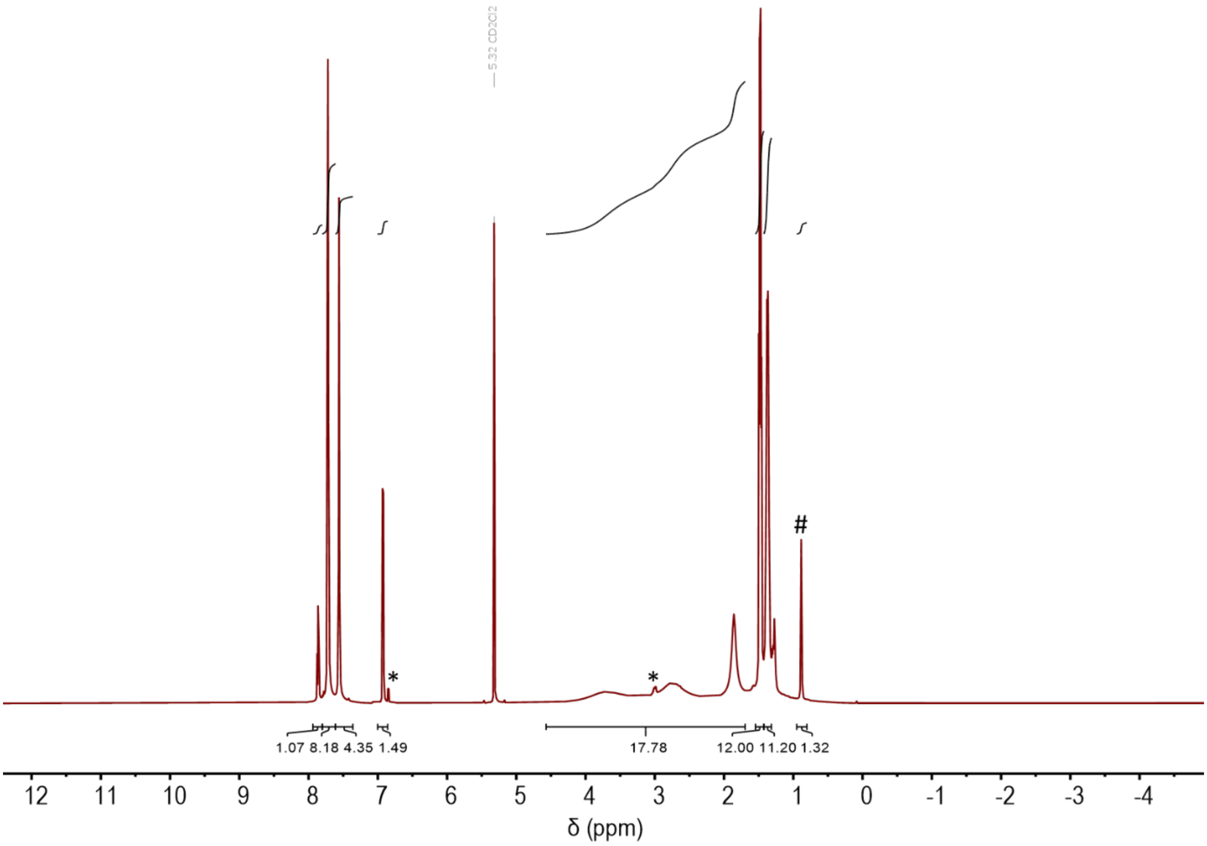

Figure S6: Solution ^{1}H NMR spectrum of [Mn(^{i}Pr-PONOP)(CO)$_2$(THF)][BAr$^F_4$] (600 MHz, CD_2Cl_2, 298 K). * denotes [Mn(^{i}Pr-PONOP)(CO)$_3$][BAr$^F_4$] which forms through CO scavenged decomposition in CD_2Cl_2 solution. # denotes co-crystallised hexane.

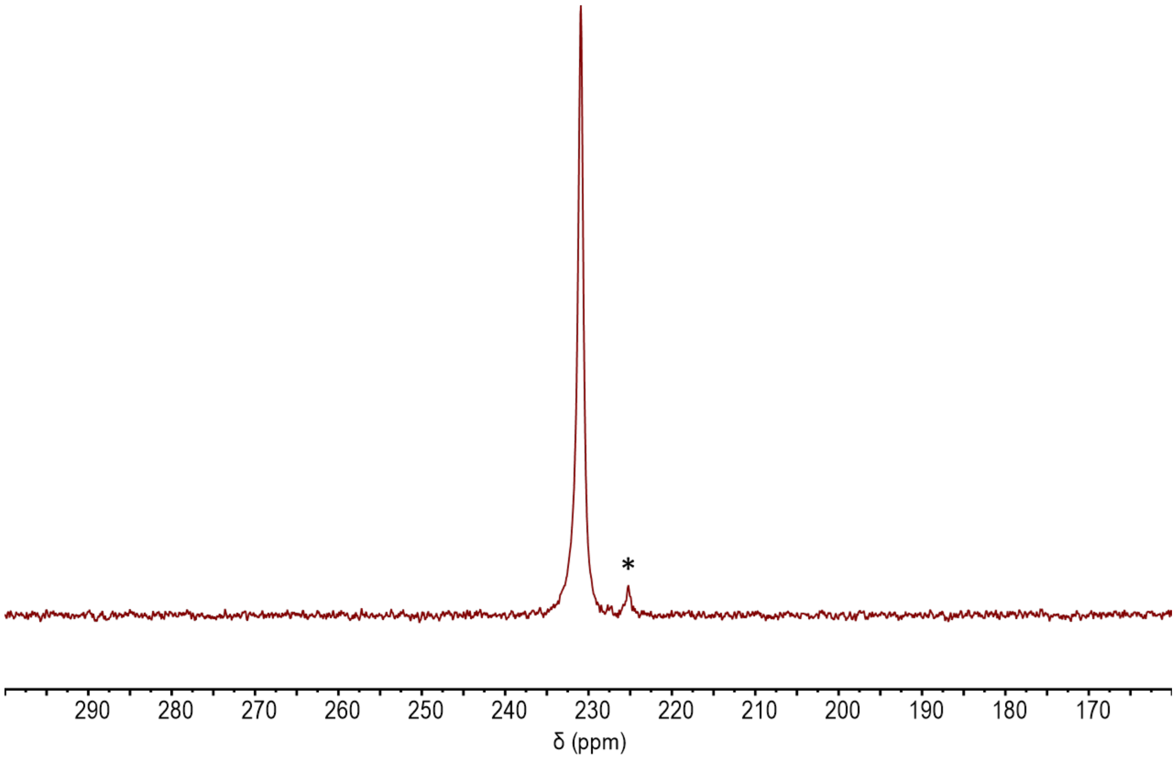

Figure S7: Solution $^{31}P\{^1H\}$ NMR spectrum of $[Mn(^iPr\text{-}PONOP)(CO)_2(THF)][BAr^F_4]$ (242.95 MHz, CD_2Cl_2, 298 K). * denotes $[Mn(^iPr\text{-}PONOP)(CO)_3][BAr^F_4]$ which forms through CO scavenged decomposition in CD_2Cl_2 solution.

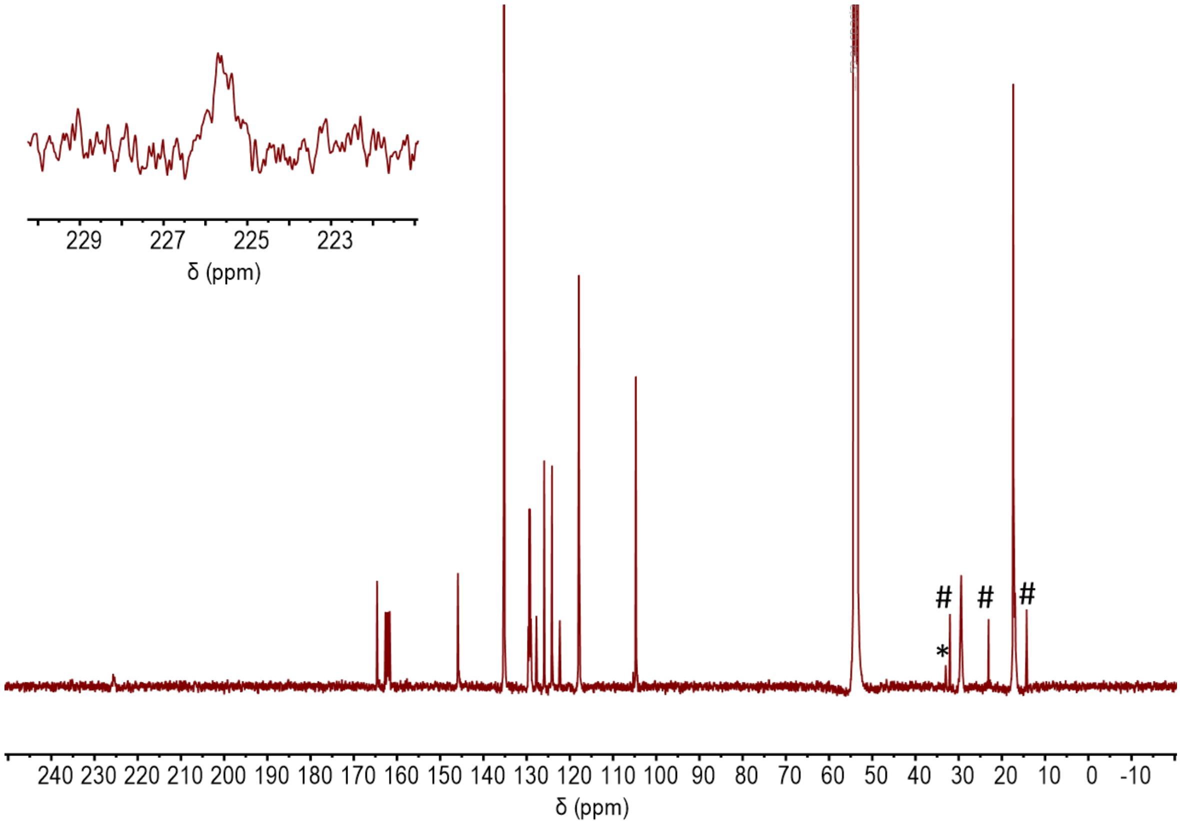

Figure S8: Solution $^{13}C\{^1H\}$ NMR spectrum of $[Mn(^iPr\text{-}PONOP)(CO)_2(THF)][BAr^F_4]$ (150.91 MHz, CD_2Cl_2, 298 K). * denotes $[Mn(^iPr\text{-}PONOP)(CO)_3][BAr^F_4]$ which forms through CO scavenged decomposition in CD_2Cl_2 solution. # denotes co-crystallised hexane.

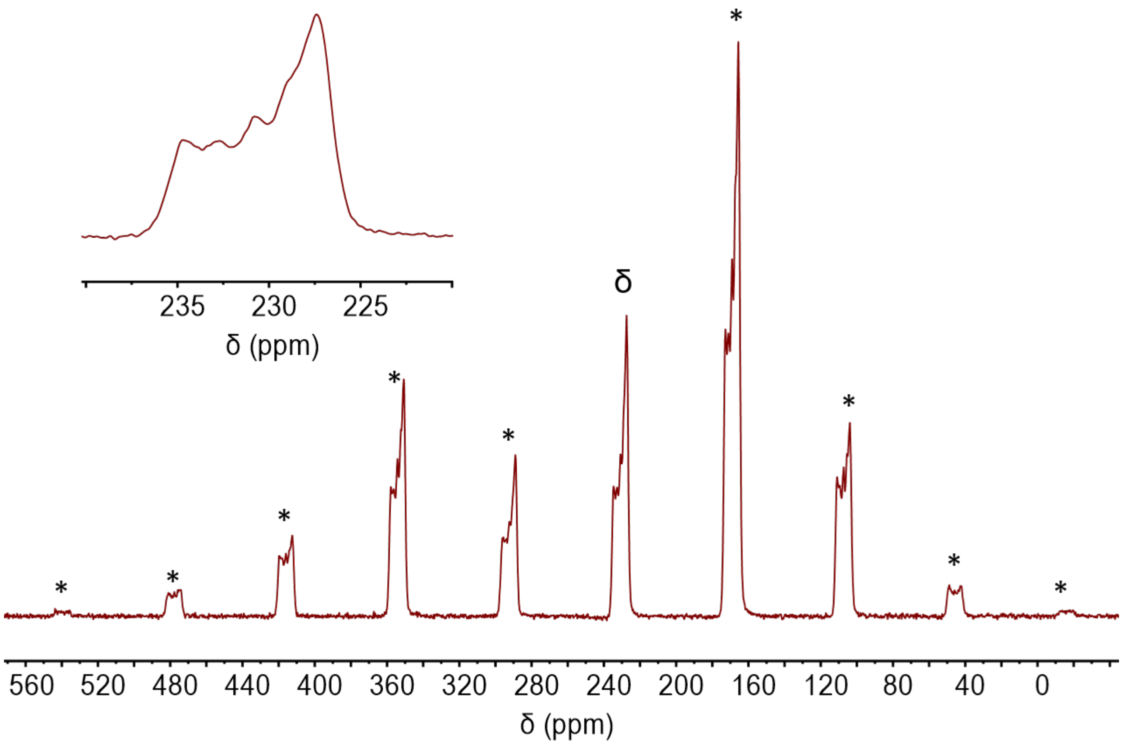

Figure S9: Solid-state $^{31}P\{^{1}H\}$ CP MAS spectrum of [Mn(^{i}Pr-PONOP)(CO)$_2$(THF)][BAr$^{F}_{4}$] (162.06 MHz, 10 kHz spin rate, 298 K). δ denotes isotropic chemical shift. Inset depicts fine coupling of isotropic chemical shift. * denotes spinning side bands.

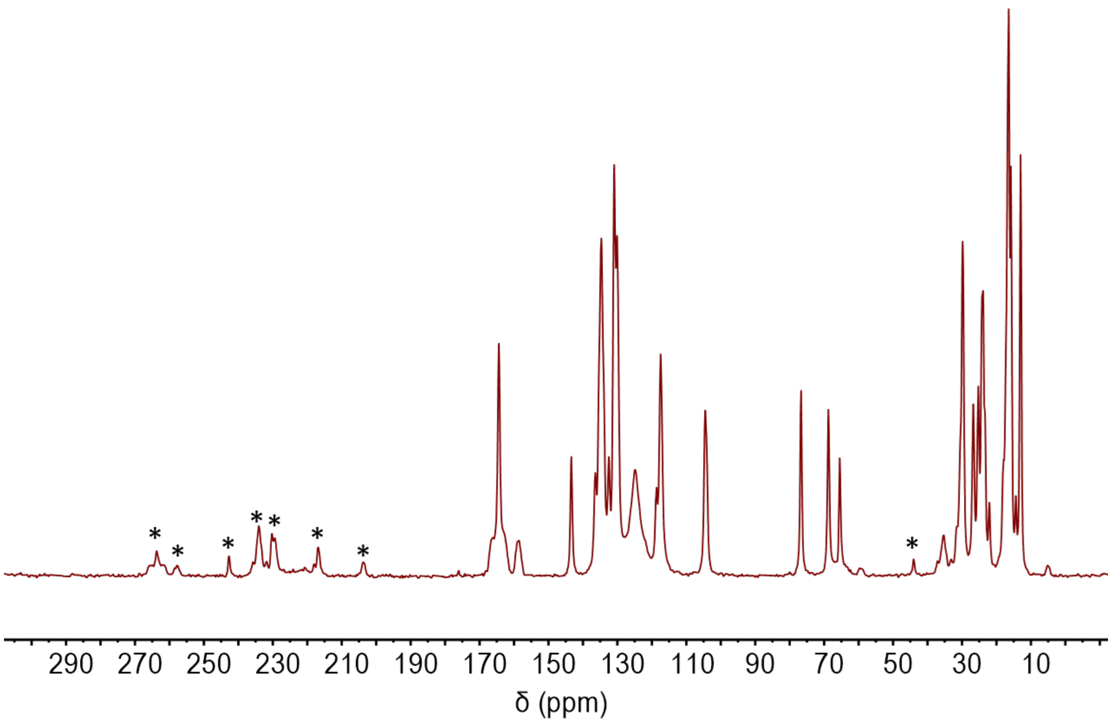

Figure S10: Solid-state $^{13}C\{^{1}H\}$ CP MAS spectrum of [Mn(^{i}Pr-PONOP)(CO)$_2$(THF)][BAr$^{F}_{4}$] (100.66 MHz, 10 kHz spin rate, 298 K). * denotes spinning side bands.

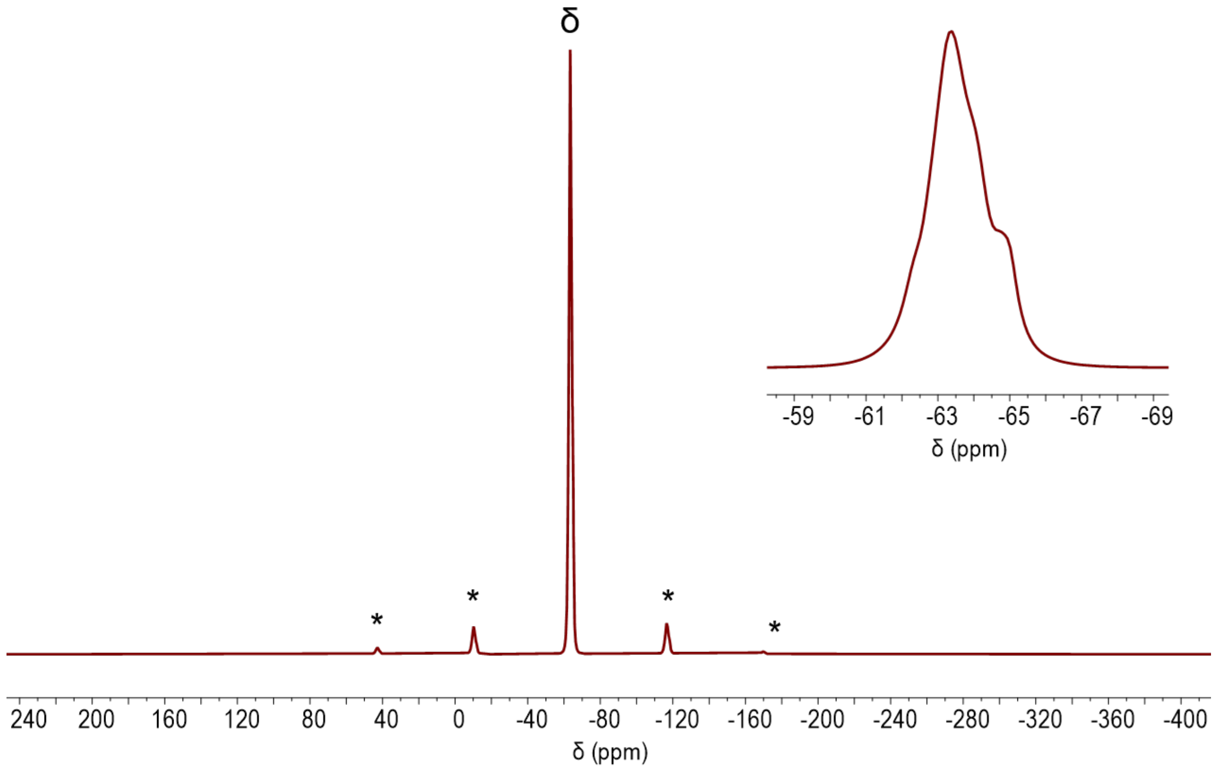

Figure S11: Solid-state $^{19}F\{^{1}H\}$ HPDEC MAS NMR spectrum of $[Mn(^{i}Pr\text{-}PONOP)(THF)(CO)_2][BAr^{F}{}_4]$ (376.5 MHz, 20 kHz spin rate, 298 K). Inset depicts isotropic chemical shift. δ denotes isotropic chemical shift, * denotes spinning side bands.

[(^{i}Pr-PONOP)Mn(CO)$_3$][BAr$^F{}_4$] (solid-state synthesis)

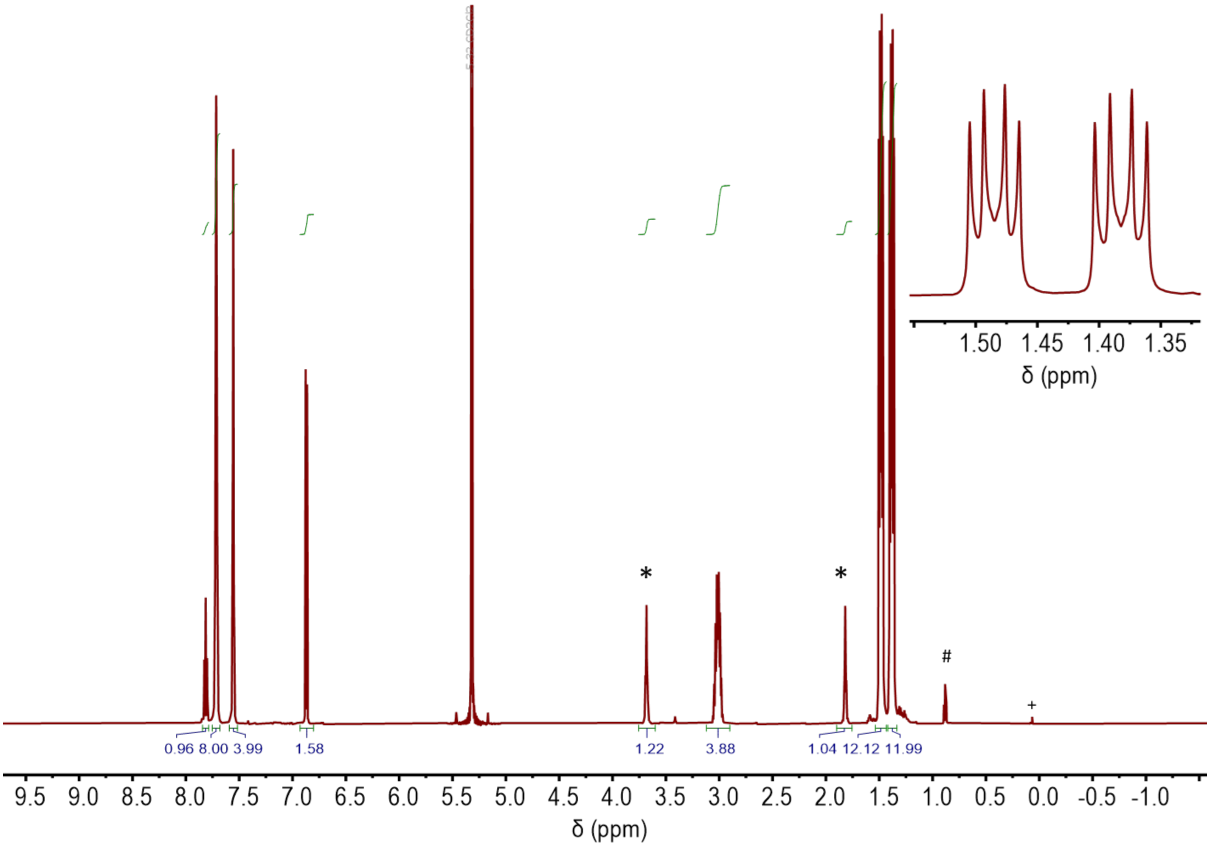

Figure S12: Solution ^{1}H NMR spectrum of [Mn(^{i}Pr-PONOP)(CO)$_3$][BAr$^F{}_4$] synthesised from addition of CO to [Mn(^{i}Pr-PONOP)(THF)(CO)$_2$][BAr$^F{}_4$] (600 MHz, CD_2Cl_2, 298 K). Inset depicts ^{i}Pr methyl environments. * denotes liberated THF, # denotes hexane, + denotes vacuum grease.

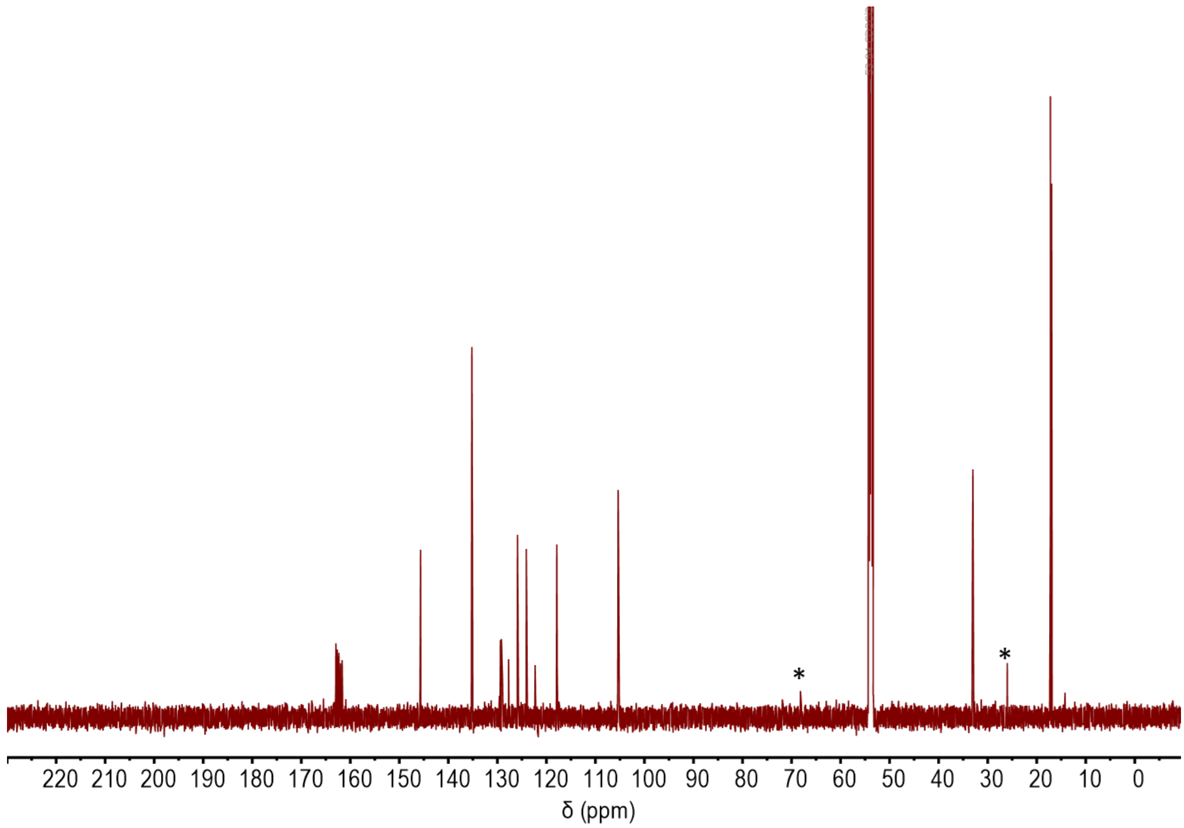

Figure S13: Solution $^{13}C\{^1H\}$ NMR spectrum of [Mn(^{i}Pr-PONOP)(CO)$_3$][BAr$^F{}_4$] synthesised from addition of CO to [Mn(^{i}Pr-PONOP)(THF)(CO)$_2$][BAr$^F{}_4$] (150.91 MHz, CD_2Cl_2, 298 K). * denotes liberated THF.

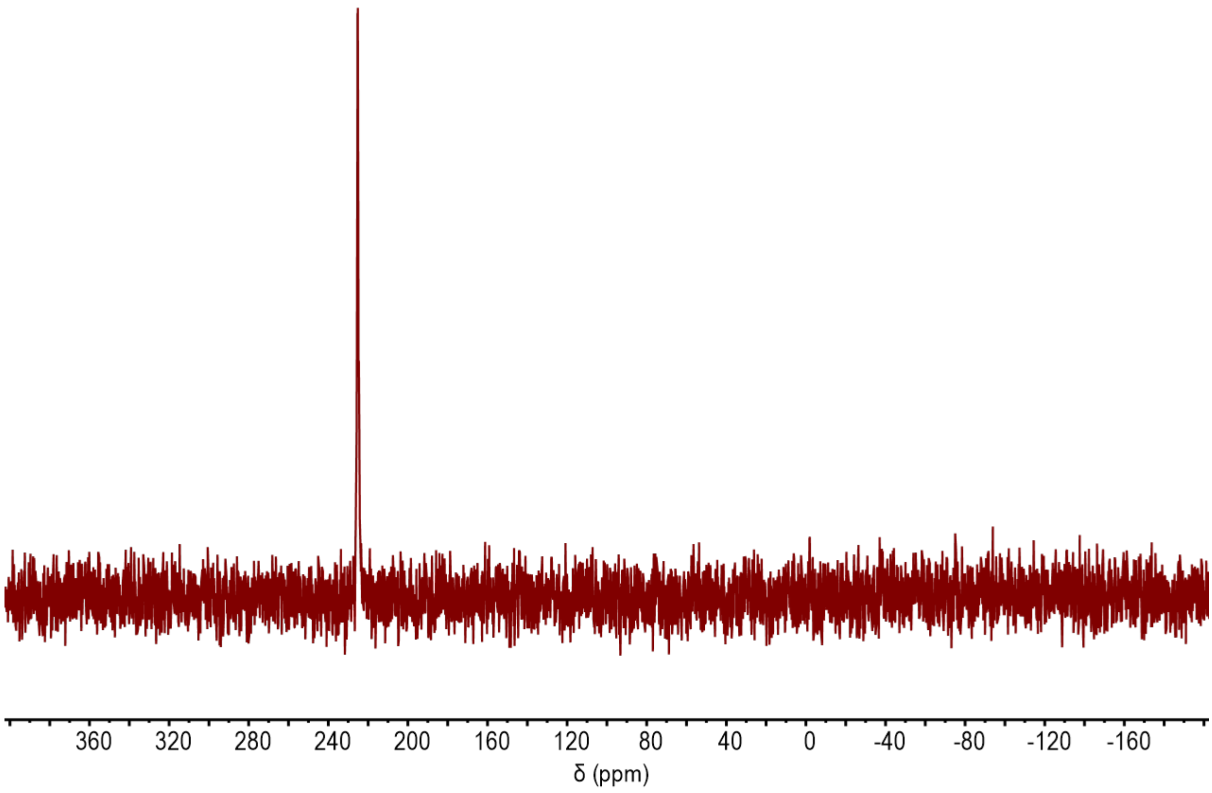

Figure S14: Solution $^{31}P\{^1H\}$ NMR spectrum of [Mn(^{i}Pr-PONOP)(CO)$_3$][BAr$^F{}_4$] synthesised from addition of CO to [Mn(^{i}Pr-PONOP)(THF)(CO)$_2$][BAr$^F{}_4$] (242.95 MHz, CD_2Cl_2, 298 K).

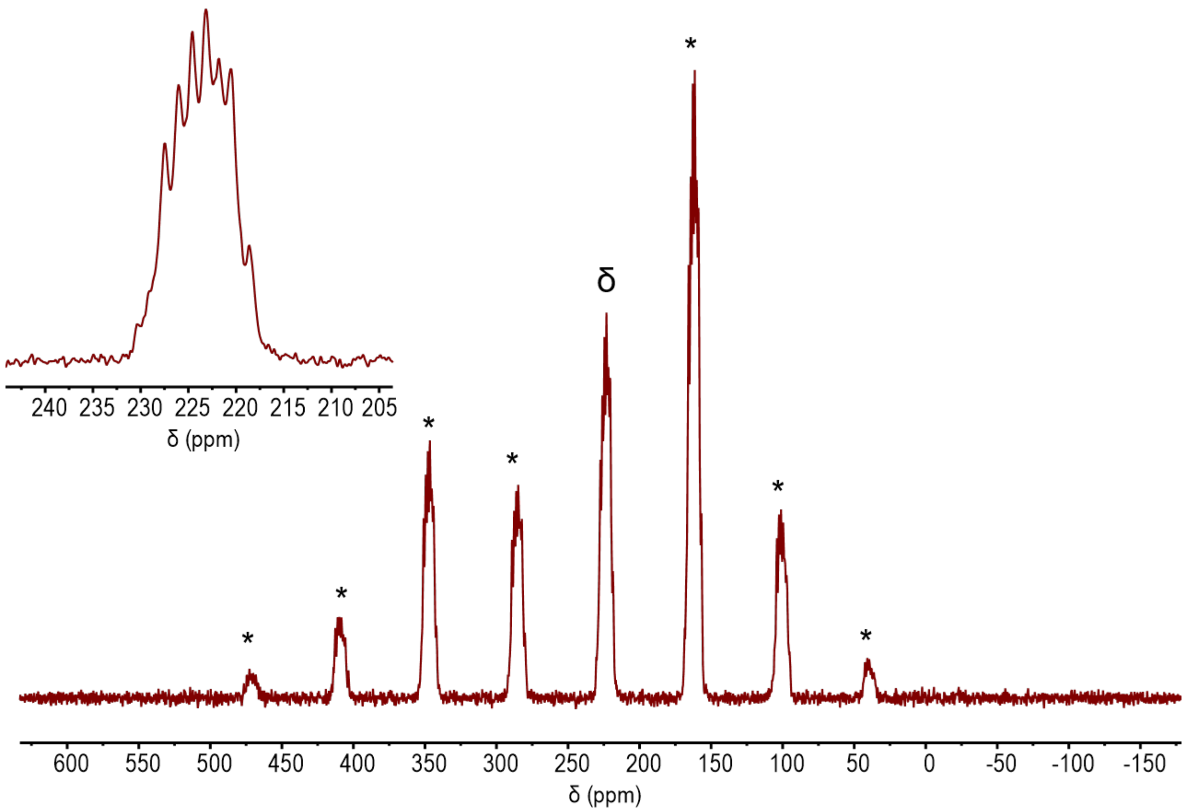

Figure S15: Solid-state $^{31}P\{^{1}H\}$ NMR spectrum of [Mn(^{i}Pr-PONOP)(CO)$_3$][BAr$^{F}{}_4$] synthesised from addition of CO to [Mn(^{i}Pr-PONOP)(THF)(CO)$_2$][BAr$^{F}{}_4$] (162.06 MHz, 10 kHz spin rate, 298 K). δ denotes isotropic chemical shift, * denotes spinning side bands.

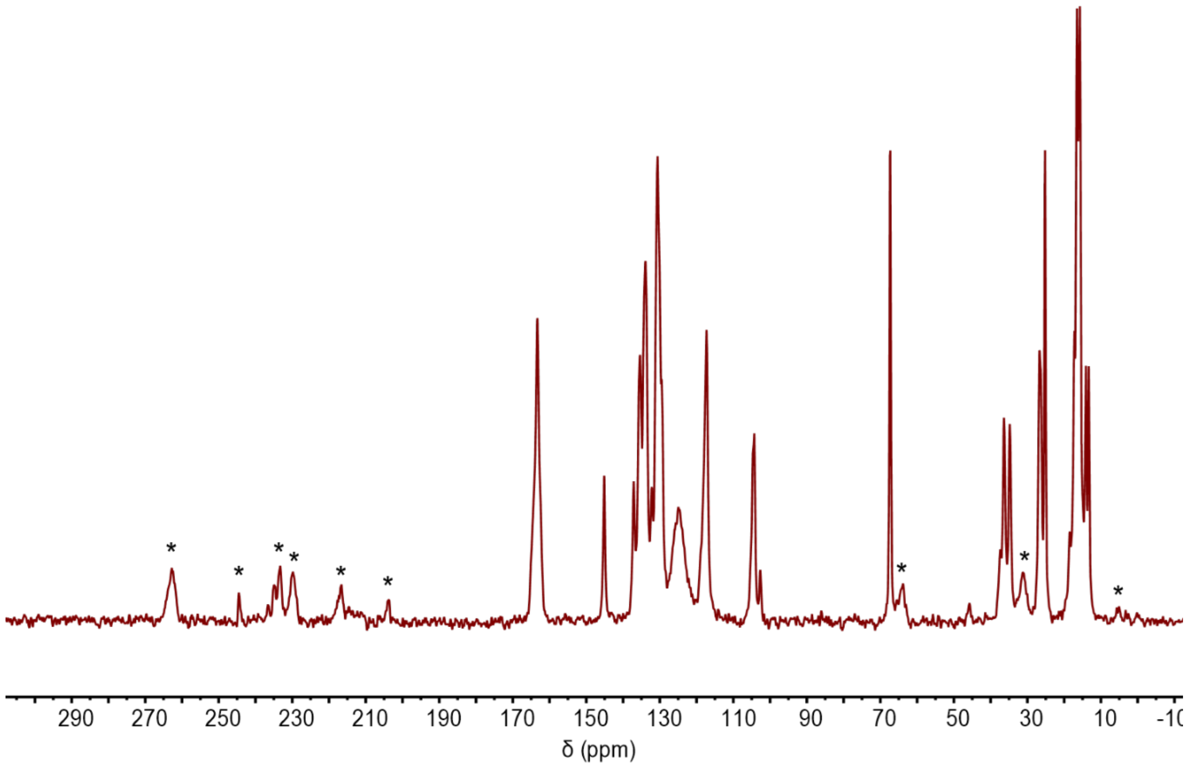

Figure S16: Solid-state $^{13}C\{^{1}H\}$ NMR spectrum of [Mn(^{i}Pr-PONOP)(CO)$_3$][BAr$^{F}{}_4$] synthesised from addition of CO to [Mn(^{i}Pr-PONOP)(THF)(CO)$_2$][BAr$^{F}{}_4$] (100.66 MHz, 10 kHz spin rate, 298 K). * denotes spinning side bands.

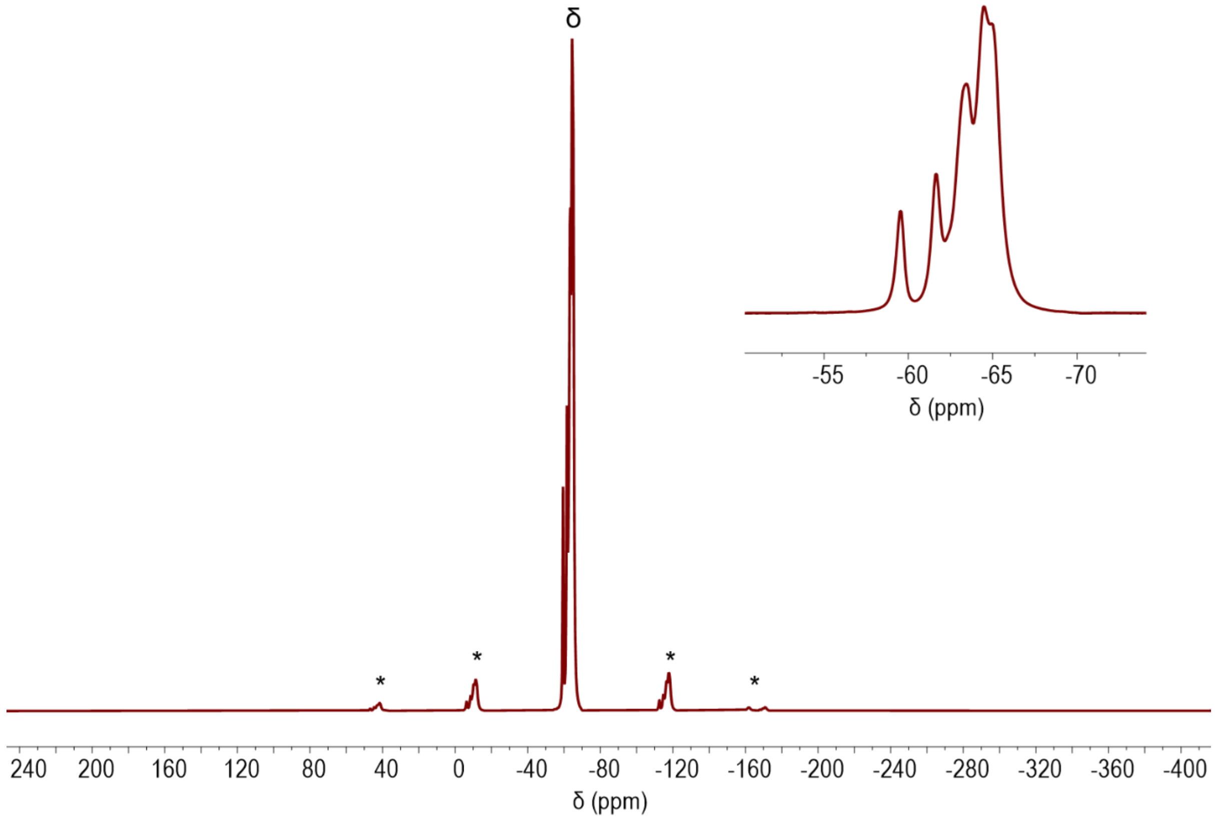

Figure S17: Solid-state $^{19}F\{^{1}H\}$ HPDEC MAS NMR spectrum of [Mn(^{i}Pr-PONOP)$(CO)_3$][BAr^F_4] (376.5 MHz, 20 kHz spin rate, 298 K). Inset depicts isotropic chemical shift. δ denotes isotropic chemical shift, * denotes spinning side bands.

[Re(^{t}Bu-PONOP)Br(CO)$_2$]

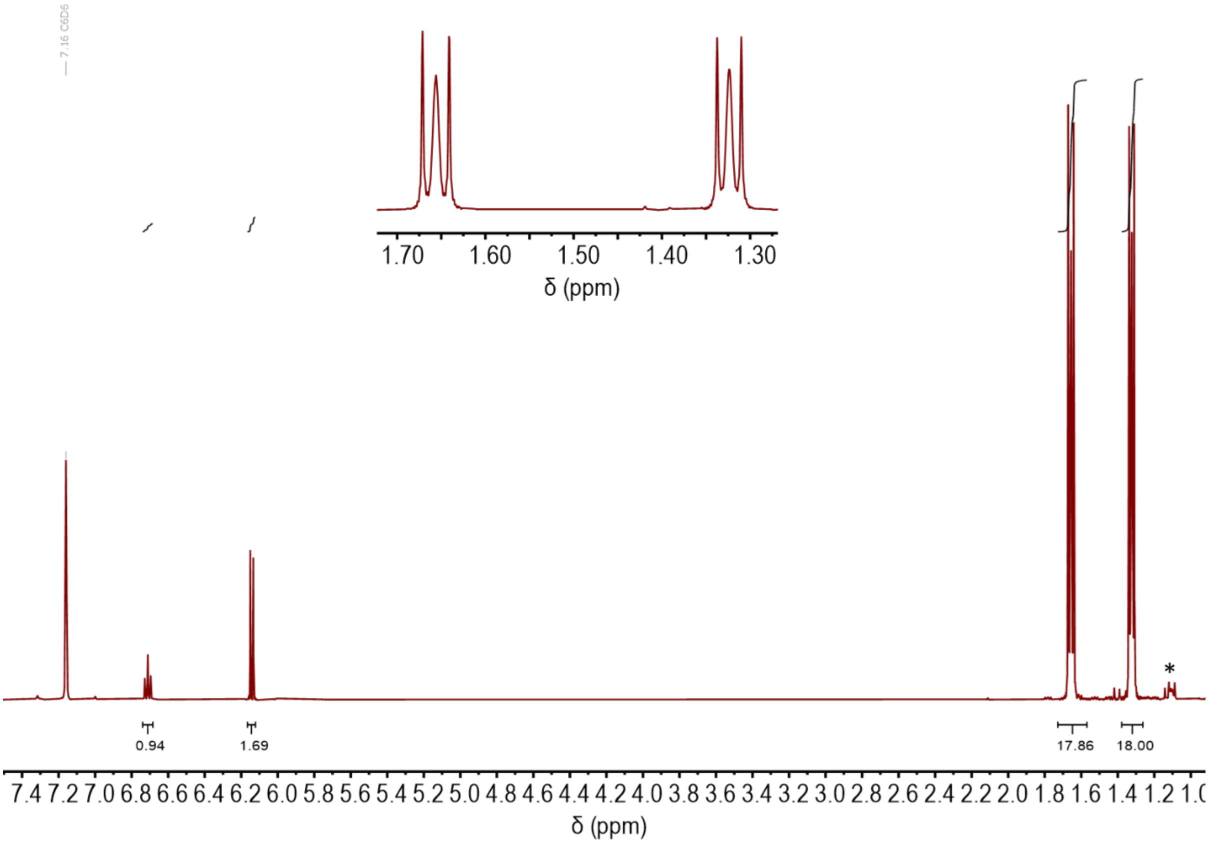

Figure S18: Solution ^{1}H NMR spectrum of [Re(^{t}Bu-PONOP)Br(CO)$_2$] (500 MHz, C_6D_6, 298 K). Inset depicts virtual triplet coupling of ^{t}Bu methyl groups. * denotes unknown minor impurity.

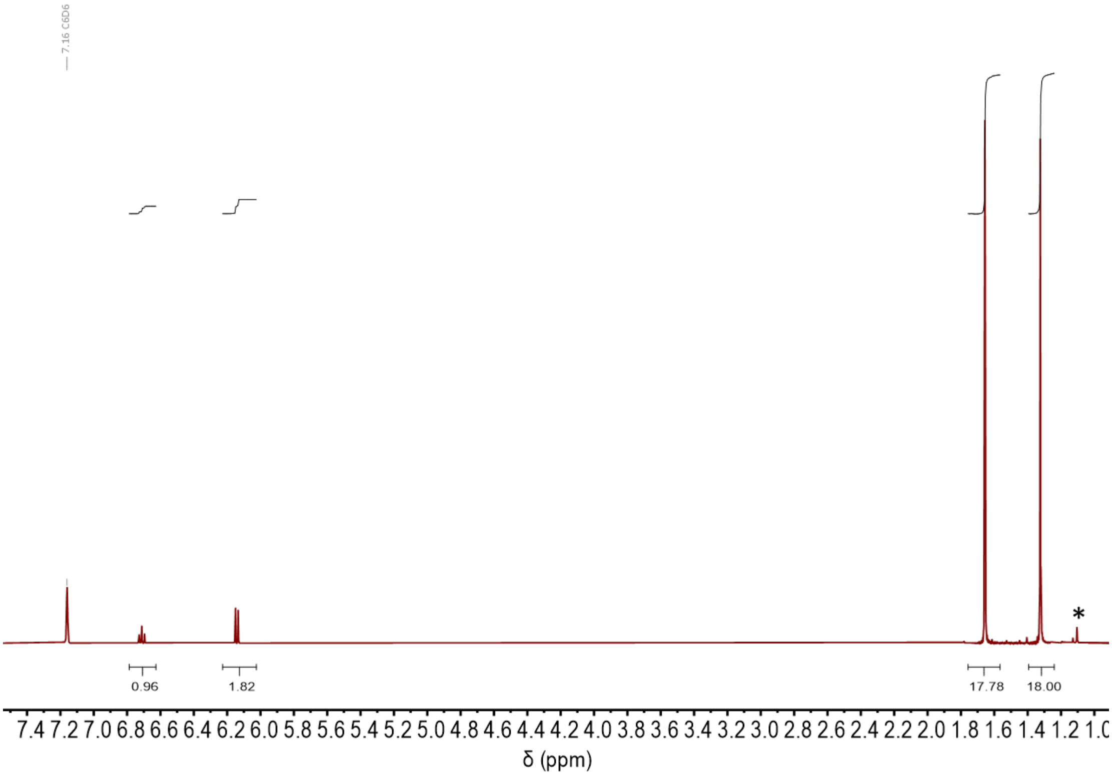

Figure S19: Solution ^{1}H{^{31}P} NMR spectrum of [Re(^{t}Bu-PONOP)Br(CO)$_2$] (500 MHz, C_6D_6, 298 K). * denotes unknown minor impurity.

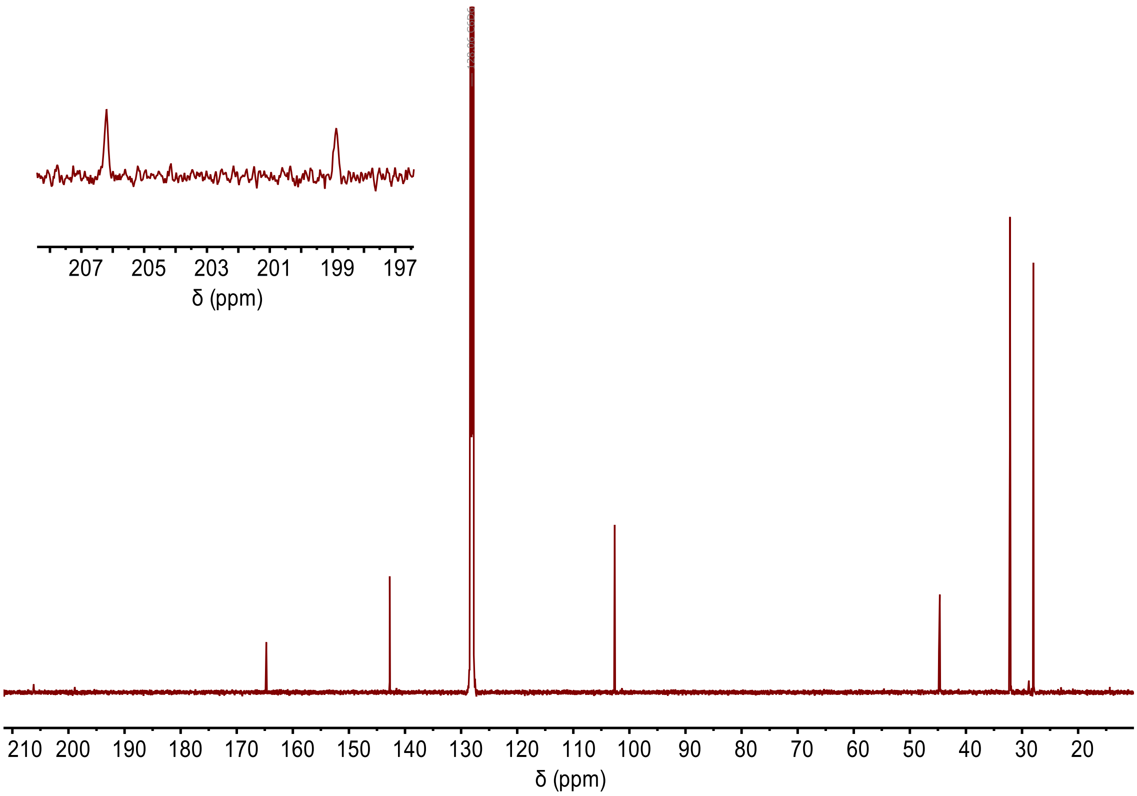

Figure S20: Solution $^{13}C\{^1H\}$ NMR spectrum of [Re(^{t}Bu-PONOP)Br(CO)$_2$] (125.8 MHz, C_6D_6, 298 K). Inset depicts CO resonances.

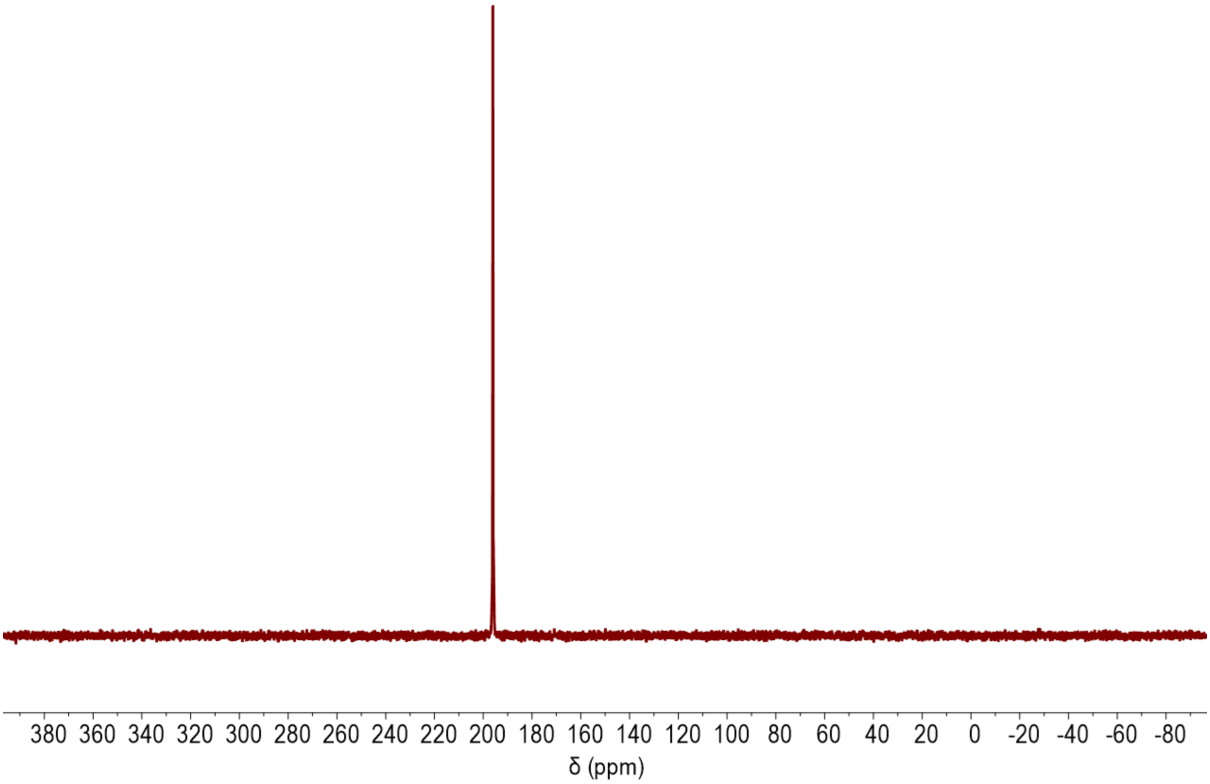

Figure S21: Solution $^{31}P\{^1H\}$ NMR spectrum of [Re(^{t}Bu-PONOP)Br(CO)$_2$] (202.52 MHz, C_6D_6, 298 K).

[Re(^{t}Bu-PONOP)(CO)$_2$][BAr$^F{}_4$]

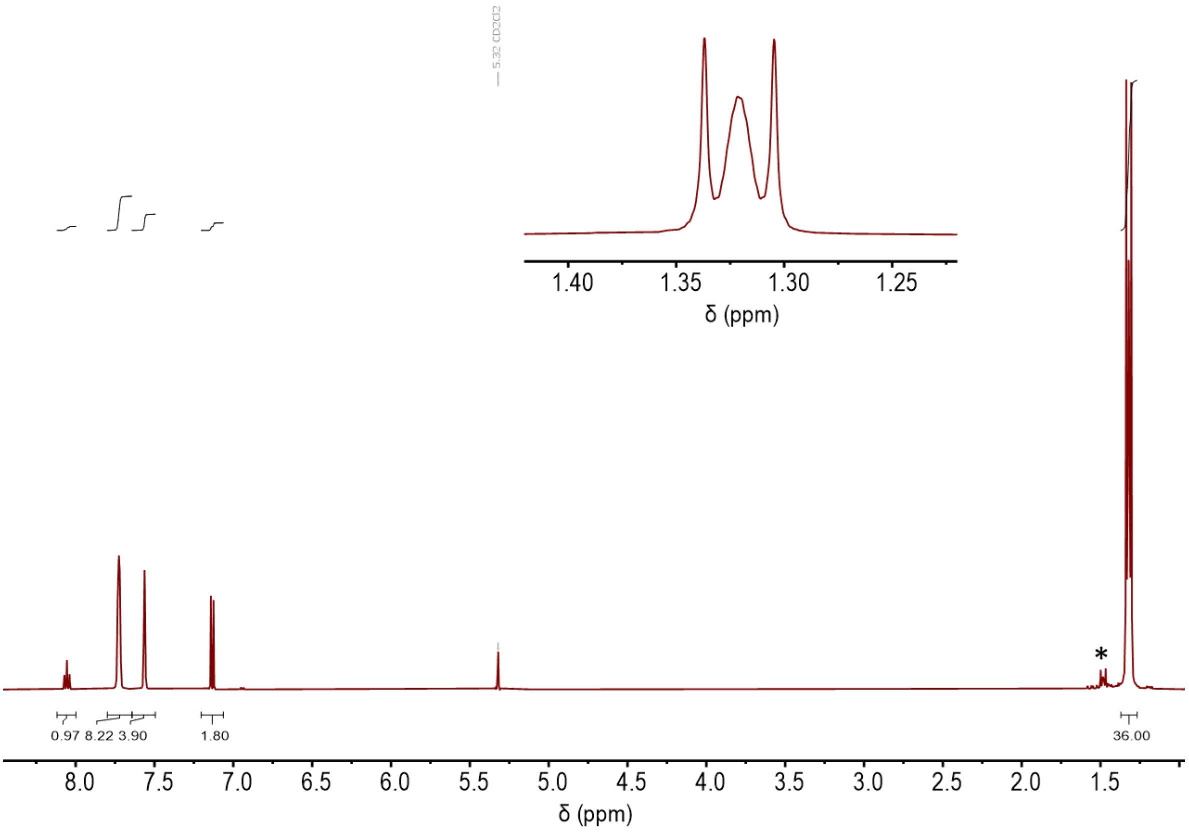

Figure S22: Solution ^{1}H NMR spectrum of [Re(^{t}Bu-PONOP)(CO)$_2$][BAr$^F{}_4$] (500 MHz, CD_2Cl_2, 298 K). Inset depicts virtual triplet coupling of ^{t}Bu methyl groups. * denotes [Re(^{t}Bu-PONOP)(CO)$_3$][BAr$^F{}_4$] which is formed through slow CO scavenged decomposition in CD_2Cl_2.

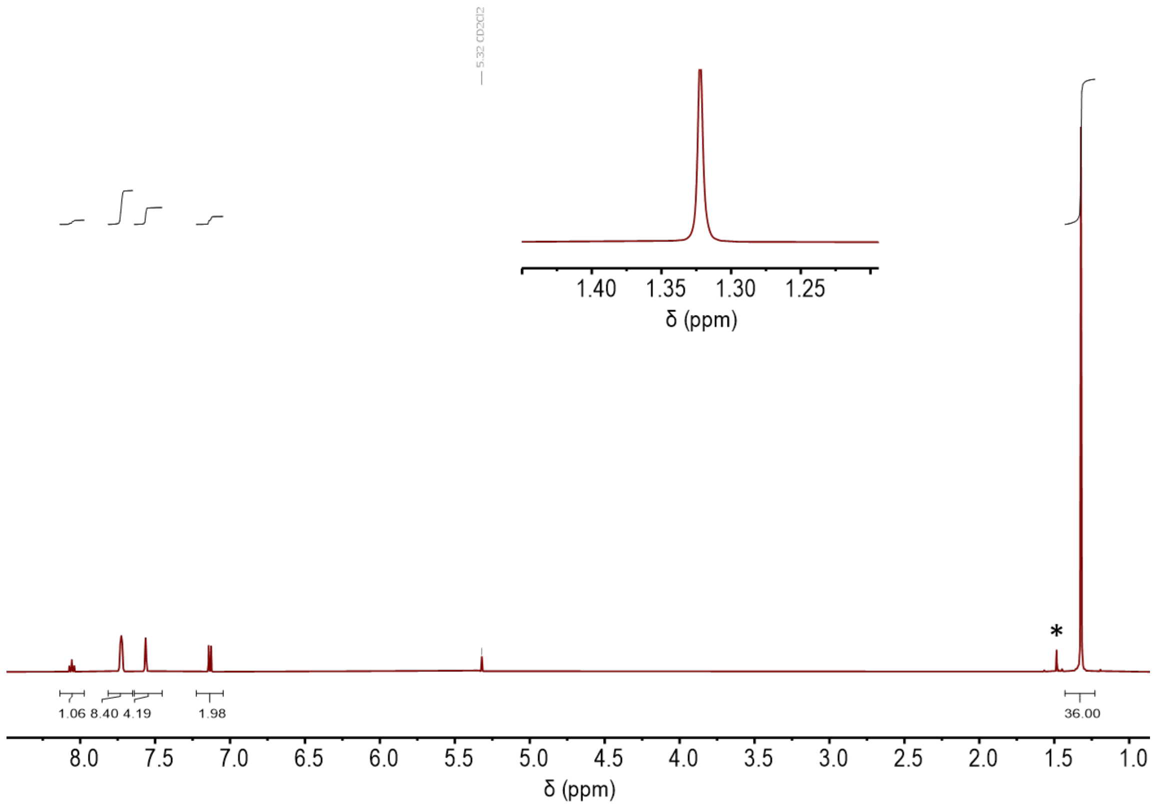

Figure S23: Solution $^{1}H\{^{31}P\}$ NMR spectrum of [Re(^{t}Bu-PONOP)(CO)$_2$][BAr$^{F}{}_4$] (500 MHz, CD_2Cl_2, 298 K). Inset depicts virtual triplet coupling of ^{t}Bu methyl groups collapses to a singlet upon ^{31}P decoupling. * denotes [Re(^{t}Bu-PONOP)(CO)$_3$][BAr$^{F}{}_4$] which is formed through slow CO scavenged decomposition in CD_2Cl_2.

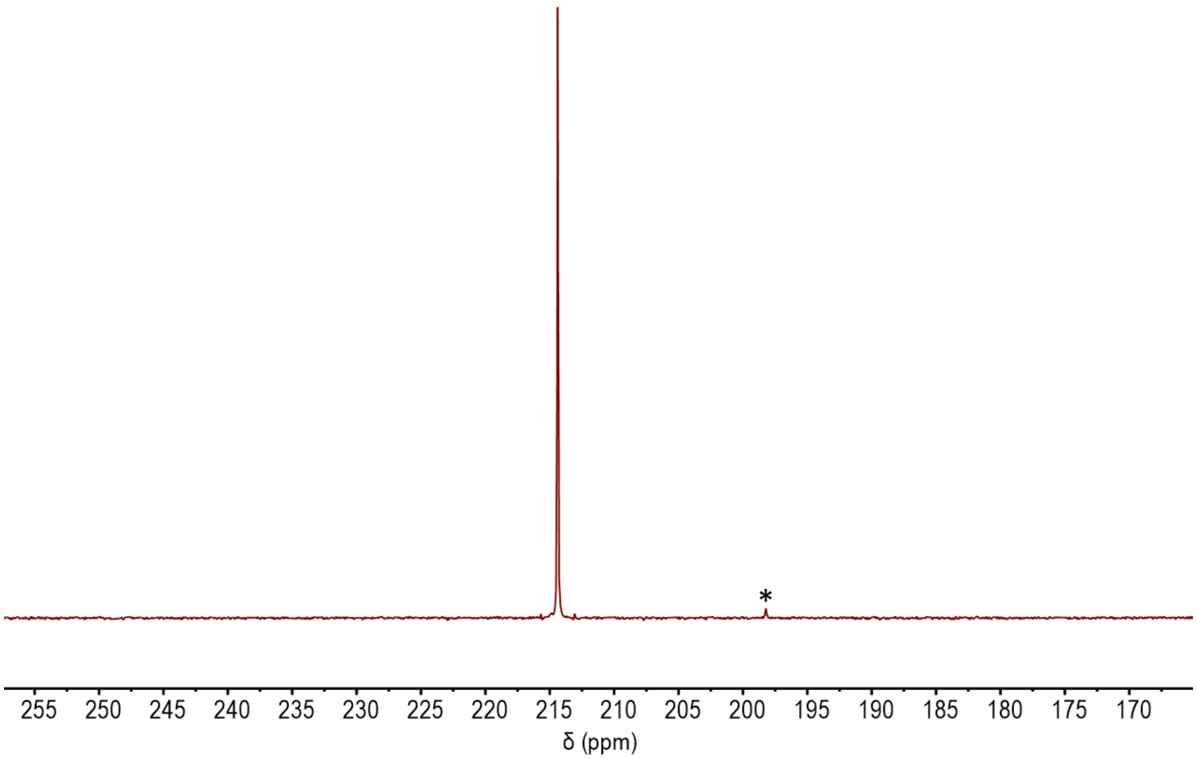

Figure S24: Solution $^{31}P\{^{1}H\}$ NMR spectrum of [Re(^{t}Bu-PONOP)(CO)$_2$][BAr$^{F}{}_4$] (202.53 MHz, CD_2Cl_2, 298 K). * denotes [Re(^{t}Bu-PONOP)(CO)$_3$][BAr$^{F}{}_4$] which is formed through slow CO scavenged decomposition in CD_2Cl_2.

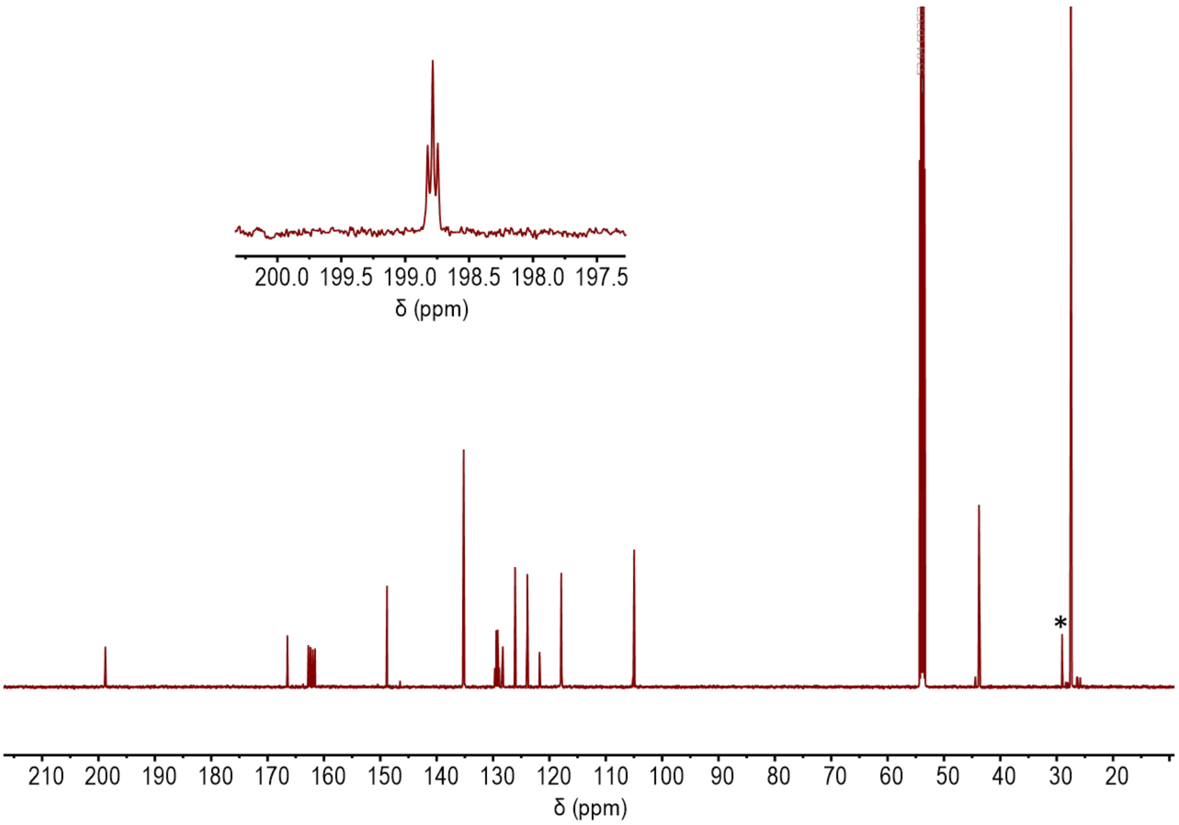

Figure S25: Solution $^{31}P\{^{1}H\}$ NMR spectrum of [Re(^{t}Bu-PONOP)$(CO)_2$][BAr^{F}_4] (125.80 MHz, CD_2Cl_2, 298 K). Inset depicts CO resonance. * denotes [Re(^{t}Bu-PONOP)$(CO)_3$][BAr^{F}_4] which is formed through slow CO scavenged decomposition in CD_2Cl_2.

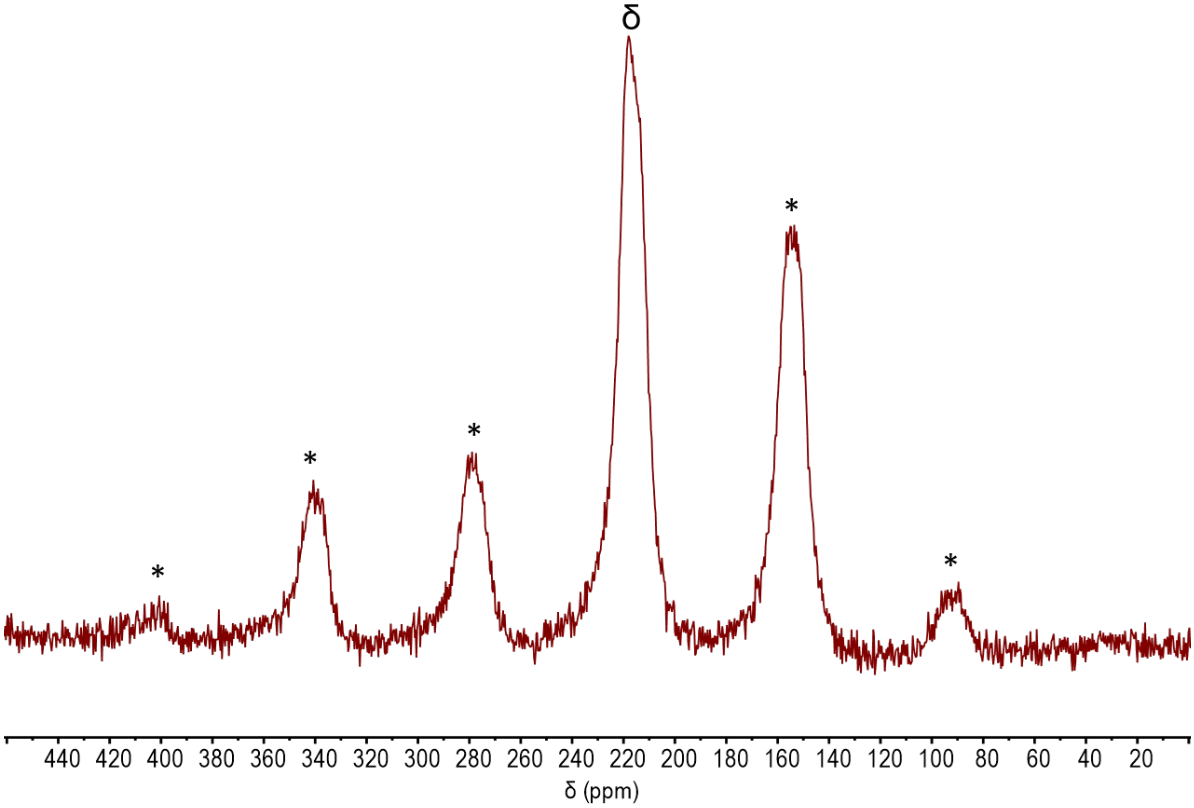

Figure S26: Solid-state $^{31}P\{^{1}H\}$ CP MAS spectrum of [Re(^{t}Bu-PONOP)$(CO)_2$][BAr^{F}_4] (162.02 MHz, 10 kHz spin rate, 298 K). δ denotes isotropic chemical shift. * denotes spinning side bands.

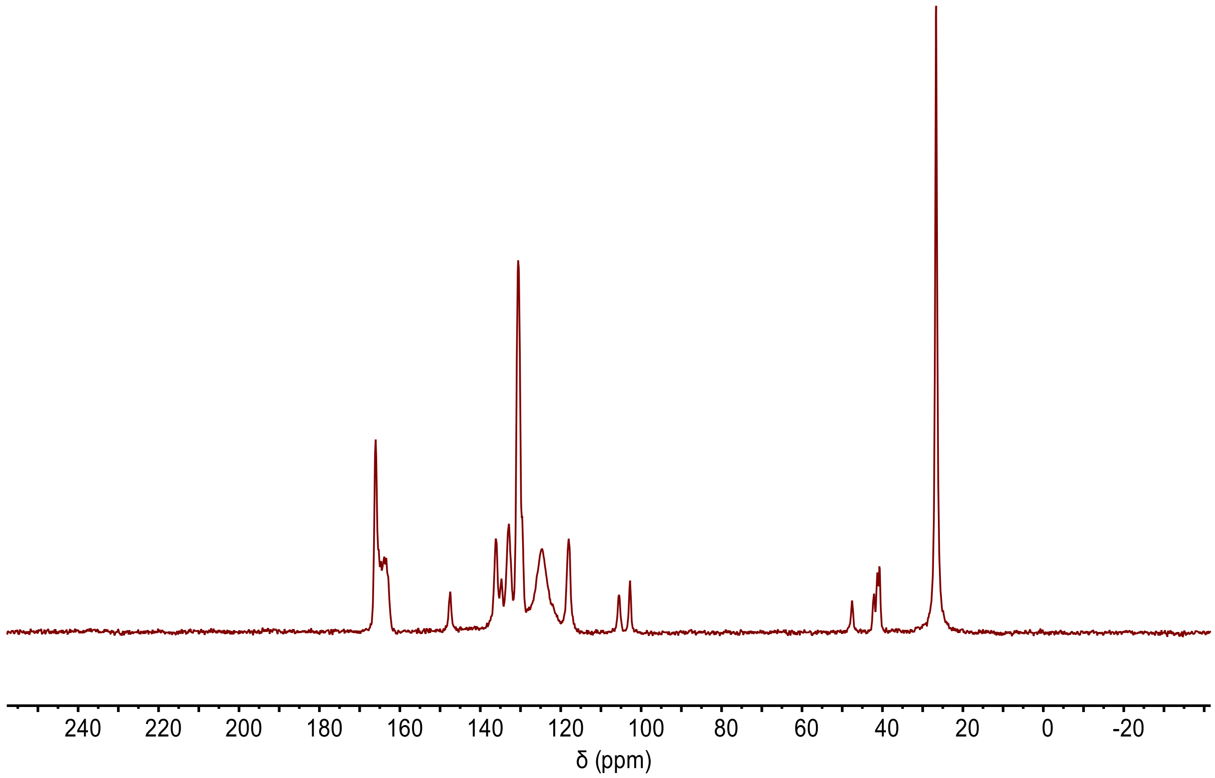

Figure S27: Side-band suppressed solid-state $^{13}C\{^{1}H\}$ CP MAS spectrum of $[Re(^{t}Bu\text{-}PONOP)(CO)_2][BAr^{F}_4]$ (100.63 MHz, 10 kHz spin rate, 298 K).

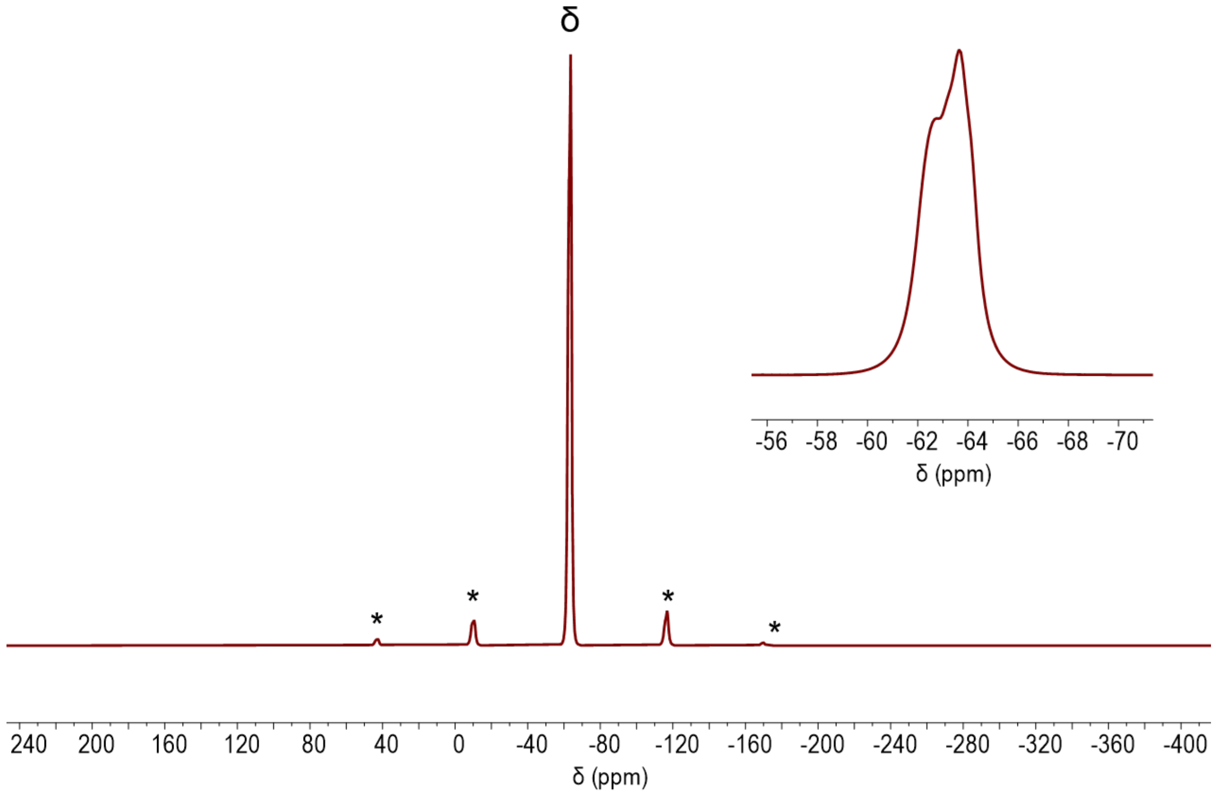

Figure S28: Solid-state $^{19}F\{^{1}H\}$ HPDEC MAS NMR spectrum of $[Re(^{t}Bu\text{-}PONOP)(CO)_2][BAr^{F}_4]$ (376.5 MHz, 20 kHz spin rate, 298 K). Inset and δ depict isotropic chemical shift, * denote spinning side bands.

[Re(^{t}Bu-PONOP)(CO)$_3$][BAr$^F{}_4$] (solid-state)

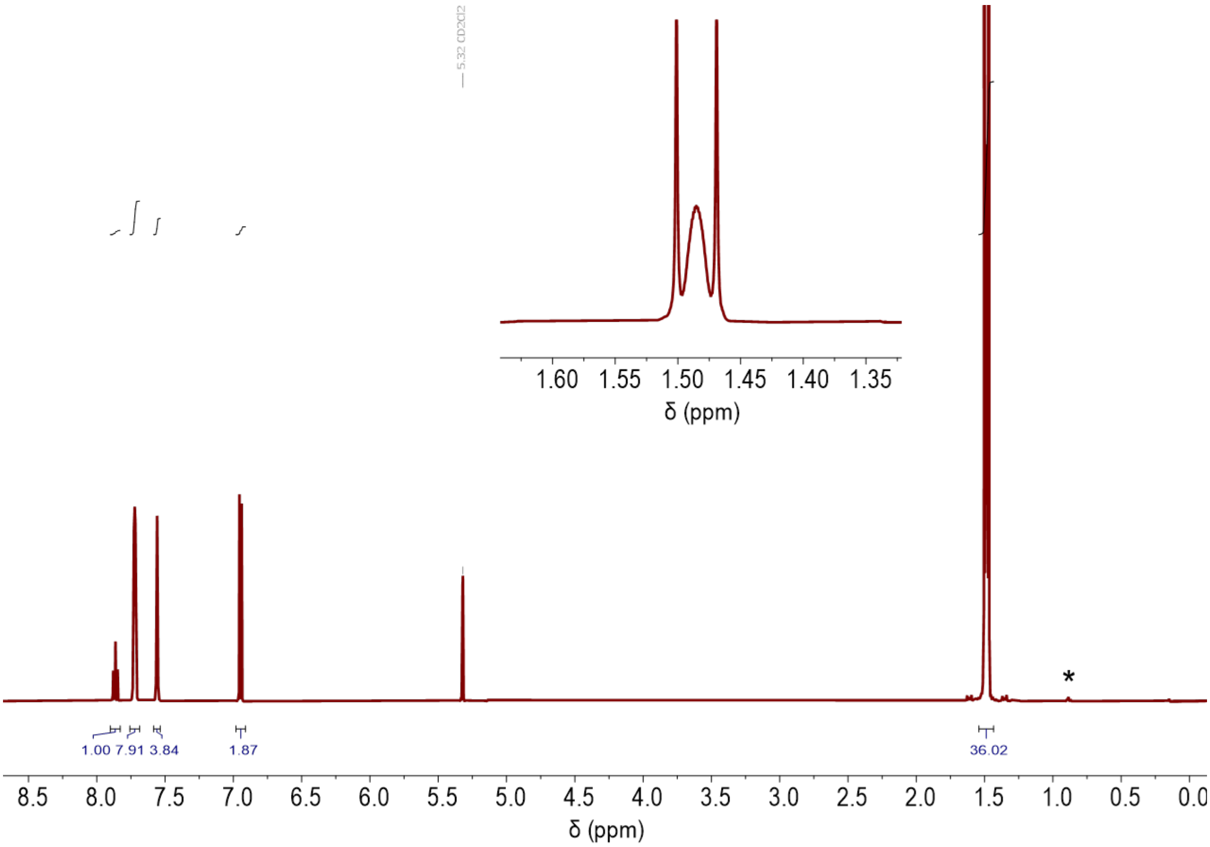

Figure S29: Solution $^1H\{^{31}P\}$ NMR spectrum of [Re(^{t}Bu-PONOP)(CO)$_3$][BAr$^F{}_4$] (500 MHz, CD_2Cl_2, 298 K). Inset depicts virtual triplet coupling of ^{t}Bu methyl groups. * denotes trace pentane impurity.

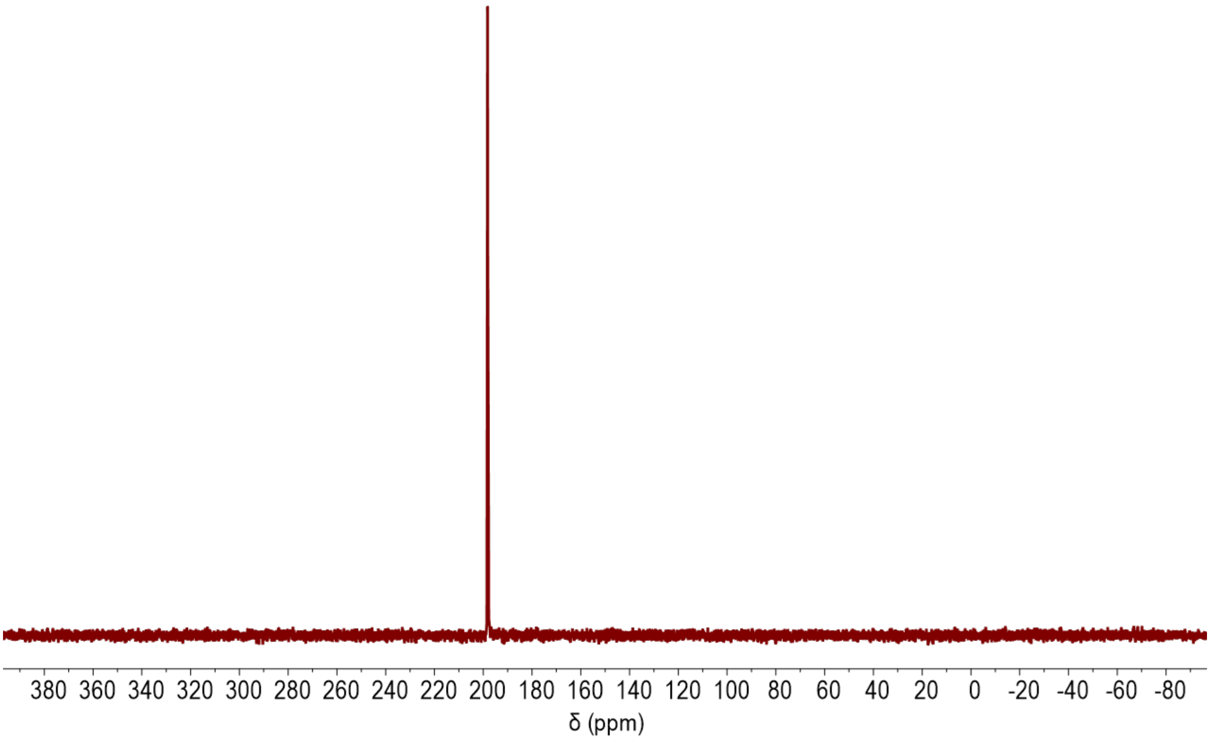

Figure S30: Solution $^{31}P\{^{1}H\}$ NMR Spectrum of $[Re(^{t}Bu\text{-}PONOP)(CO)_3][BAr^F_4]$ (202.52 MHz, CD_2Cl_2, 298 K).

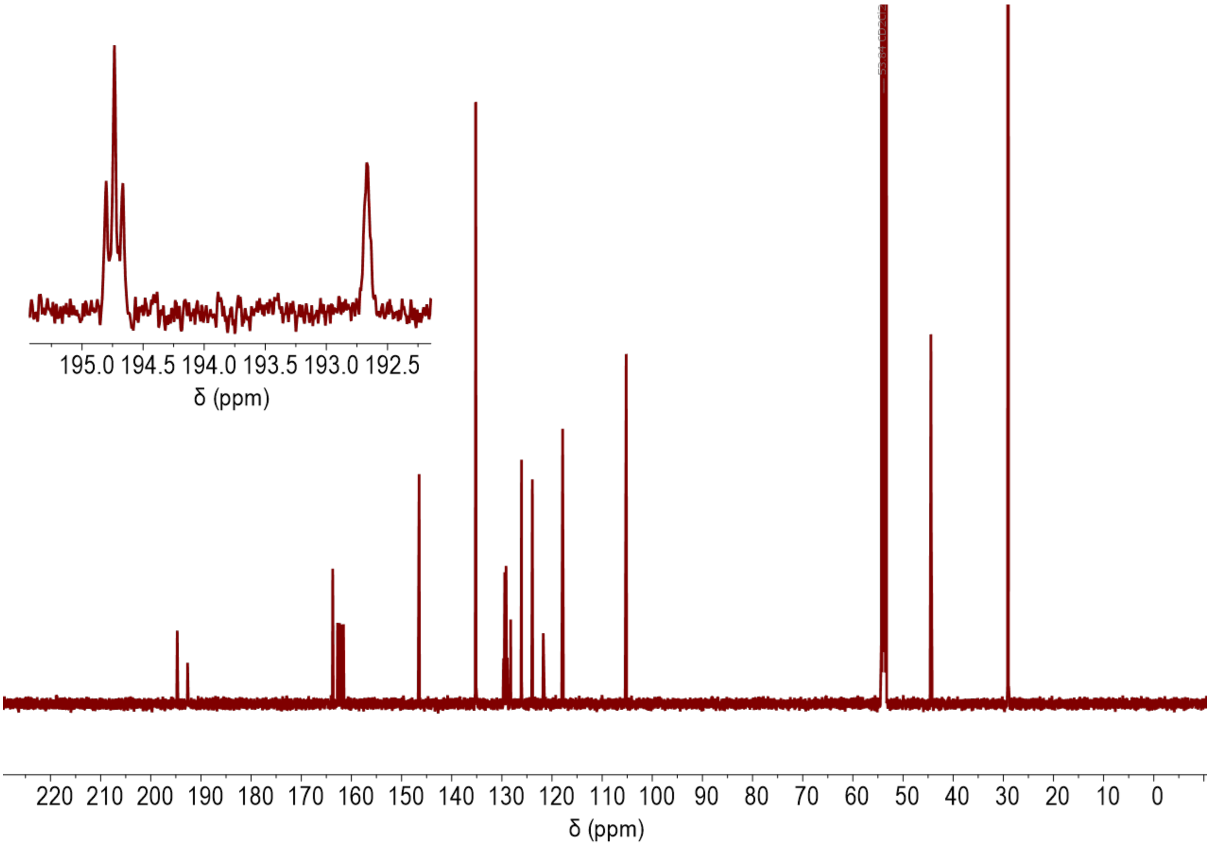

Figure S31: Solution $^{13}C\{^{1}H\}$ NMR Spectrum of $[Re(^{t}Bu\text{-}PONOP)(CO)_3][BAr^F_4]$ (125.8 MHz, CD_2Cl_2, 298 K). Inset depicts carbonyl resonances.

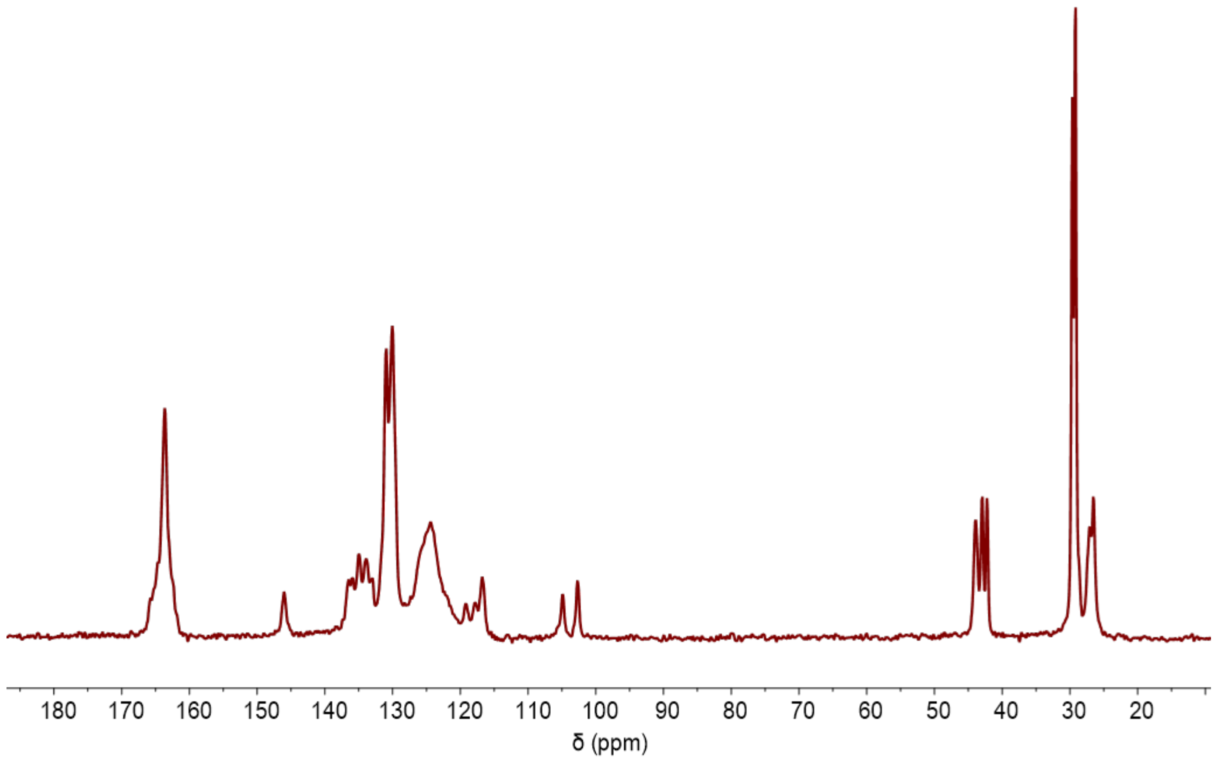

Figure S32: Side band suppressed solid-state $^{13}C\{^{1}H\}$ CP MAS NMR spectrum of $[Re(^{t}Bu\text{-}PONOP)(CO)_3][BAr^F{}_4]$ (100.63 MHz, 10 kHz spin rate, 298 K).

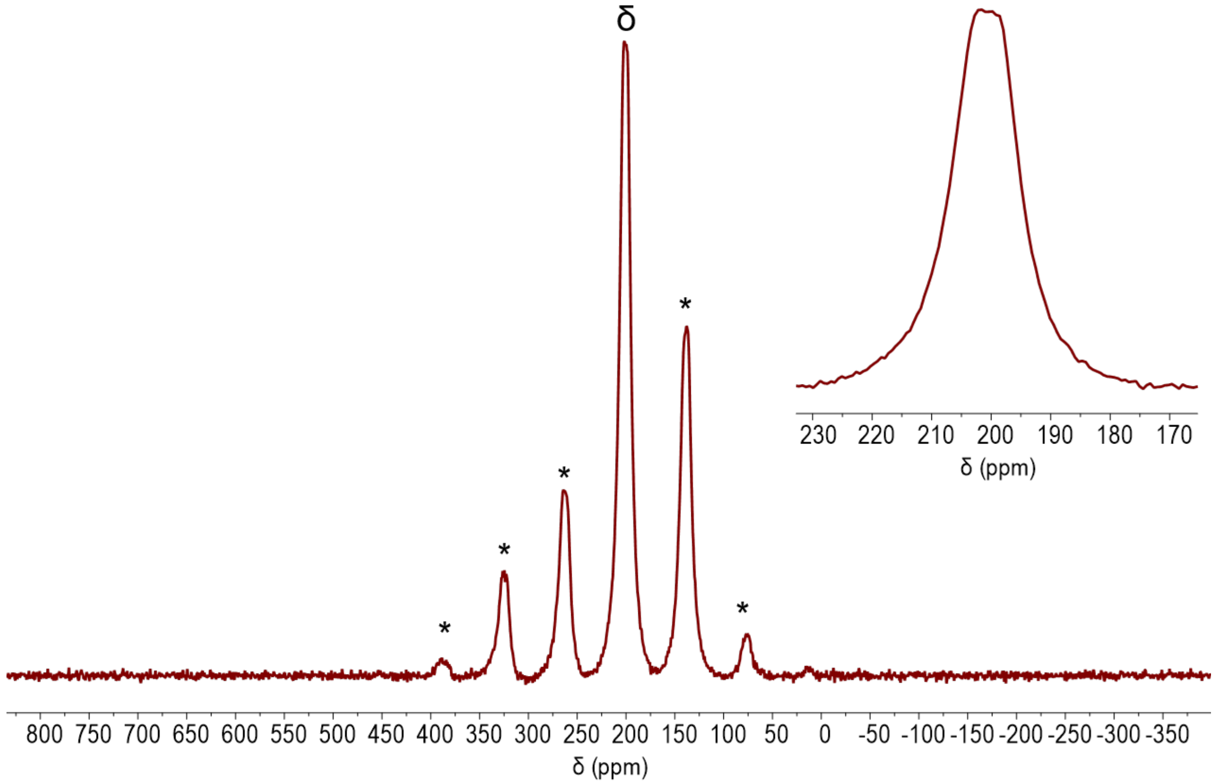

Figure S33: Solid-state $^{31}P\{^{1}H\}$ CP MAS NMR spectrum of [Re(^{t}Bu-PONOP)$(CO)_3$][BAr^{F}_4] (162.03 MHz, 10 kHz spin rate, 298 K). Inset and δ denote isotropic chemical shift, * denotes spinning side bands.

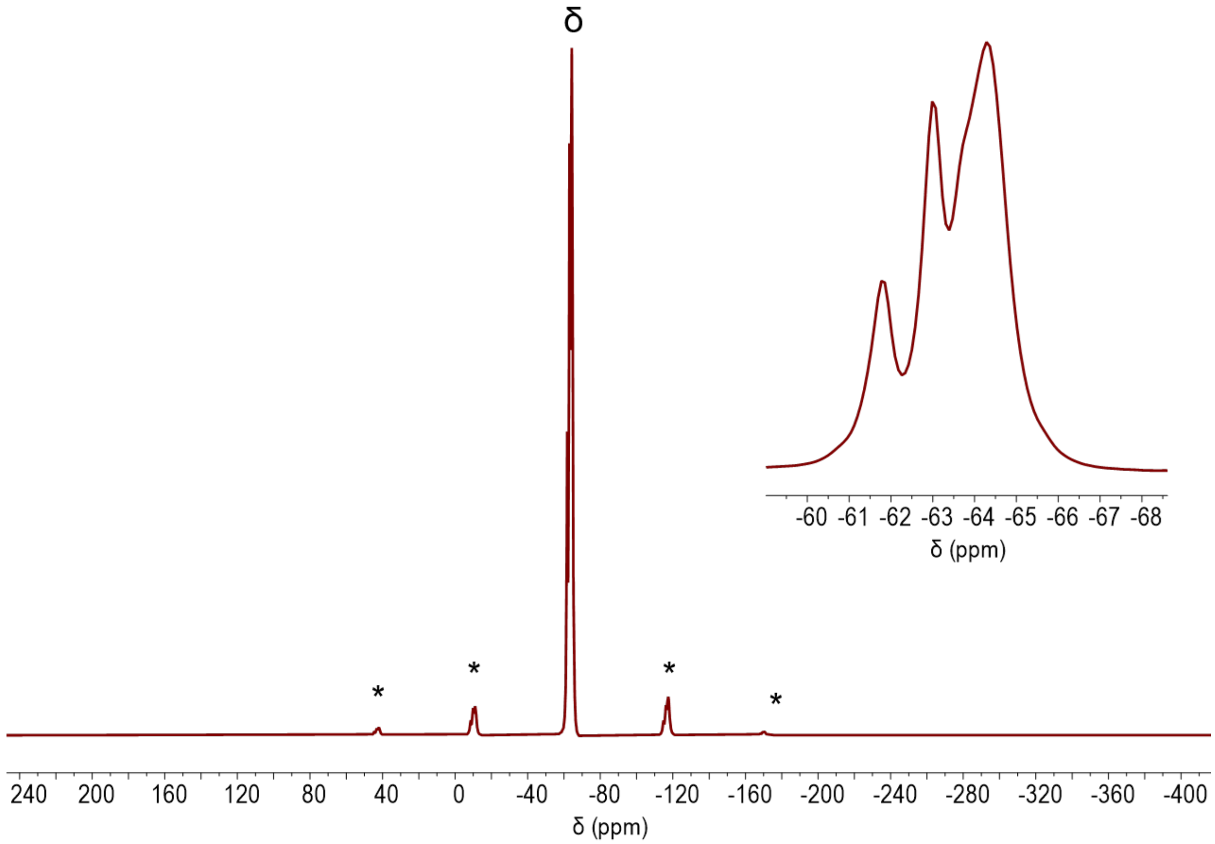

Figure S34: Solid-state $^{19}F\{^{1}H\}$ HPDEC MAS NMR spectrum of [Re(^{t}Bu-PONOP)$(CO)_3$][BAr^{F}_4] (376.5 MHz, 20 kHz spin rate, 298K). Inset and δ denote isotropic chemical shifts, * denotes spinning side bands.

S1d. IR Spectra

[Mn(^{i}Pr-PONOP)(CO)$_3$][BAr$^F{}_4$]

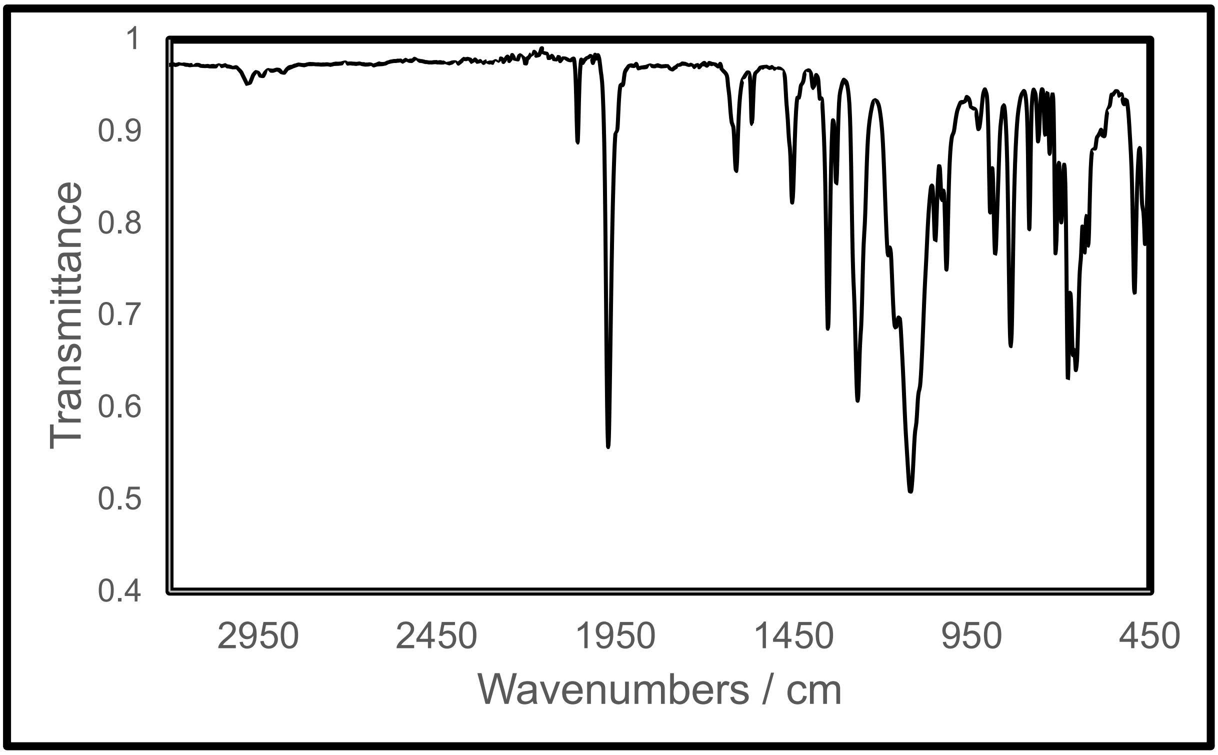

Figure S35: ATR IR spectrum of [Mn(^{i}Pr-PONOP)(CO)$_3$][BAr$^F{}_4$].

[Mn(^{i}Pr-PONOP)(THF)(CO)$_2$][BAr$^F{}_4$]

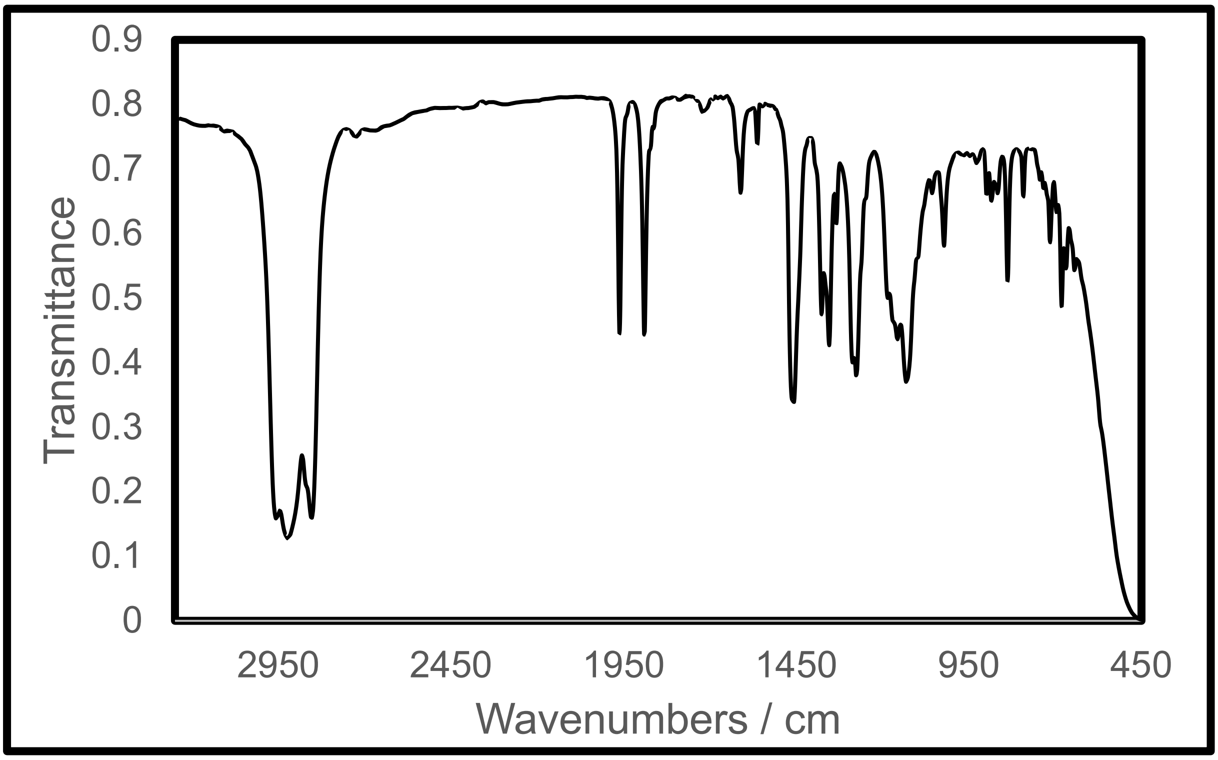

Figure S36: IR Spectrum of [Mn(^{i}Pr-PONOP)(THF)(CO)$_2$][BAr$^F{}_4$] in nujol.

[Mn(^{i}Pr-PONOP)(CO)$_3$][BAr$^F{}_4$] (solid-state synthesis)

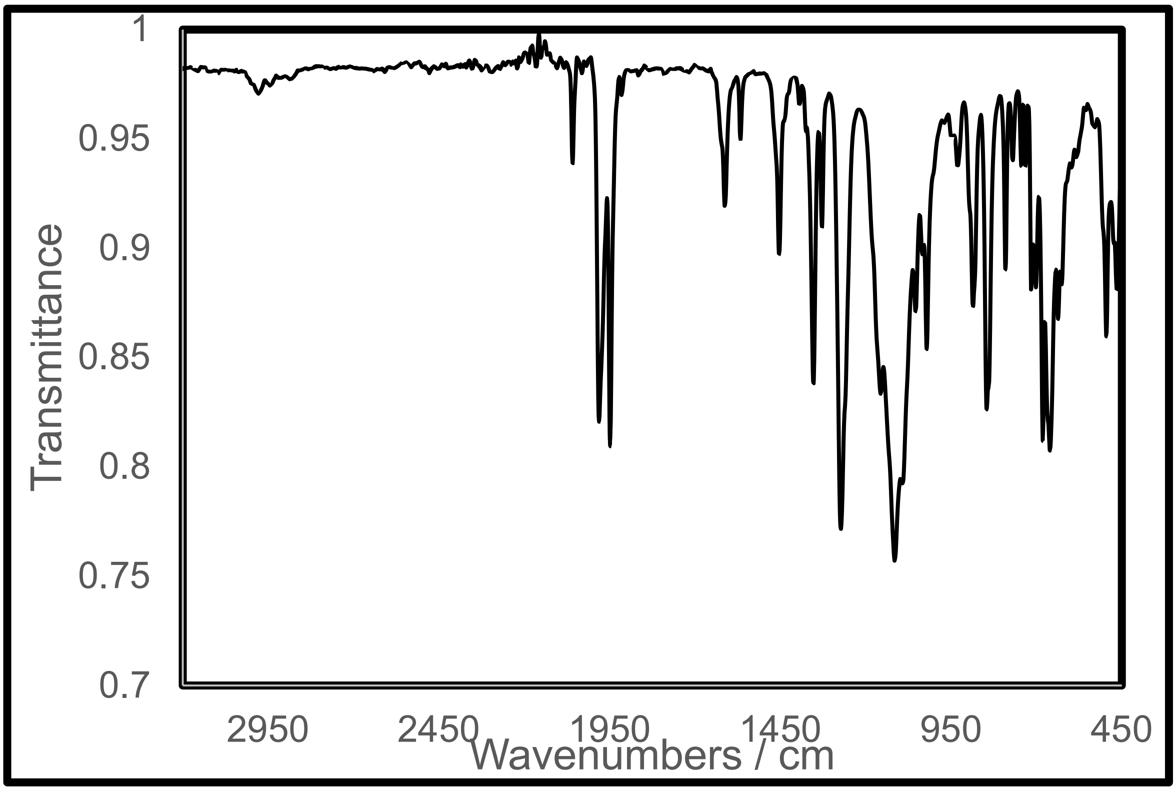

Figure S37: ATR IR spectrum of [Mn(^{i}Pr-PONOP)(CO)$_3$][BAr$^F{}_4$] formed from addition of CO to [Mn(^{i}Pr-PONOP)(THF)(CO)$_2$][BAr$^F{}_4$].

[Re(^{t}Bu-PONOP)Br(CO)$_2$]

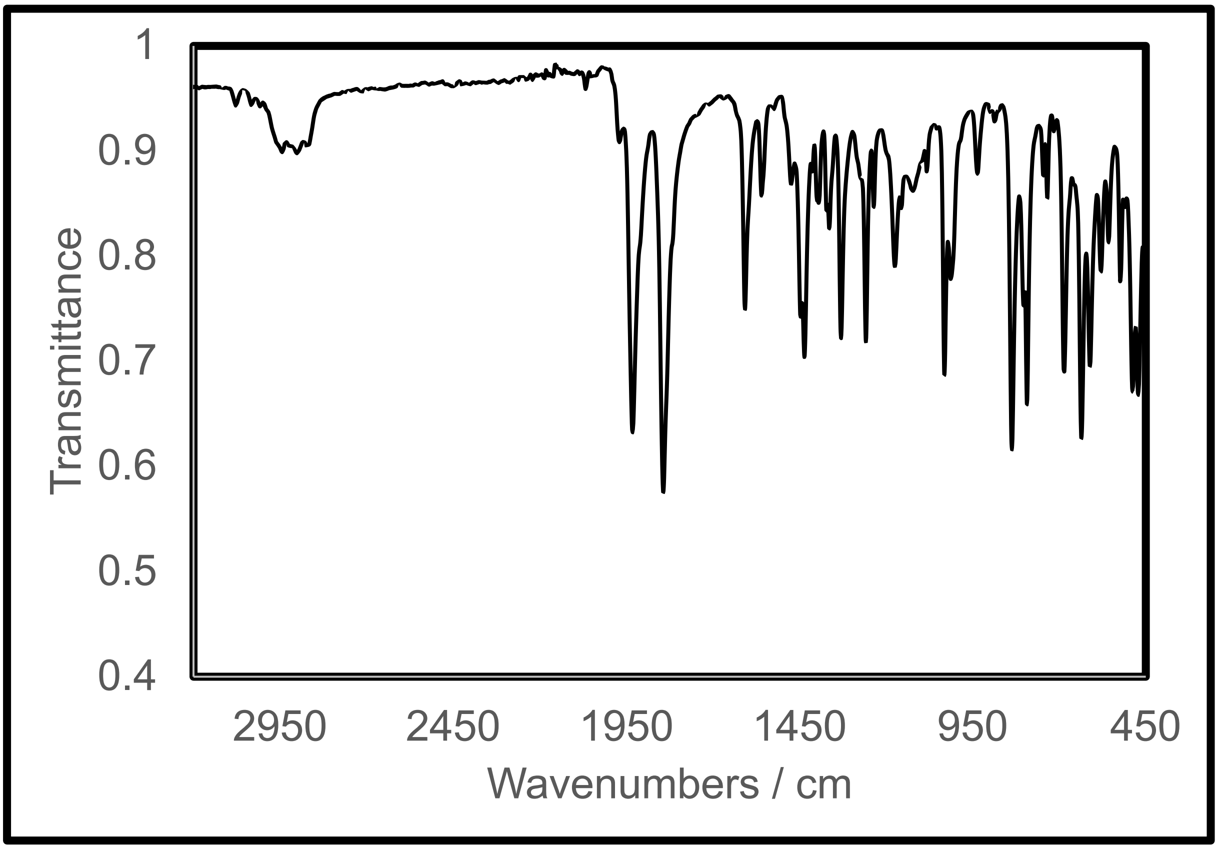

Figure S38: ATR IR spectrum of [Re(^{t}Bu-PONOP)Br(CO)$_2$].

[Re(^{t}Bu-PONOP)(CO)$_2$][BAr$^F{}_4$]

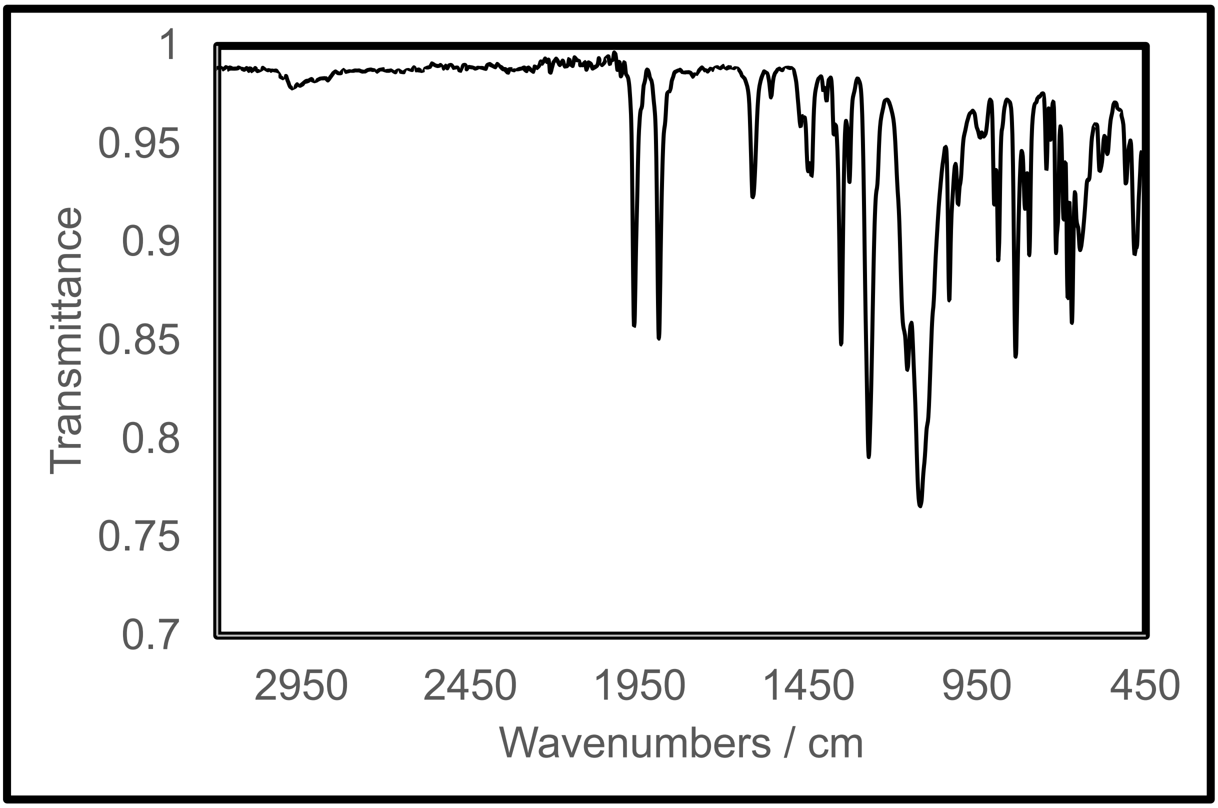

Figure S39: ATR IR spectrum of [Re(^{t}Bu-PONOP)(CO)$_2$][BAr$^F{}_4$].

[Re(^{t}Bu-PONOP)(CO)$_3$][BAr$^F{}_4$]

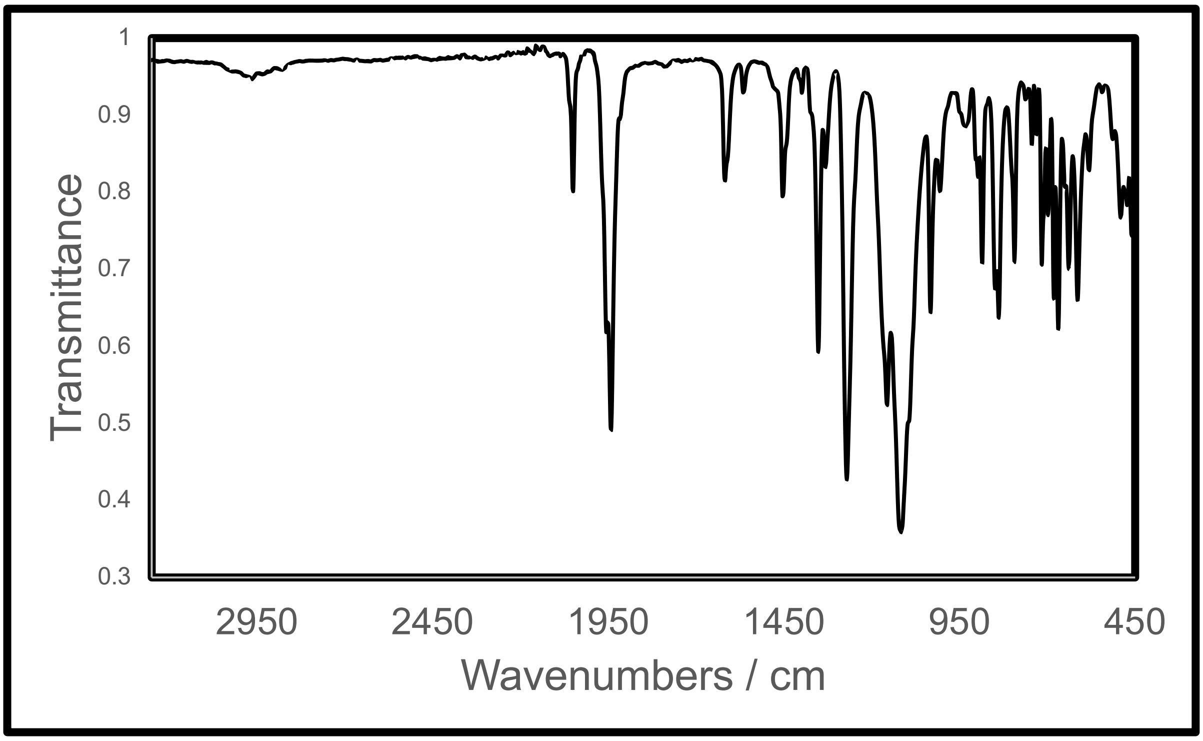

Figure S40: ATR IR spectrum of [Re(^{t}Bu-PONOP)(CO)$_3$][BAr$^F{}_4$] formed from addition of CO to [Re(^{t}Bu-PONOP)(CO)$_2$][BAr$^F{}_4$] in the solid-state.

S2. MicroED Methods

Sample Preparation

[Mn(^{i}Pr-PONOP)(CO)$_2$(THF)][BAr$^F{}_4$] was lightly crushed with a spatula and deposited onto Quantifoil Cu R2/4 grids that had been assembled into autogrid cartridges. Grids were transferred into glass vials and transported to the microscope under an Ar atmosphere. The grids were then conductively cooled to liquid N_2 temperature and transferred into the cassette under a blanket of N_2 vapour before loading into the TEM.

Data Collection

3DED data were collected at 80 K using a Thermo Scientific Glacios TEM operated at 200 kV and equipped with a Ceta-D camera. In order to obtain a low flux whilst operating the microscope in nanoprobe mode the following illumination conditions were used: gun lens 8, spot size 11, 30 μm C2 aperture. This resulted in a parallel beam of ~1 μm diameter and flux of 0.06 – 0.07 e^- $Å^{-2}$ s^{-1}. A selected area aperture was not used. Data were acquired using EPU-D with the following settings: 2x binning, a rotation speed of 2° s^{-1} and an exposure time of 0.5 s. 19 datasets were collected from crystals across 2 duplicate grids.

Data Processing

All data were processed with DIALS.[6] Images recorded on the Ceta-D exhibit negative mean background values at high resolution which causes failures in background modelling so a pedestal of 64 ADU was added to every pixel value. The initial detector distance was fixed to 958.5 mm (calibrated using an aluminium powder diffraction calibration grid). 8 datasets were combined resulting in 95.3% complete data to 0.83 Å resolution. The strong reflections from each of the datasets were used for joint refinement of the detector distances and unit cell parameters of each of the 8 datasets. The mean refined detector distance 958.48(20) mm was identical within error to the initial estimate. The optimal unit cell parameters of the combined dataset were then refined by fitting calculated to observed 2θ values.

Data Solution

The structure was solved *ab initio* using SHELXT[7] and refined with SHELXL.[8] The electron scattering factors from Peng[9] were used in refinement. Anisotropic ADPs were refined for all non-hydrogen atoms. Hydrogen atoms were geometrically placed at the idealised (inter-nuclear) X-H distances defined in SHELXL for refinement of structures against neutron diffraction data[10] and allowed to ride on their parent atoms during refinement. Three CF_3 groups are disordered in the structure and were modelled in two positions with distance (C-F and F-F), similar-ADP and rigid-bond[11] restraints (SADI, SIMU and RIGU instructions) used. No other restraints were applied. An extinction parameter was refined. To convert map values (in $Å^{-2}$) to values of electrostatic potential in $eÅ^{-1}$ a conversion factor of 47.87801 $Å^2V$ (International Tables for Crystallography (2006) Volume C section 4.3.1.7) followed by a conversion factor of 1V = 0.069446154 $eÅ^{-1}$ (based on recommended values from CODATA 2018) was used.

S3. Single-Crystal X-Ray Diffraction

Single-crystal X-Ray diffraction data were collected on a Rigaku SuperNova diffractometer with Cu-K_α (λ = 1.54184 Å) radiation equipped with a nitrogen gas Oxford Cryosystems Cryostream unit[12] at the University of York. Diffraction images from raw frame data were reduced using the CrysAlisPro suite of programmes. The structures were solved using SHELXT[7] and refined by full convergence on F^2 against all independent reflections by full-matrix least-squares using SHELXL[13] (version 2018/3) through the Olex2 GUI.[14] All non-hydrogen atoms were refined anisotropically and hydrogen atoms were geometrically placed and allowed to ride on their parent atoms. Disorder of the -CF_3 groups on the $[BAr^F{}_4]^-$ anions and the THF ligand in $[Mn(^iPr\text{-PONOP})(CO)_2(THF)][BAr^F{}_4]$ was treated by introducing a split-site model and restraining the geometries and displacement parameters. Disordered solvent in $[Mn(^iPr\text{-PONOP})(CO)_2(THF)][BAr^F{}_4]$ was modelled isotropically. Distances and angles were calculated using the full covariance matrix. Crystallographic data are available free of charge via the Cambridge Crystallographic Data Centre, under deposition numbers 2271683-2271688, and 2271690.

S3a. Room Temperature Structure of [Re(tBu-PONOP)(CO)2][BArF4]

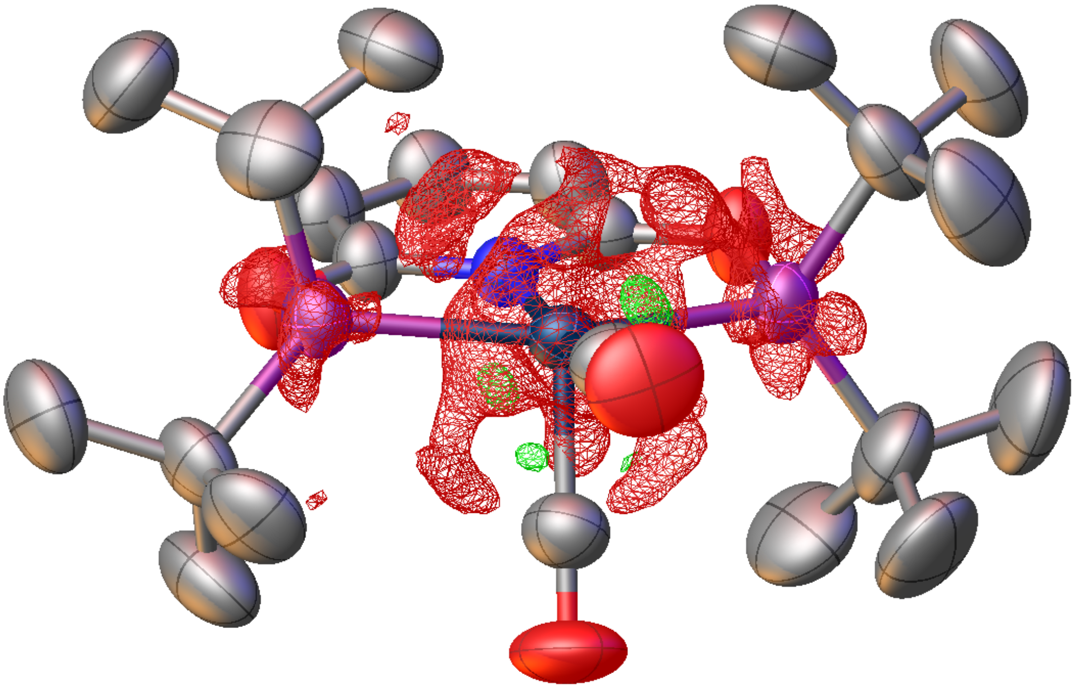

Figure S41: Structure of $[Re(^tBu\text{-PONOP})(CO)_2]^+$ cation as determined by single crystal X-Ray diffraction at 298 K. Hydrogen atoms omitted for clarity. A map of the residual electron density indicates no sign of positional disorder of the carbonyl environments in the solid-state.

S3b. Crystallographic Data Tables

Table S1: Selected crystallographic data for compounds **1** and **2**.

	[Mn(^{i}Pr-PONOP)(CO)$_3$][BAr$^F_4$] Recrystallised	[Mn(^{i}Pr-PONOP)(CO)$_2$(THF)][BAr$^F_4$] •½ hexane	[Mn(^{i}Pr-PONOP)(CO)$_3$][BAr$^F_4$] (microED)
CCDC Number	2271683	2271684	2271690
Formula	$C_{52}H_{43}BNO_5F_{24}P_2Mn$	$C_{58}H_{58}BF_{24}MnNO_5P_2$	$C_{52}H_{43}BNO_5F_{24}P_2Mn$
M_w	1345.56	1432.754	1345.56
Crystal System	Triclinic	Monoclinic	Triclinic
Space Group	P-1	P2$_1$/n	P-1
T / K	149.95(10)	150.00(10)	80(2)
a / Å	12.9543(6)	19.6838(4)	13.0245(6)
b / Å	14.1111(6)	23.8631(6)	14.1722(9)
c / Å	17.0692(8)	13.4251(3)	17.0849(12)
α / °	105.704(4)	90	105.823(5)
β / °	106.445(4)	90	106.400(5)
γ / °	97.358(4)	90	97.542(5)
V / Å^3	2809.2(2)	6306.0(2)	2835.6(3)
Z	2	4	2
ρ_{calc} / g cm^{-3}	1.591	1.509	1.576
μ / mm^{-1}	3.613	3.254	0.000
2Θ range / °	7.246 – 142.247	7.41 – 142.54	0.092 – 1.732
Reflns collected	20116	44881	43604
R_{int}	0.0302	0.0675	0.1748
Completeness / %	100	100	95.3
Data/restr/param	10626 / 157 / 895	12038 / 121 / 953	9903 / 339 / 868
R_1 [I > 2σ(I)]	0.0415	0.0759	0.1514
wR_2 [all data]	0.1109	0.2171	0.4048
GooF	1.043	1.014	1.028
Largest pk/hole / eÅ^{-3}	0.67 / -0.47	0.86 / -0.88	0.20 / -0.23
Flack param	-	-	-

Table S2: Selected crystallographic data for compound **3** and [Re(^{t}Bu-PONOP)Br(CO)$_2$]

	[Re(^{t}Bu-PONOP)Br(CO)$_2$]	[Re(^{t}Bu-PONOP)(CO)$_2$][BAr^F$_4$] 110 K	[Re(^{t}Bu-PONOP)(CO)$_2$][BAr^F$_4$] 298 K
CCDC Number	2271685	2271686	2271687
Formula	$C_{23}H_{39}BrNO_4P_2Re$	$C_{55}H_{51}BF_{24}NO_4P_2Re$	$C_{55}H_{51}BF_{24}NO_4P_2Re$
M_w	721.60	1504.43	1504.43
Crystal System	Orthorhombic	Triclinic	Triclinic
Space Group	$Pna2_1$	P-1	P-1
T / K	110.05(10)	110.00(10)	298.0(2)
a / Å	24.3315(2)	12.74810(10)	12.87470(10)
b / Å	10.26090(10)	14.4679(2)	14.51620(10)
c / Å	11.21410(10)	17.4995(2)	17.7612(2)
α / °	90	107.7110(10)	106.7050(10)
β / °	90	94.0020(10)	93.6650(10)
γ / °	90	98.7510(10)	98.2320(10)
V / $Å^3$	2799.75(4)	3015.62(6)	3127.18(5)
Z	4	2	2
ρ_{calc} / g cm^{-3}	1.712	1.657	1.598
μ / mm^{-1}	11.464	5.526	5.329
2Θ range / °	7.266 – 153.866	7.07 – 153.976	6.978 – 153.96
Reflns collected	32487	36432	37887
R_{int}	0.0383	0.0401	0.0356
Completeness / %	100	100	100
Data/restr/param	5693 / 1 / 301	12178 / 77 / 889	12765 / 223 / 1029
R_1 [I > 2σ(I)]	0.0227	0.0407	0.0263
wR_2 [all data]	0.0597	0.1063	0.0671
GooF	1.092	1.051	1.058
Largest pk/hole / e$Å^{-3}$	1.17 / -0.96	1.80 / -2.28	0.30 / -0.86
Flack param	-0.034(6)	-	-

Table S3: Selected crystallographic data for compound **4**.

	[(^{t}Bu-PONOP)Re(CO)$_3$][BAr^F$_4$]
CCDC Number	2271688
Formula	$C_{56}H_{51}BF_{24}NO_5P_2Re$
M_w	1532.92
Crystal System	Triclinic
Space Group	P-1
T / K	109.95(10)
a / Å	12.9529(4)
b / Å	14.1717(3)
c / Å	16.8449(5)
α / °	97.370(2)
β / °	94.209(3)
γ / °	96.468(2)
V / Å^3	3034.88(15)
Z	2
ρ_{calc} / g cm^{-3}	1.677
μ / mm^{-1}	5.517
2Θ range / °	6.896 – 155.206
Reflns collected	33048
R_{int}	0.0935
Completeness / %	100
Data/restr/param	12259 / 0 / 823
R_1 [I > 2σ(I)]	0.0577
wR_2 [all data]	0.1584
GooF	1.082
Largest pk/hole / eÅ^{-3}	1.55 / -2.34
Flack param	-

S4. SEM Methods

The samples were affixed to an Al stub *via* a carbon pad and then sputter coated with 5 nm of Cu in a JEOL JFC-2300HR high resolution fine coater, equipped with a JEOL FC-TM20 for thickness control. The SEM images were acquired on a JEOL JSM-7800F prime, equipped with a Schottky (field-assisted) thermionic emitter, at the York JEOL Nanocentre. The signals were collected in an off-axis Everhart-Thornley detector with a positive bias, in LED mode 3, for attraction of both secondary and backscattered electrons. An objective lens aperture size of 30 μm was used with an accelerating voltage of 5 keV, resulting in a probe current of 0.1 nA. At the used working distance of 10 mm, with the aforementioned settings, the maximum resolution of the instrument is 3 nm. The images produced are 1280 x 960 pixels in resolution and were collected with a dwell time of 14 μs at each pixel.

S5. Computational Section

S5a. Computational Details

S5ai. Periodic DFT Calculations

Periodic DFT calculations were performed employing the Gaussian Plane Wave (GPW) formalism as implemented in the QUICKSTEP[15] module within the CP2K program suite (Version 5.0).[16] Initial coordinates for [Mn(^{i}Pr–PONOP)$(CO)_2$(THF)][BAr^F_4], **1,** [Mn(^{i}Pr–PONOP)$(CO)_3$][BAr^F_4], **2**, [Re(^{t}Bu–PONOP)$(CO)_2$][BAr^F_4], **3** and [Re(^{t}Bu–PONOP)$(CO)_3$][BAr^F_4], **4**, were obtained from the experimental crystallographic data. For structure **1** the hexane molecule present in the experimental unit cell was removed and periodic DFT optimizations resulted in good agreement with the experimental structure (see Figure S43) The proto–structures **2*** and **4*** were generated from the unit cell of structures **1** and **3** via THF/CO substitution and CO addition at all metal centres respectively. Periodic boundary conditions (PBC) were applied throughout in combination with fixed unit cell parameters obtained from experiment. Molecularly optimized basis sets of double–ζ quality plus polarization in their short–range variant (DZVP–MOLOPT–SR–GTH)[17] were used on all atomic species. The interaction between the core electrons and the valence shell (Mn: 15, Re: 15, F: 7, O: 6, N: 5, P: 5, C: 4, B: 3, H: 1 electrons) was described by Goedecker–Teter–Hutter (GTH) pseudo potentials.[18-20] Geometry optimisations employed the PBE GGA functional[21] and included dispersion effects via Grimme's D3 correction.[22] The auxiliary plane wave basis set was truncated at a cutoff of 500 Ry. The maximum force convergence criterion was set to 10^{-4} Eh·Bohr^{-1}, whilst default values were used for the remaining criteria. The convergence criterion for the self–consistent field (SCF) accuracy was set to 10^{-7} Eh and 10^{-8} Eh for geometry optimizations. The electronic energies were used directly without further corrections. The Brillouin zone was sampled using the Γ–point.

Cartesian coordinates in Å of the central cations surrounded by the arrays of anions are provided as a separate XYZ file. Individual ion–pair geometries were taken from these structures without further modification. Good agreement between computed and experimental structures were obtained (see Figures S43-S46).

S5aii. Computational Details for Molecular DFT Calculations

Inter–ion interactions were analysed between one central metal cation and five nearest–neighbour surrounding [BAr^F_4]$^-$ anions, where ion–pair geometries were extracted from the periodic–DFT optimised structures. The ion–pair interaction energies were computed using Gaussian16 (Revision A.03) and employed the PBE functional with and without Grimme's D3 dispersion correction.[23] Mn and Re centres were described with Stuttgart pseudopotentials and associated basis sets[24] while 6–31G** basis sets were employed for the other atoms.[25, 26] The transition state for CO exchange in the [Re(^{t}Bu–PONOP)$(CO)_2$]$^+$ system was modelled and verified by the appearance of one imaginary frequency in the vibrational analysis. IRC calculations showed this transition state linked to two equivalent forms of the ground state structure, i.e. the [Re(^{t}Bu–PONOP)$(CO)_2$]$^+$ cation. Quantum theory of atoms in molecules (QTAIM) analysis[27] employed AIMALL program[28] and used the extended wavefunction format. Independent gradient model calculations were run with Multiwfn[29] with the Hirshfeld partitioning scheme (IGMH method).[30] Surfaces were visualised with VMD.[31]

S5b. QTAIM Analysis of the [Re(tBu-PONOP)(CO)2]+ molecular cation

The molecular graph for this species is shown in Figure S42 with bond critical points (BCPs) shown as green spheres. Bond paths with ρ(r) < 0.001 a.u. and ring critical points are omitted for clarity. Bond paths signalling weak H···H and O···H interactions were seen (see table of associated BCP metrics) but no evidence for any C–H···Re bond paths that would be associated with an agostic interaction was computed. Note that the atom labelling scheme provided here differs to that in the main manuscript.

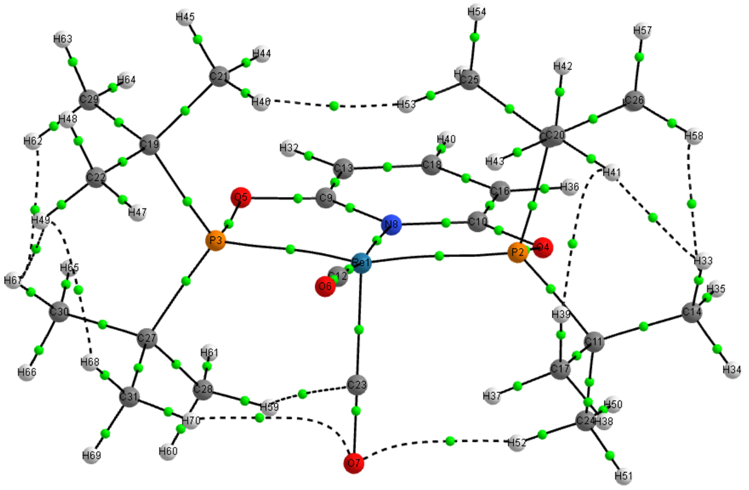

Bond Path	ρ(r)	$\nabla^2\rho$(r)	H	DI
H46–H53	0.007842	+0.025988	0.001474	0.018591
H62–H67	0.010281	+0.039383	0.002173	0.020331
H49–H67	0.006015	+0.021849	0.001330	0.014432
H49–H68	0.007544	+0.027752	0.001662	0.016084
O7–H52	0.008881	+0.032559	0.001326	0.032930
O7–H70	0.008597	+0.034345	0.001737	0.028607
C23–H59	0.010277	+0.039273	0.002003	0.022424
H39–H41	0.007689	+0.028718	0.001718	0.014352
H33–H58	0.008880	+0.034180	0.002000	0.015712
H33–H41	0.007966	+0.028626	0.001576	0.020127

Figure S42: Molecular graph for the [Re(^{t}Bu–PONOP)(CO)$_2$]$^+$ cation along with associated BCP metrics for H···H and O···H bond paths. All data are in atomic units: ρ =e/bohr^3; ∇^2ρ =e/bohr^5; H = hartree/bohr^3, q=e.

S5c. Comparison of experimental and computed structures of 1-4.

S5ci. [Mn(^{i}Pr–PONOP)(CO)$_2$(THF)][BAr$^F{}_4$], 1

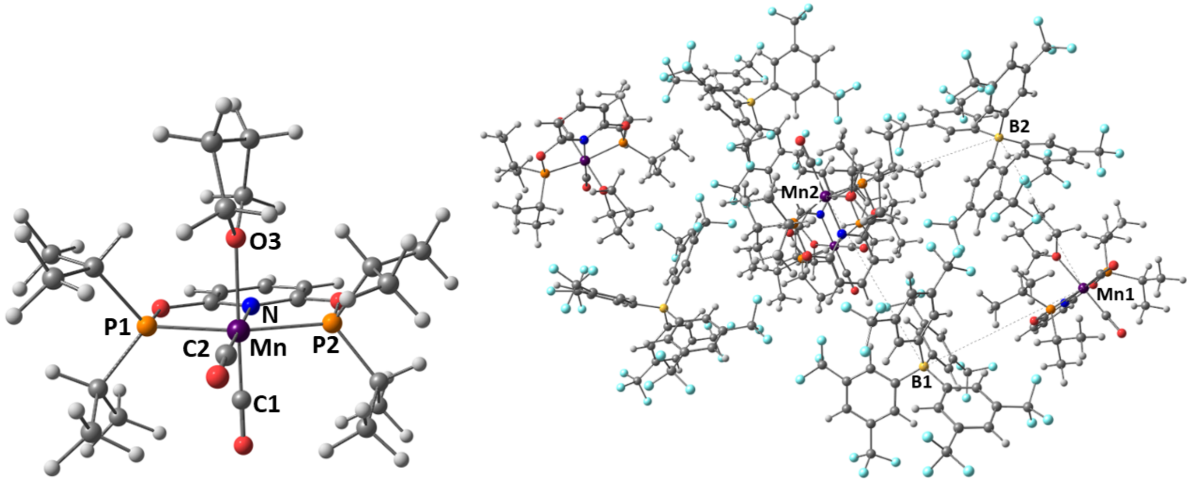

Internuclear Distance (Å)	Experimental	Periodic–DFT
Mn–C1	1.781 (5)	1.741
Mn–C2	1.789 (5)	1.773
Mn–N	2.027 (4)	2.040
Mn–O3	2.146 (4)	2.238
Mn–P1	2.2529 (14)	2.245
Mn–P2	2.2569 (14)	2.244
B1···Mn1	9.801 (5)	9.810
B2···Mn1	9.011 (6)	9.022
B1···Mn2	9.545 (6)	9.551
B2···Mn2	10.301 (5)	10.319

Figure S43: Comparison of experimental and computed distances in **1** with the labelling of key atoms in the **[Mn(^{i}Pr–PONOP)(CO)$_2$(THF)]$^+$** cation and the full unit cell as indicated.

S5cii. [Mn(^{i}Pr–PONOP)$(CO)_3$][BAr^{F}_4], 2

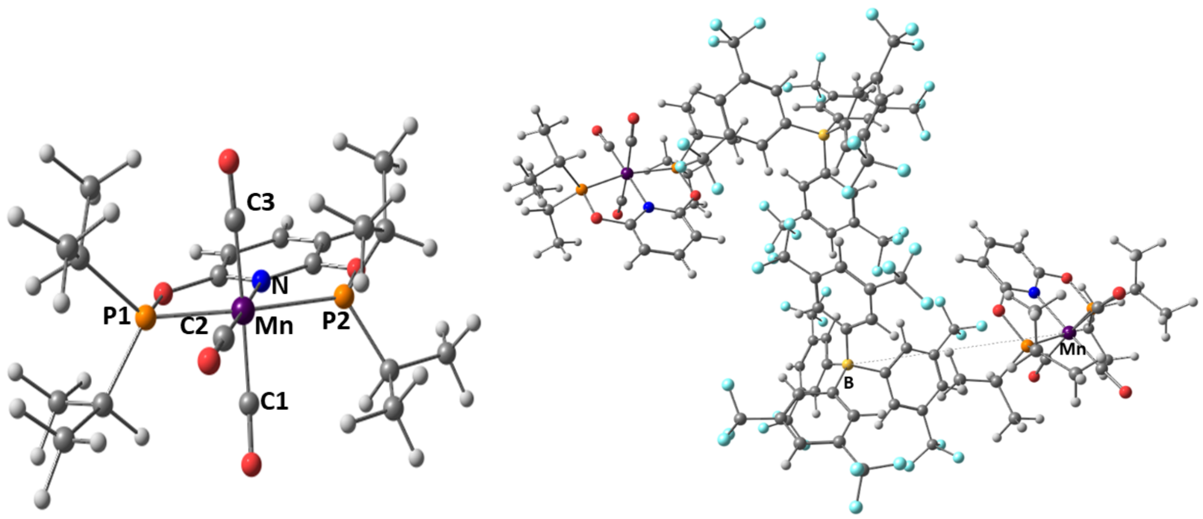

Internuclear Distance (Å)	Experimental	Periodic–DFT
Mn–C1	1.864 (8)	1.826
Mn–C2	1.79 (1)	1.826
Mn–N	2.073 (8)	2.062
Mn–C3	1.864 (8)	1.826
Mn–P1	2.258 (6)	2.251
Mn–P2	2.272 (6)	2.252
B···Mn	8.893 (11)	8.813

Figure S44: Comparison of experimental and computed distances in **2** with the labelling of key atoms in the **[Mn(^{i}Pr–PONOP)$(CO)_3$]$^{+}$** cation and the full unit cell as indicated.

S5cii. [Re(^{t}Bu–PONOP)(CO)$_2$][BAr$^F{}_4$], 3

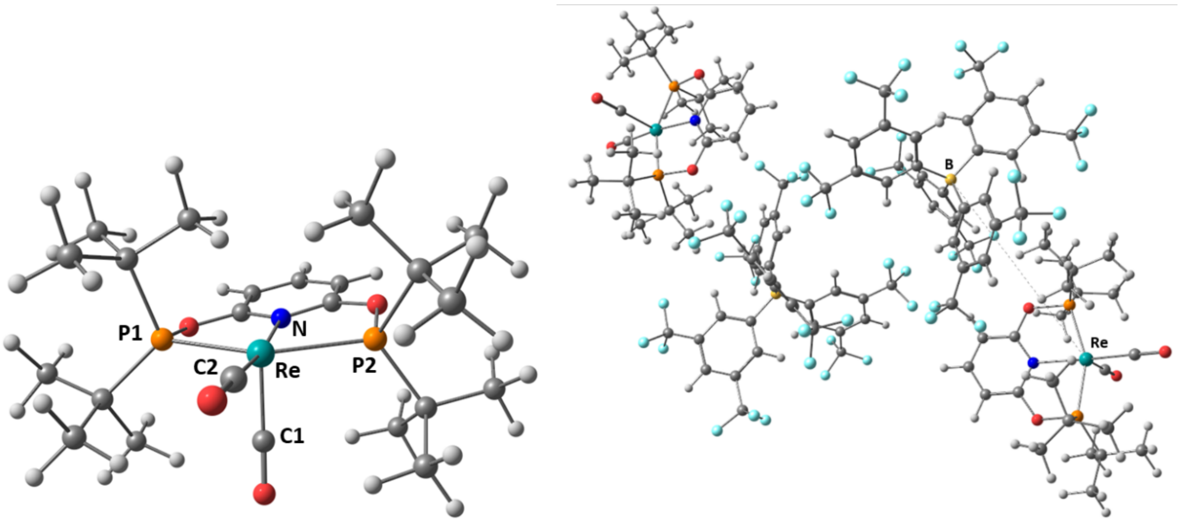

Bond Length (Å)	Experimental	DFT
Re–C1	1.863 (4)	1.837
Re–C2	1.927 (4)	1.908
Re–N	2.150 (3)	2.161
Re–P1	2.3541 (9)	2.364
Re–P2	2.3845 (9)	2.394
B···Re	9.112 (4)	9.126

Figure S45: Comparison of experimental and computed distances in **3** with the labelling of key atoms in the **[Re(^{t}Bu–PONOP)(CO)$_2$]$^+$**, cation and the full unit cell as indicated.

S5ciii. [Re(^{t}Bu–PONOP)(CO)$_3$][BAr$^F{}_4$], 4

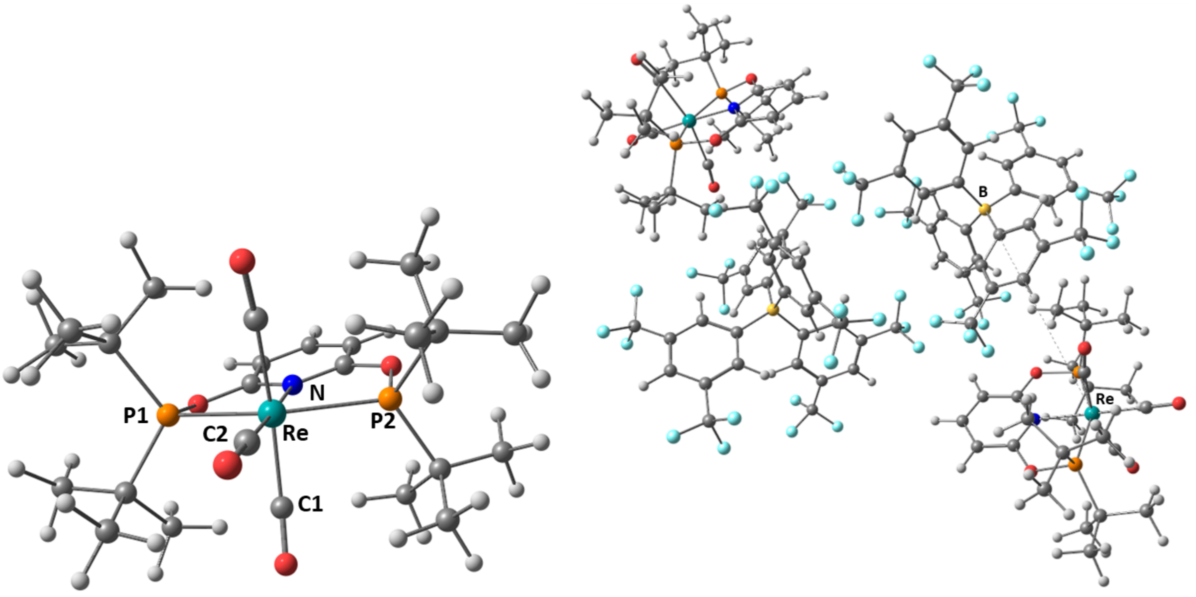

Bond Length (Å)	Experimental	DFT
Re–C1	1.982 (7)	1.968
Re–C2	1.940 (6)	1.911
Re–N	2.173 (4)	2.193
Re–P1	2.4170 (12)	2.439
Re–P2	2.4225 (12)	2.439
Re–C3	2.027 (6)	1.978
B···Re	8.701 (6)	8.712

Figure S46: Comparison of experimental and computed distances in **4** with the labelling of key atoms in the **[Re(^{t}Bu–PONOP)(CO)$_3$]$^+$,** cation and the full unit cell as indicated.

S5d. IGMH Plots

S5di. [Mn(^{i}Pr–PONOP)(CO)$_2$(THF)][BAr$^{F}_4$], 1, and [Mn(^{i}Pr–PONOP)(CO)$_3$][BAr$^{F}_4$], 2*

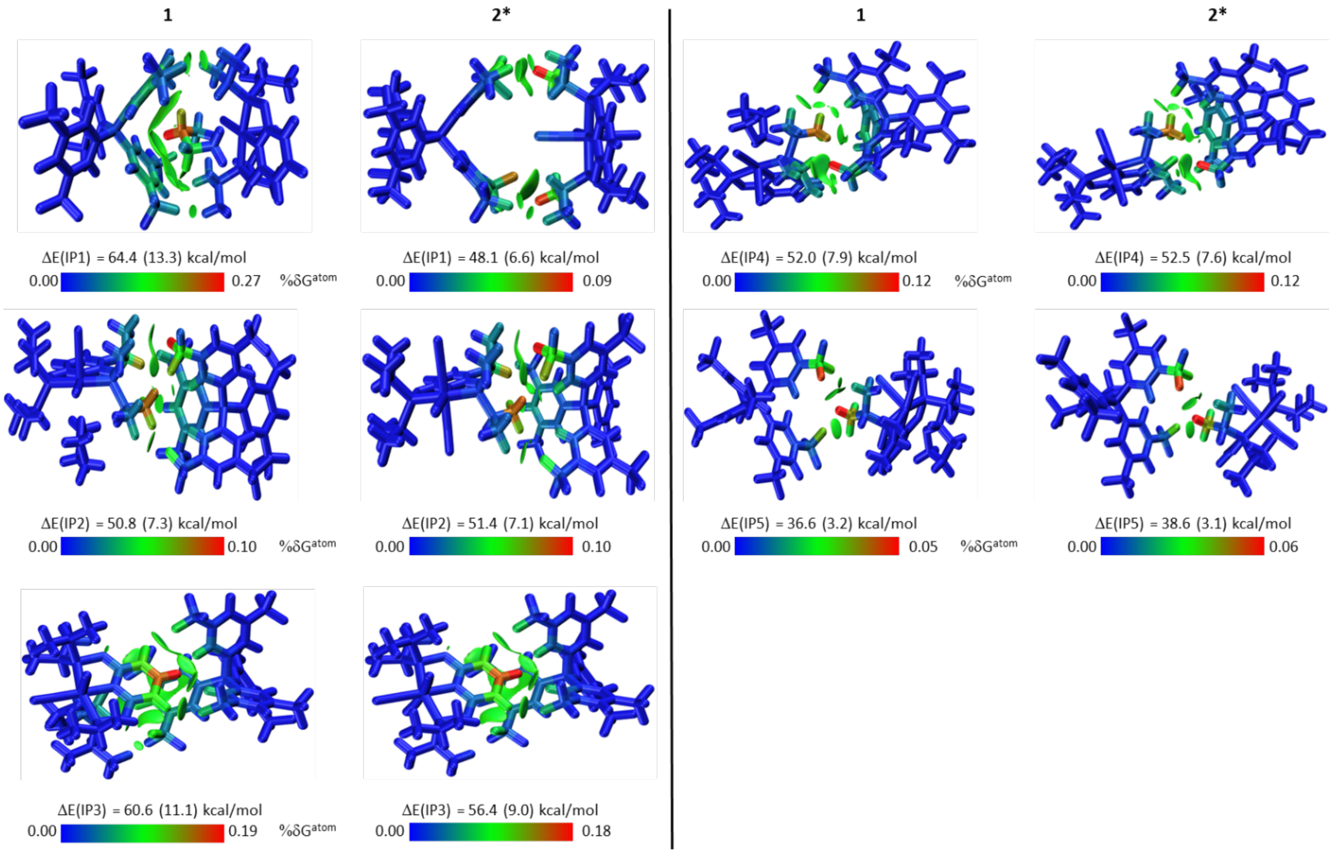

Figure S47: IGMH plots for IP1–IP5 in **1** and its equivalent in **2*** after THF/CO substitution. Cations and anions are defined as separate fragments; sign(λ_2)ρ–coloured isosurfaces are plotted with δG^{inter} = 0.003 a.u; relative atomic contributions coloured by $\%\delta G^{atom}$. The ion–pair interaction energies are also indicated (kcal/mol) with the contribution from dispersion in brackets.

S5dii. [Mn(^{i}Pr–PONOP)(CO)$_3$][BAr$^F{}_4$], 2

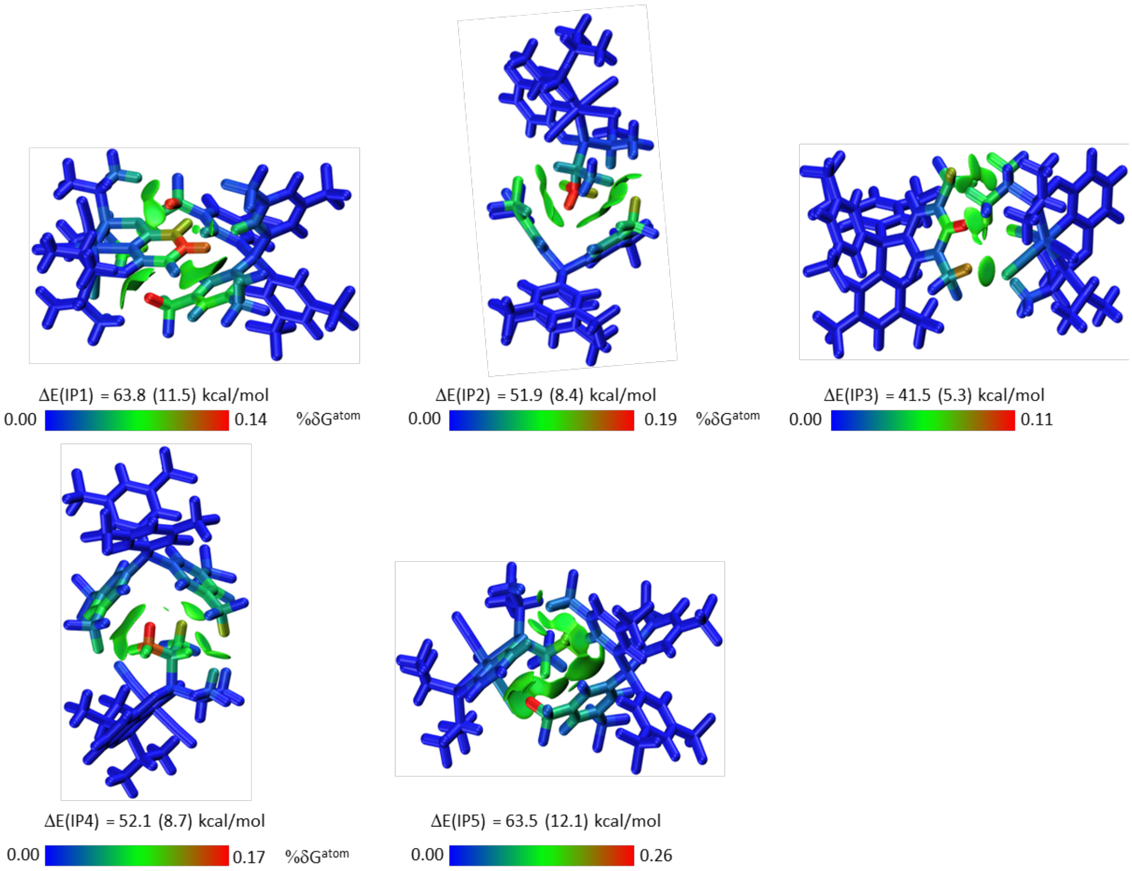

Figure S48: IGMH plots for IP1–IP5 in **2**. Cations and anions are defined as separate fragments; sign(λ_2)ρ–coloured isosurfaces are plotted with δG^{inter} = 0.003 a.u; relative atomic contributions coloured by %δG^{atom}. The ion–pair interaction energies are also indicated (kcal/mol) with the contribution from dispersion in brackets.

S5diii. [Re(^{t}Bu–PONOP)(CO)$_2$][BAr$^F{}_4$], 3 and [Re(^{t}Bu–PONOP)(CO)$_3$][BAr$^F{}_4$], 4*

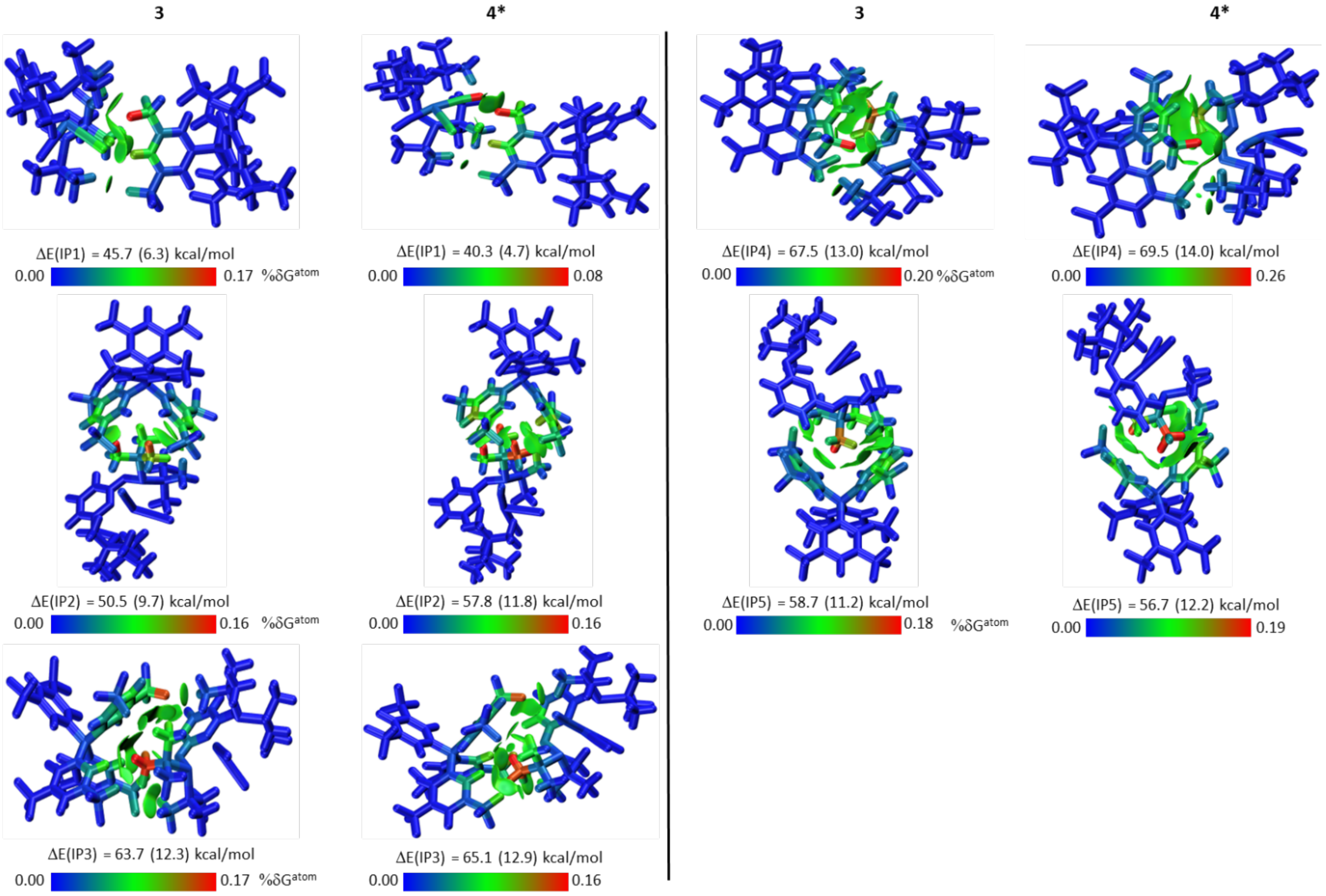

Figure S49: IGMH plots for IP1–IP5 in **3** and its equivalent in **4*** after THF/CO substitution. Cations and anions are defined as separate fragments; sign(λ_2)ρ–coloured isosurfaces are plotted with δG^{inter} = 0.003 a.u; relative atomic contributions coloured by %δG^{atom}. The ion–pair interaction energies are also indicated (kcal/mol) with the contribution from dispersion in brackets.

S5div. $[Re(^{t}Bu\text{–}PONOP)(CO)_3][BAr^{F}_4]$, 4

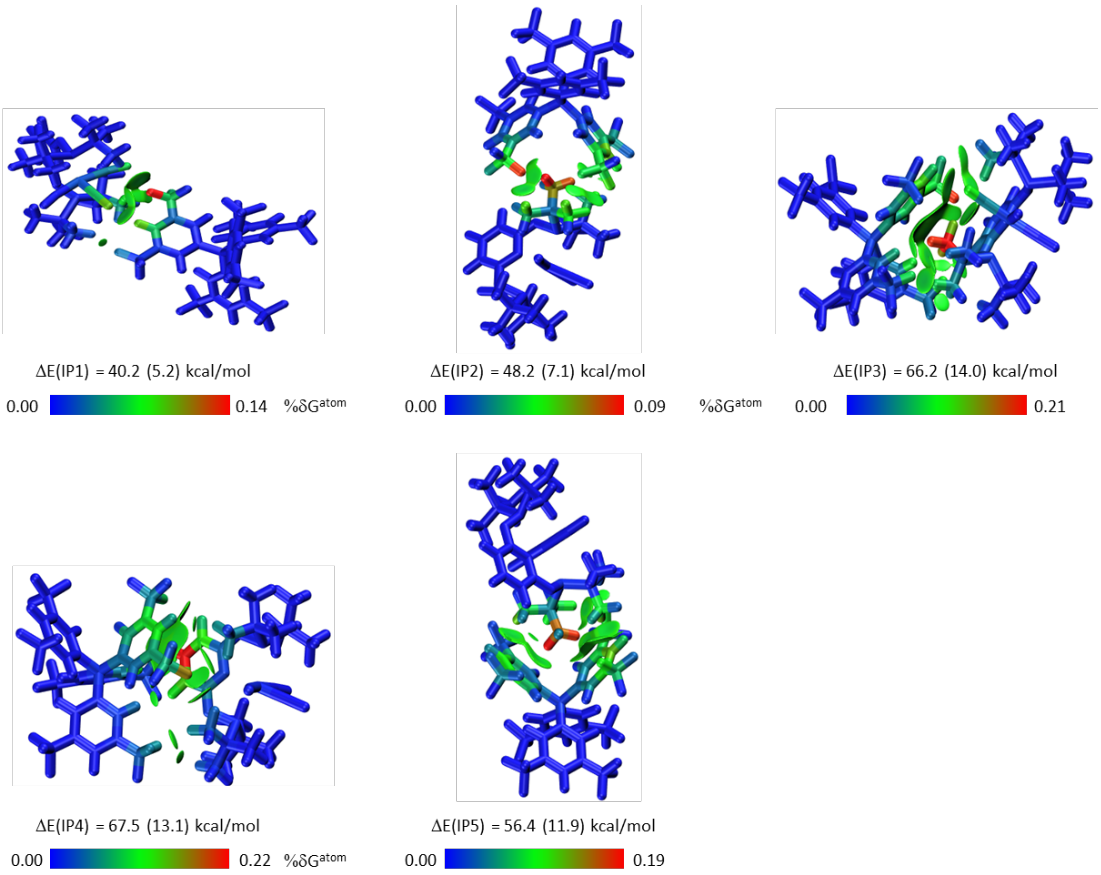

Figure S50: IGMH plots for IP1–IP5 in **4**. Cations and anions are defined as separate fragments; sign(λ_2)ρ–coloured isosurfaces are plotted with δG^{inter} = 0.003 a.u; relative atomic contributions coloured by %δG^{atom}. The ion–pair interaction energies are also indicated (kcal/mol) with the contribution from dispersion in brackets.

S6. References

1. A. J. Martínez-Martínez and A. S. Weller, *Dalton Trans.*, 2019, **48**, 3551-3554.
2. A. Johnson, C. G. Royle, C. N. Brodie, A. J. Martínez-Martínez, S. B. Duckett and A. S. Weller, *Inorg. Chem.*, 2021, **60**, 13903-13912.
3. S. Kundu, W. W. Brennessel and W. D. Jones, *Inorg. Chem.*, 2011, **50**, 9443-9453.
4. A. M. Tondreau and J. M. Boncella, *Polyhedron*, 2016, **116**, 96-104.
5. M. W. Ndinguri, C. A. Black, A. L. Gosser, S. Reitz, C. Sutphin, F. R. Fronczek and L. E. Cormier, *Inorg. Chim. Acta*, 2021, **520**, 120291.
6. D. G. W. G. Winter, J. M. Parkhurst, A. S. Brewster, R. J. Gildea, H. Gerstel, L. Fuentes-Montero, M. Vollmar, T. Michels-Clark, I. D. Young, N. K. Sauter and G. Evans *Acta Crystallogr. D.*, 2018, **74**, 85-97.
7. G. Sheldrick, *Acta Crystallogr. A*, 2015, **71**, 3-8.
8. G. M. Sheldrick, *Acta Crystallogr. C.*, 2015, **71**, 3-8.
9. L. M. Peng, *Micron*, 1999, **30**, 625-648.
10. T. Gruene, H. W. Hahn, A. V. Luebben, F. Meilleur and G. M. Sheldrick, *J. Appl. Crystallogr.*, 2014, **47**, 462-466.
11. A. Thorn, B. Dittrich and G. M. Sheldrick, *Acta Crystallogr. A*, 2012, **68**, 448-451.
12. J. Cosier and A. M. Glazer, *J. Appl. Crystallogr.*, 1986, **19**, 105-107.
13. G. Sheldrick, *Acta Crystallogr. A*, 2008, **64**, 112-122.
14. O. V. Dolomanov, L. J. Bourhis, R. J. Gildea, J. A. K. Howard and H. Puschmann, *J. Appl. Crystallogr.*, 2009, **42**, 339-341.
15. J. VandeVondele, M. Krack, F. Mohamed, M. Parrinello, T. Chassaing and J. Hutter, *Comput. Phys. Commun.*, 2005, **167**, 103-128.
16. J. Hutter, M. Iannuzzi, F. Schiffmann and J. VandeVondele, *WIREs Computational Molecular Science*, 2014, **4**, 15-25.
17. T. D. Kühne, M. Iannuzzi, M. Del Ben, V. V. Rybkin, P. Seewald, F. Stein, T. Laino, R. Z. Khaliullin, O. Schütt, F. Schiffmann, D. Golze, J. Wilhelm, S. Chulkov, M. H. Bani-Hashemian, V. Weber, U. Borštnik, M. Taillefumier, A. S. Jakobovits, A. Lazzaro, H. Pabst, T. Müller, R. Schade, M. Guidon, S. Andermatt, N. Holmberg, G. K. Schenter, A. Hehn, A. Bussy, F. Belleflamme, G. Tabacchi, A. Glöß, M. Lass, I. Bethune, C. J. Mundy, C. Plessl, M. Watkins, J. VandeVondele, M. Krack and J. Hutter, *J. Chem. Phys.*, 2020, **152**, 194103.
18. S. Goedecker, M. Teter and J. Hutter, *Phys. Rev. B*, 1996, **54**, 1703-1710.
19. C. Hartwigsen, S. Goedecker and J. Hutter, *Phys. Rev. B*, 1998, **58**, 3641-3662.
20. M. Krack, *Theor. Chem. Acc.*, 2005, **114**, 145-152.
21. J. P. Perdew, K. Burke and M. Ernzerhof, *Phys. Rev. Lett.*, 1996, **77**, 3865-3868.
22. S. Grimme, J. Antony, S. Ehrlich and H. Krieg, *J. Chem. Phys.*, 2010, **132**, 154104.
23. M. J. Frisch, G. W. Trucks, H. B. Schlegel, G. E. Scuseria, M. A. Robb, J. R. Cheeseman, G. Scalmani, V. Barone, G. A. Petersson, H. Nakatsuji, X. Li, M. Caricato, A. V. Marenich, J. Bloino, B. G. Janesko, R. Gomperts, B. Mennucci, H. P. Hratchian, J. V. Ortiz, A. F. Izmaylov, J. L. Sonnenberg, Williams, F. Ding, F. Lipparini, F. Egidi, J. Goings, B. Peng, A. Petrone, T. Henderson, D. Ranasinghe, V. G. Zakrzewski, J. Gao, N. Rega, G. Zheng, W. Liang, M. Hada, M. Ehara, K. Toyota, R. Fukuda, J. Hasegawa, M. Ishida, T. Nakajima, Y. Honda, O. Kitao, H. Nakai, T. Vreven, K. Throssell, J. A. Montgomery Jr., J. E. Peralta, F. Ogliaro, M. J. Bearpark, J. J. Heyd, E. N. Brothers, K. N. Kudin, V. N. Staroverov, T. A. Keith, R. Kobayashi, J. Normand, K. Raghavachari, A. P. Rendell, J. C. Burant, S. S. Iyengar, J. Tomasi, M. Cossi, J. M. Millam, M. Klene, C. Adamo, R. Cammi, J. W. Ochterski, R. L. Martin, K. Morokuma, O. Farkas, J. B. Foresman and D. J. Fox, *Journal*, 2016.
24. D. Andrae, U. Häußermann, M. Dolg, H. Stoll and H. Preuß, *Theor. Chim. Acta*, 1990, **77**, 123-141.
25. W. J. Hehre, R. Ditchfield and J. A. Pople, *J. Chem. Phys.*, 1972, **56**, 2257-2261.
26. P. C. Hariharan and J. A. Pople, *Theor. Chim. Acta*, 1973, **28**, 213-222.

27. R. F. W. Bader, *Atoms in molecules : a quantum theory*, Oxford : Clarendon press, 1990.
28. T. A. Keith, AIMAII, http://aim.tkgristmill.com).
29. T. Lu and F. Chen, *J. Comput. Chem.*, 2012, **33**, 580-592.
30. T. Lu and Q. Chen, *J. Comput. Chem.*, 2022, **43**, 539-555.
31. E. D'Oria and J. J. Novoa, *CrystEngComm*, 2008, **10**, 423-436.
